# A transient increase of HIF-1α during the G1 phase (G1-HIF) ensures cell survival under nutritional stress

**DOI:** 10.1038/s41419-023-06012-7

**Published:** 2023-07-27

**Authors:** Ratnal Belapurkar, Maximilian Pfisterer, Jan Dreute, Sebastian Werner, Sven Zukunft, Ingrid Fleming, Michael Kracht, M. Lienhard SCHMITZ

**Affiliations:** 1 Institute of Biochemistry, Justus-Liebig-University, Member of the German Center for Lung Research, Giessen, Germany; 2 Rudolf Buchheim Institute of Pharmacology, Justus-Liebig-University, Member of the German Center for Lung Research, Giessen, Germany; 3 Institute for Vascular Signalling, Goethe University, Frankfurt am Main, Germany; 4 German Center of Cardiovascular Research (DZHK), Partner site RheinMain, Frankfurt am Main, Germany

**Keywords:** Molecular biology, Biochemistry

## Abstract

The family of hypoxia-inducible transcription factors (HIF) is activated to adapt cells to low oxygen conditions, but is also known to regulate some biological processes under normoxic conditions. Here we show that HIF-1α protein levels transiently increase during the G1 phase of the cell cycle (designated as G1-HIF) in an AMP-activated protein kinase (AMPK)-dependent manner. The transient elimination of G1-HIF by a degron system revealed its contribution to cell survival under unfavorable metabolic conditions. Indeed, G1-HIF plays a key role in the cell cycle-dependent expression of genes encoding metabolic regulators and the maintenance of mTOR activity under conditions of nutrient deprivation. Accordingly, transient elimination of G1-HIF led to a significant reduction in the concentration of key proteinogenic amino acids and carbohydrates. These data indicate that G1-HIF acts as a cell cycle-dependent surveillance factor that prevents the onset of starvation-induced apoptosis.

## Introduction

Cell division requires major metabolic changes to enable duplication of the DNA and the cell mass [1]. Cell growth, division and metabolism are tightly coupled to provide biosynthetic precursors and ATP to allow for energy-consuming processes such as mitosis [2]. Throughout the cell cycle cells rewire metabolic circuits to allow (i) an increased glycolytic flux during G1, (ii) the production of nucleotide precursors for the synthesis of DNA and RNA during G1/S phase and (iii) an increased oxidative phosphorylation during mitosis [3, 4]. In yeast, more than 50% of the metabolome changes to satisfy periodic demands during the cell cycle, while in vertebrates mitochondria converge into a hyper-fused giant network during G1/S transition [5, 6]. Cell cycle-dependent changes in metabolism rely heavily on cyclin-dependent kinases (CDKs), which, in addition to their role as regulators of the cell cycle, also influence metabolic circuits [7–9]. During a G1 phase checkpoint known as *Start* in yeast and as the restriction point “R” in mammals, nutrient concentrations and cell size are sensed to allow irreversible entry into the cell cycle in the presence of suitable conditions [10, 11]. This sensing of nutrient availability is mediated by various enzymes including the kinases mTOR (mammalian target of rapamycin) or AMP-activated protein kinase (AMPK). The latter kinase functions as a cellular energy sensor and is rapidly activated by unfavorable conditions represented by low ATP/AMP ratios [12].

One of the AMPK substrates is the transcription factor HIF-1 [13], a heterodimer composed of HIF-1β and oxygen-regulated HIF-1α subunits [14]. The oxygen-dependent regulation of HIF-1 is achieved via its O_2_-dependent proline hydroxylation by prolyl-4-hydroxylases (PHDs) and subsequent degradation by the proteasome, a process that leads to a rapid decrease in HIF-1 levels under normoxic conditions [15]. At the same time, asparagine hydroxylation reduces transcription activation by precluding the binding of HIF-1α to the coactivator p300 [16]. HIF-1 can be also upregulated by a number of different oxygen-independent mechanisms that occur under physiological [17] and pathophysiological conditions, such as cancer [18, 19]. Increased HIF levels can result from various mechanisms including increased transcription of the *HIF1A* gene [20], elevation of protein stability for example by phosphorylation [21] and association with heat shock protein 90 kDa (HSP90) [22]. In addition, oxygen-independent HIF stabilization can involve mechanisms leading to reduced levels of degradative ubiquitination, for example by interaction with deubiquitinating enzymes [23] or by TNF receptor-associated factor 6 (TRAF6)-mediated addition of regulatory (K63-branched) ubiquitin chains [24]. Following its stabilization, the nuclear HIF-1 dimer associates with hundreds of binding sites to induce or repress transcription of its target genes [25, 26]. HIF-1 has also functions that are independent from DNA-binding, as it was shown for example that HIF-1α and HIF-2α can interact with the MCM DNA helicase complex [27] and the helicase loading factor cell division cycle 6 (CDC6) [28], thus causing decreased DNA replication. Further non-transcriptional functions include the γ-secretase activating function of HIF and the mitochondrial association of a fraction of HIF [29, 30].

Here we set out to investigate the regulation of HIF-1α during the cell cycle and discovered that its levels increase transiently during the G1 phase. Transiently activated G1-HIF acts as a surveillance factor that prevents the onset of apoptosis likely due to its ability to ameliorate the supply of metabolites under unfavorable metabolic conditions.

## Results

### HIF-1α is transiently stabilized during the G1 phase of the cell cycle

**Fig. 1:**
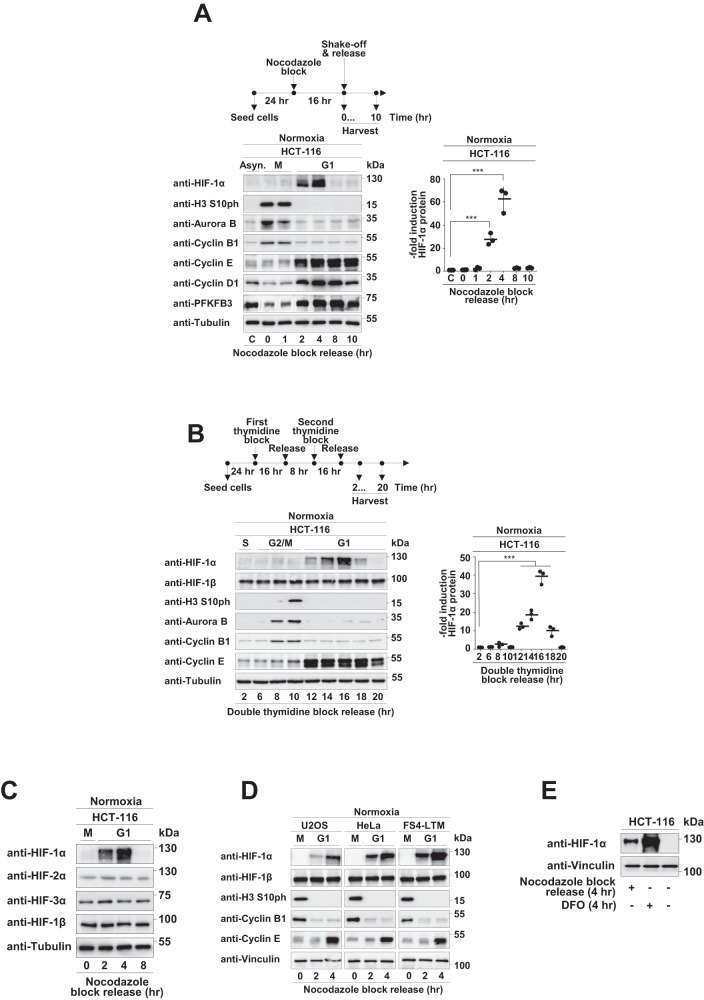
Cell cycle-dependent expression levels of HIF-1α in normoxia. **A** The top visualizes the experimental protocol indicating that HCT-116 cells were arrested in prometaphase by treatment with nocodazole (0.1 µg/ml) for 16 h and released into M phase by shaking off the non-adherent cells. Cells were collected at the indicated time points, and lysates were analyzed by Western blotting using the indicated antibodies detecting HIF-1α and further cell cycle regulated proteins. The positions of molecular weight markers are indicated. The right part shows a quantification of normalized G1-HIF protein levels (**B**) HCT-116 cells were synchronized by a double thymidine block (2 mM thymidine), washed, and released into the S phase as schematically indicated at the top. Cells were collected at the indicated time points and analyzed by immunoblotting for the expression of HIF-1α and cell cycle markers. The right part shows a quantitative analysis of G1-HIF protein expression. **C** The experiment was performed as in (**A**) with the difference that all DNA-binding members of the HIF family were detected. **D** The indicated cells were synchronized via nocodazole block and release, followed by detection of G1-HIF and the cell cycle regulators by immunoblotting. **E** HCT-116 cells were synchronized via nocodazole block/release or treated with DFO (10 µM) as shown, followed by the analysis of HIF-1α expression by Western blotting. Quantifications show the mean ± SD, as calculated using the one-way ANOVA with Tukey multiple comparisons test (*** = *P* ≤ 0.001, *n* = 3).

As nutrient availability regulates metabolic checkpoints of the cell cycle, we investigated whether also HIF-1α protein levels are subject to cell cycle-dependent changes. To address this question, we employed human diploid HCT-116 colon cancer cells which can be conveniently synchronized and are widely used in cell cycle research [31, 32]. Cells were synchronized by inducing arrest during early mitosis using nocodazole, followed by mitotic shake-off and release into the cell cycle for different periods of time, as schematically shown in Fig. 1A. Cell extracts were prepared and analyzed by Western blotting for the occurrence of HIF-1α and further cell cycle regulatory proteins. Successful cell synchronization was ensured by controls revealing the time-dependent reduction of mitotic markers (H3 S10 phosphorylation, Aurora B and Cyclin B1), increased levels of Cyclin D and E (Fig. 1A) and of PFKFB3 (6-phosphate fructose-2-kinase/fructose-2,6-bisphosphatase-3), a regulator of carbohydrate metabolism that is also increased in G1 [33]. Furthermore, cell synchronization was confirmed by flow ctyometric analysis (Fig. S1A). These experiments showed a transient increase of HIF-1α protein levels during the G1 phase peaking between 2 and 4 h after release from the nocodazole block, while the levels of the HIF-1α protein were very low in the other cell cycle phases (Fig. 1A). The transient stabilization of HIF-1α during the G1 phase was not due to changes at the mRNA level (Fig. S1B) and was also detected in HCT-116 cells synchronized by release from a double thymidine block (Fig. 1B).

The transient increase during G1 phase was restricted to the dynamically regulated HIF-1α protein, as there were no changes of the expression levels of the other HIF family members (Fig. 1C). G1-HIF was also detected in all tested tumor cell lines and in non-transformed primary human fibroblasts, as revealed by immunoblotting (Fig. 1D) and its quantification (Fig. S1C). To compare the levels of G1-HIF with that of oxygen-dependent HIF, Western blot experiments were performed using extracts from synchronized cells and those treated with the PHD inhibitor deferoxamine (DFO). These results show that the levels of G1-HIF are way lower as compared to HIF amounts occurring after PHD inhibition (Fig. 1E) or hypoxia (data not shown).

### G1-HIF protects cells from death caused by metabolic stress

**Fig. 2:**
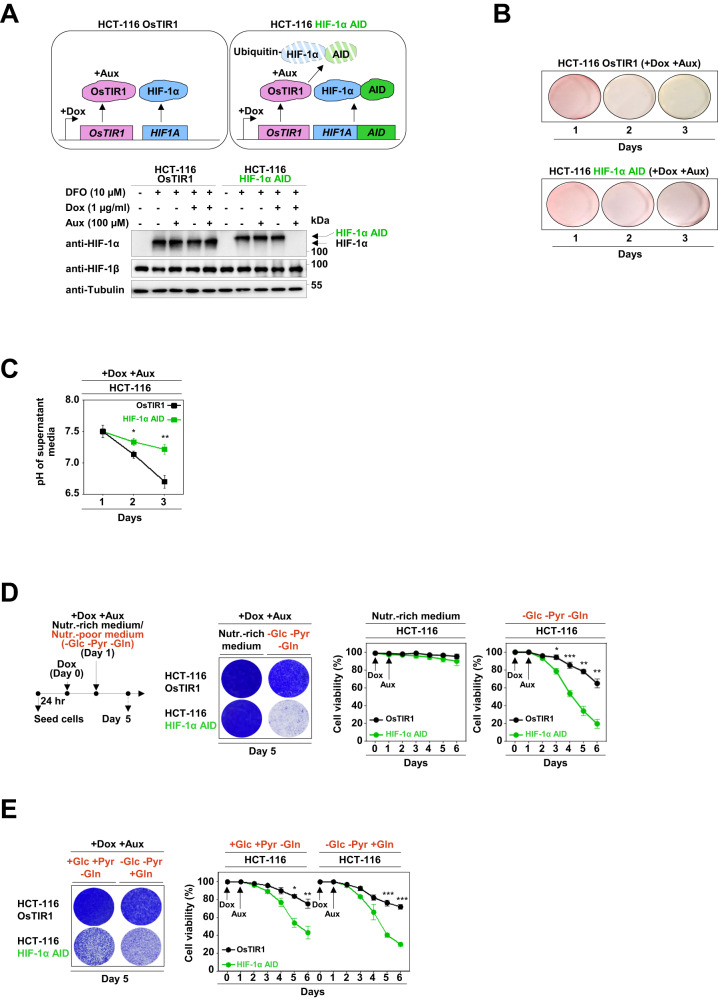
G1-HIF protects from cell death induced by nutritional stress. **A** The experimental strategy for fusion of the AID to the C-terminus of HIF-1α is schematically depicted at the top. The functional characterization was done by treatment of the indicated cells with Dox (1 µg/ml) for 24 h (to enable expression of OsTIR1), followed by treatment with DFO (10 µM) for 6 h (to generate detectable HIF-1α protein levels) and subsequent treatment with Aux (100 µM) for 30 min. Cell lysates were analyzed for HIF-1 protein expression with specific antibodies. **B** The indicated cells were pre-treated with Dox for 24 h and then cultivated in the presence of Aux. The color changes in the cell culture media over several days are displayed. **C** Cells were treated as in (**B**) and the pH of the cell culture media was determined. **D** The experimental scheme is shown in the left. HCT-116 OsTIR1 and HCT-116 HIF-1α AID cells were pre-treated for 1 day with Dox to allow first the expression of OsTIR1. Then cells were further cultured in nutrient-rich or nutrient-poor medium lacking glucose (Glu), pyruvate (Pyr) and glutamine (Gln) in the presence of Dox and Aux as shown. Five days after the addition of Dox the remaining attached cells were washed with PBS and stained using crystal violet (middle). Cells were treated as shown above and cell viability was examined each day using a LUNA automated cell counter. The mean ± SD of three independent biological replicates is shown (right). **E** The experiments were done as in (**D**) with the difference that differently composed nutrient-poor media were used. Quantifications show the mean ± SD, as calculated using the two-way ANOVA with Bonferroni multiple comparisons test (* = *P* ≤ 0.05, ** = *P* ≤ 0.01, *** = *P* ≤ 0.001, *n* = 3).

**Fig. 3:**
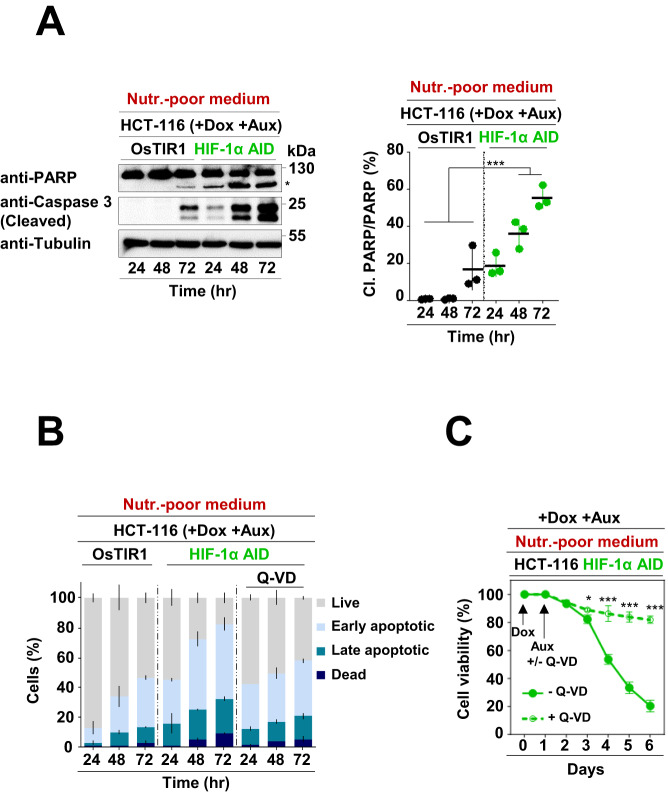
The absence of G1-HIF leads to apoptosis under nutrient-poor conditions. **A** Left: The indicated cells were pre-treated for 1 day with Dox (1 µg/ml) and then further cultured in a nutrient-poor medium in the presence of Aux (100 µM) for several periods. The cleavage of caspase-3 and its substrate PARP were detected by immunoblotting. The cleaved form of PARP is shown by an asterisk. Right: Quantification of PARP cleavage, data are shown as mean ± SD and analyzed with one-way ANOVA with Tukey multiple comparisons test (*** = *P* ≤ 0.001, *n* = 3). **B** HCT-116 OsTIR1 and HCT-116 HIF-1α AID cells were treated and processed as in (**A**), followed by analysis of Annexin V binding and PI staining via flow cytometry. **C** HCT-116 HIF-1α AID cells were treated and processed as in (**A**) in the presence/absence of the pan-caspase inhibitor Q-VD (20 µM). At the indicated time points, cells were collected, and cell viability was measured using a LUNA-II™ automated cell counter. Cell viability of untreated cells was set to 100%, data are shown as mean ± SD and analyzed with two-way ANOVA with Bonferroni multiple comparisons test (* = *P* < 0.05, *** = *P* < 0.001, *n* = 3).

To study the function of G1-HIF, we genetically engineered HCT-116 cells to allow the fast elimination of HIF-1α using an Auxin-inducible degron (AID) system (Fig. S2A, B). These cells (designated as HCT-116 HIF-1α AID cells) also express the doxycycline (Dox)-inducible ubiquitin E3 ligase TIR1 (transport inhibitor response 1 protein) from Oryza sativa (OsTIR1) [34], to allow the rapid elimination of HIF-1α after addition of Dox and the plant hormone Auxin (Aux) (Fig. 2A). The HIF-1α AID fusion protein was fully functional as evidenced by the intact hypoxia-induced expression of HIF target genes (Fig. S2C). When cultivating HCT-116 OsTIR1 and HCT-116 HIF-1α AID cells in the presence of Dox and Aux, we made the serendipitous observation that depletion of HIF-1α largely prevented the color change of DMEM medium that occurred when cells were grown for several days in the same medium (Fig. 2B). The difference in color change was not related to alterations in proliferation or cell cycle distribution of the cells (Fig. S2D), but was rather correlated with reduced acidification of the medium after G1-HIF depletion (Fig. 2C). Given that HIF-1α is an important regulator of metabolism under both hypoxic and normoxic conditions [35, 36], we determined whether the transient expression of the transcription factor affected cell viability under conditions of nutritional stress. To address this, HCT-116 HIF-1α AID cells and control cells were further grown in nutrient-rich DMEM (Dulbecco’s modified eagle medium) medium or nutrient-poor DMEM medium without supplementation of glucose, pyruvate and glutamine in the presence or absence of HIF-1α, as schematically shown in Fig. 2D. While the depletion of G1-HIF showed almost no reduction of cell viability in nutrient-rich medium, its elimination in nutrient-poor medium caused strong cell death, as revealed by crystal violet staining of the remaining attached cells. This effect was fully recapitulated by quantitative and time-resolved analysis of cell viability by dye exclusion (Fig. 2D), further controls ensured that neither Dox nor Aux had significant cytotoxic activities (data not shown). The protective effect of G1-HIF was also evident in medium lacking glucose and pyruvate or in the absence of glutamine (Fig. 2E), hinting that G1-HIF protects cells from death induced by various types of metabolic stress. Further experiments in nutrient-poor medium revealed no impact of G1-HIF on proliferation and cell cycle dynamics (Fig. S2E), senescence (Fig. S2F) or autophagy (Fig. S2G) at the analyzed time points, suggesting that G1-HIF maintained cell viability by other processes. Instead, we observed that the absence of G1-HIF in nutrient-poor medium increased the cleavage of Caspase-3 and its substrate poly (ADP-ribose) polymerase (Fig. 3A), both of which are indicative of apoptosis induction. Consistent with this, the depletion of G1-HIF in cells cultured in nutrient-poor medium increased the number of cells in early and late apoptosis (Fig. 3B). The latter effects were prevented by the pan-caspase inhibitor Quinoline-Val-Asp-difluorophenoxymethylketone (Q-VD) (Fig. 3C), indicating that G1-HIF is essential to prevent caspase-mediated apoptosis under conditions of starvation.

### G1-HIF regulates amino acid and carbohydrate homeostasis

**Fig. 4:**
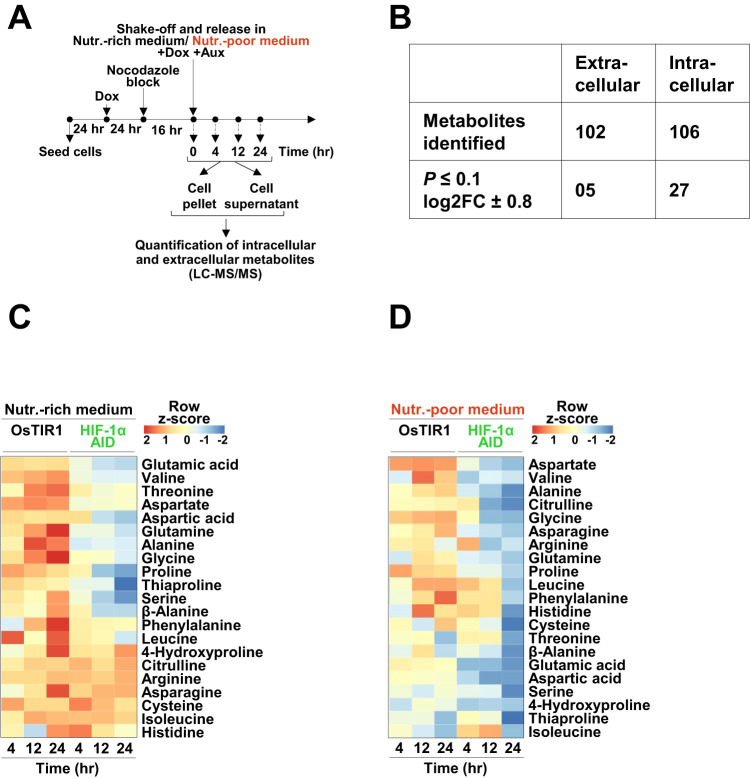
Regulation of amino acid homeostasis by G1-HIF. **A** Schematic illustration of the experimental workflow for metabolic profiling. Post nocodazole/block release, HCT-116 OsTIR1 and HCT-116 HIF-1α AID cells were treated as shown and harvested at different time points. Cell pellets and supernatants were separately analyzed using targeted LC-MS/MS. **B** Summary of metabolites identified by targeted metabolomics (threshold *P* value < 0.1 and ±LFC ≥ 0.8). Heat maps show the contribution of G1-HIF for intracellular amino acid levels for cells grown in nutrient-rich (**C**) and nutrient-poor (**D**) medium.

As the biological function of G1-HIF discovered here was only apparent after metabolic perturbation, we assessed its impact on cell metabolism. Therefore, HCT-116 HIF-1α AID and control cells were released from a nocodazole block in Auxin-containing nutrient-rich or nutrient-poor medium, followed by determination of the levels of extracellular and intracellular selected metabolites at various time points, as schematically shown in Fig. 4A. Targeted metabolic analyses (LC-MS/MS) was used to detect changes in amino-acid metabolism, the tricarboxylic acid cycle (TCA cycle) and the pentose-phosphate pathway. A total of 102 extracellular and 106 intracellular metabolites were detected of which 5 extracellular and 27 intracellular metabolites were differentially regulated in either one or both nutrient conditions during at least one phase of the cell cycle (Fig. 4B and Supplemental Table S1).

**Fig. 5:**
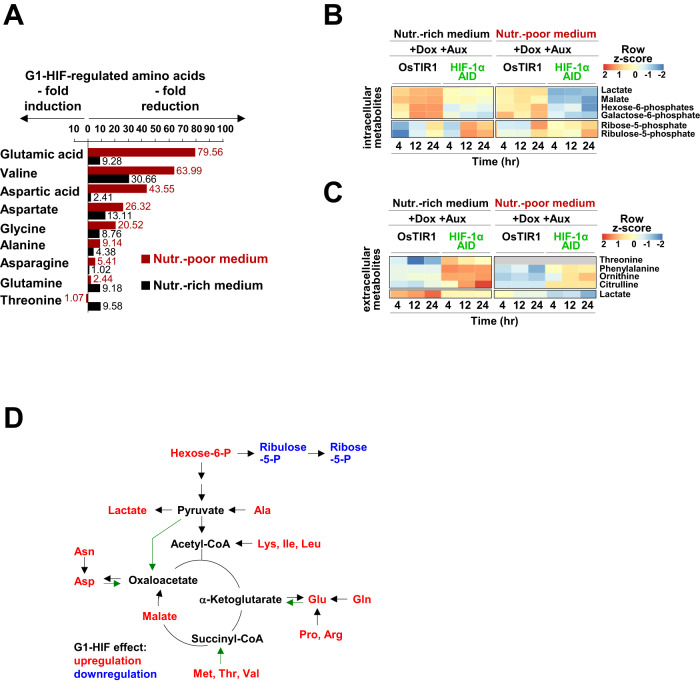
Regulation of metabolites by G1-HIF. **A** Ratios of the mean values of G1-HIF-dependent amino acids are shown. The heat maps show G1-HIF-dependent dynamic changes of the indicated intracellular (**B**) and extracellular (**C**) metabolites during cell cycle progression. **D** The sketch summarizes the effects of G1-HIF on the levels of the metabolites under normoxic conditions, the decay pathways for regulated amino acids are shown and green arrows show anaplerotic reactions.

The analysis of intracellular amino acids from cells grown in nutrient-rich medium showed a dynamic increase of most amino acids during progression through the cell cycle (Fig. 4C) in order to satisfy the periodic demands for the synthesis of proteins, nucleic acids and other building blocks. The absence of G1-HIF markedly reduced levels of most proteinogenic amino acids, with the exception of asparagine, cysteine, isoleucine and histidine. HIF-1α-deficiency also caused a slight increase in metabolites of the urea cycle i.e., citrulline and arginine (Fig. 4C). In nutrient-poor medium, the levels of approximately half of the intracellular amino acids also increased during the cell cycle (Fig. 4D), although the overall levels of metabolites were lower than in cells cultured in nutrient-rich conditions. The deletion of G1-HIF elicited a pronounced decrease in all of the amino acids; with the exception of isoleucine, indicating a collapse of amino acid homeostasis (Fig. 4D). Levels of glutamic acid, valine, aspartic acid, aspartate, glycine, alanine, and asparagine dropped more severely in nutrient-poor medium in the absence of G1-HIF (Fig. 5A). Other pathways were also affected in the absence of G1-HIF, as reflected by lower levels of hexose-6-phosphate, galactose-6-phosphate, malate and lactate (Fig. 5B). Extracellular lactate levels were also reduced in the absence of G1-HIF (Fig. 5C), consistent with the impaired acidification of the cell culture medium under these conditions (Fig. 2C). Further experiments showed no effect of G1-HIF on the level of reactive oxygen intermediates or oxygen consumption (Fig. S3A–D), suggesting that G1-HIF mainly functions by ensuring cell cycle-dependent supply of nutrients as summarized in Fig. 5D.

### G1-HIF regulates gene expression

**Fig. 6:**
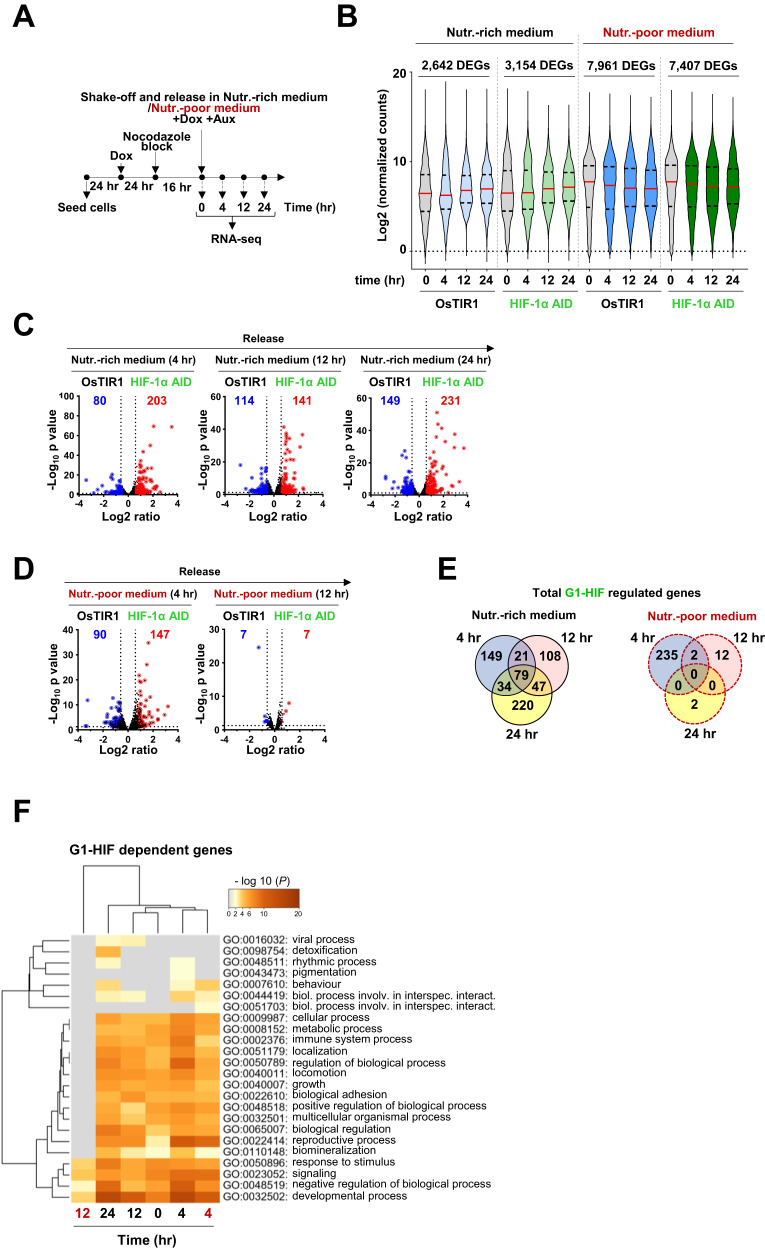
Analysis of G1-HIF- and cell cycle-dependent gene expression. **A** Scheme depicting the experimental set-up. **B** DEGs matching the filtering criteria ( ± FC ≥ 2-fold, *P* ≤ 0.05 comparing each time point to the 0 control) at 4, 12 and 24 h after cell cycle release were collected and their normalized expression values are displayed along the values in the corresponding nocodazole-arrested conditions (gray). Violin plots show the distribution of the mean expression values, red lines show medians and dashed lines indicate the interquartile range containing 50% of values above and below the median. Total numbers of DEGs according to cell type and nutritional condition are indicated. **C**, **D** Volcano plot representations of pairwise comparisons of HCT-116 OsTIR1 with HCT-116 HIF-1α AID cells across all conditions where ratio values were calculated by Desq2. Blue and red colors highlight HIF-1α AID-dependent DEGs ( ± FC ≥ 1.5-fold, *P* ≤ 0.05). **E** Venn diagram comparing the time-dependent distribution of regulated DEGs (up- or down) at nutrient-rich (left) and nutrient-poor (right) conditions. **F** G1-HIF-dependent DEGs were further used to perform an overrepresentation analysis using Metascape. The enriched pathway terms are indicated, the gray fields visualize lack of enrichment. Time points in red color refer to cells grown in nutrient-poor medium.

Because HIF-1α is a transcription factor and a fraction of G1-HIF also occurs in the nucleus (Fig. S4A), we continued to investigate a possible transcriptional function of G1-HIF during the cell cycle. HCT-116 OsTIR and control cells were synchronized and treated as shown in Fig. 6A and gene expression was analyzed 4, 12 and 24 h post release by RNA-seq. The bioinformatic analysis of two biological and two technical replicates revealed a comparable number and distribution of overall read counts (Fig. S4B–D). Principal component analyses confirmed the reproducibility of replicate experiments under the various experimental conditions and suggested that the largest variation in gene expression was related to cell cycle release (PC 1, 51,32%), followed by metabolic state (PC2, 14,98%). In nutrient-rich medium, 2642 (HCT-116 OsTIR1) and 3154 (HCT-116 HIF-1α AID) differentially expressed genes (DEGs, ±FC ≥ 2-fold, *P* ≤ 0.05) were observed upon cell cycle release, whose mean intensities significantly increased over time when compared to the expression levels in the nocodazole-arrested state (Fig. 6B, left half of the graph, and Supplemental Table S2). In nutrient-poor medium, an even larger set of DEGs ( ± FC ≥ 2-fold, *P* ≤ 0.05) was found upon cell cycle release (7961 DEGs in HCT-116 OsTIR1 and 7407 DEGs in HCT-116 HIF-1α AID). This cell stress-induced increase was lost over time, likely due to metabolic shortage and the onset of apoptosis [37, 38] (Fig. 6B, right half of the graph, and Supplemental Table S2). Overall, there was a strong correlation between all changes in gene expression when comparing OsTIR1 with HIF1α AID cells in both, the nutrition-rich and the nutrition-poor conditions, suggesting that G1-HIF only controls a limited number of genes (Fig. S4E).

To reveal the contribution of G1-HIF for gene expression at each individual time point, we performed pairwise comparisons between HCT-116 cells expressing or lacking this transcription factor (Fig. 6C, D) (±≥1.5-fold, *P* ≤ 0.05). After release in nutrient-rich medium, 283 (4 h), 255 (12 h) and 380 (24 h) genes showed G1-HIF-dependent regulation (Fig. 6C, Supplemental Table S3). Control RT-qPCR experiments confirmed the HIF-1α dependency of regulated genes (Fig. S4F). The release of arrested cells in nutrient-poor medium resulted in the detection of 237 G1-HIF-dependent genes 4 h after release, a number that dropped markedly at later time points, most likely by a lack of metabolic building blocks and the onset of cell death (Fig. 6D).

In nutrient-rich medium approximately half of the G1-HIF dependent DEGs showed HIF-1α-dependent expression at more than one time point (Fig. 6E). This distribution was not seen in nutrient-poor medium where gene expression was only intact 4 h after release within the G1 phase, but not at later time points. Mapping G1-HIF-dependent genes to gene ontology (GO) pathways using Metascape revealed the regulation of genes participating in cell proliferation, metabolism and the stress response, amongst others (Fig. 6F). G1-HIF had no significant effect on genes regulating cell death or survival, suggesting that cell death is triggered by a well-known and transcription-independent program induced solely by nutrient deficiency [37, 39, 40].

### G1-HIF-mediated regulation of metabolism employs transcriptional and non-transcriptional events

**Fig. 7:**
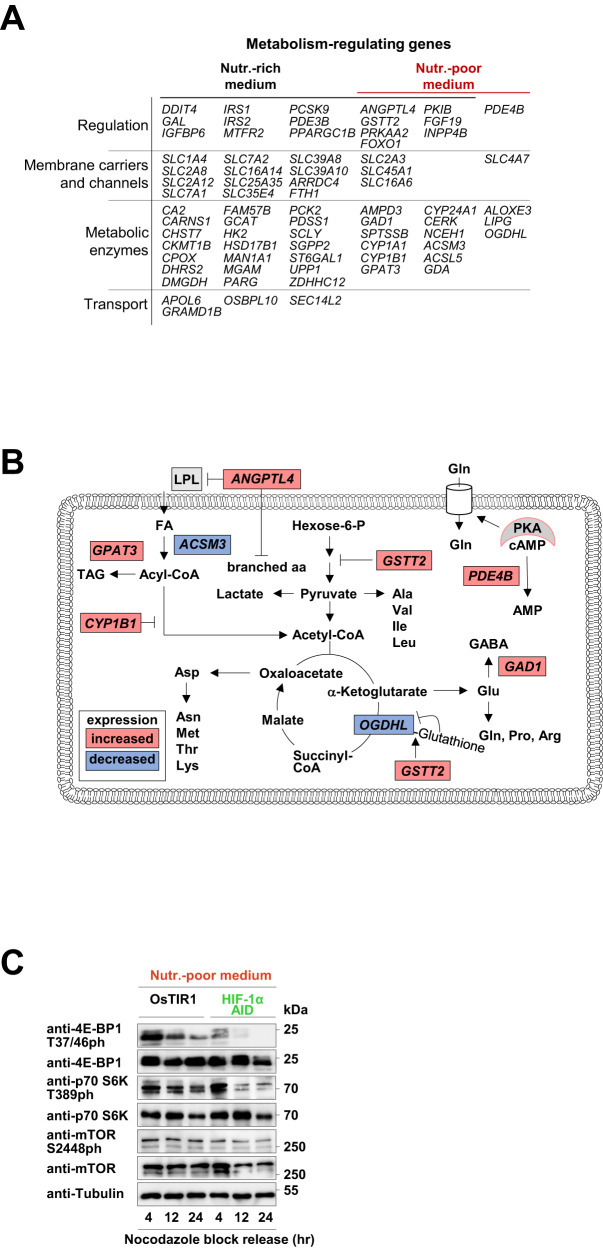
G1-HIF-mediated regulation of transcription and of signaling pathways. **A** A non-redundant list of G1-HIF-dependent DEGs ( ± FC ≥ 1.5-fold, *P* ≤ 0.05) was analyzed for a possible role in metabolic control. The indicated transcripts have reported functions at the indicated levels of metabolic regulation. **B** Transcripts displayed in (**A**) showing G1-HIF-dependent regulation in nutrient poor medium were analyzed for their possible role in metabolic regulation of the indicated metabolic pathways. The hypothetical model shows effects on FA oxidation, the metabolism of carbohydrates, the TCA cycle and amino acids. **C** HCT-116 OsTIR1 and HCT-116 HIF-1α AID cells were treated for 12 h with Dox (1 µg/mL), arrested in M phase using nocodazole and released in nutrient-poor medium containing Dox and Aux for the indicated periods. Cell extracts were analyzed by Western blotting for the expression and phosphorylation of mTOR and its indicated substrates.

A further analysis of G1-HIF-dependent genes revealed a number of transcripts with a potential role in steering of metabolic processes at different regulatory levels (Fig. 7A). Since the functional role of G1-HIF is specifically relevant in nutrient-poor medium, the genes regulated under these conditions were assigned to a map of metabolic pathways. This analysis revealed a possible function of a number of genes on different metabolic processes (Fig. 7B). The uptake and oxidation of fatty acids (FA) to acetyl-CoA can be negatively affected by several G1-HIF dependent genes. These include increased expression of *ANGPTL4* (Angiopoietin Like 4), which inhibits the catalytic activity of lipoprotein lipase (LPL) und fatty acid (FA) uptake [41] and also diminished expression of *ACSM3* (Acyl-CoA Synthetase Medium Chain Family Member 3), which contributes to the generation of Acyl-CoA. While increased expression of *CYP1B1* (Cytochrome P450 Family 1 Subfamily B Member 1) will reduce ß-oxidation [42], higher levels of *GPAT3* (Glycerol-3-Phosphate Acyltransferase 3) can limit the amounts of available Acyl-CoA by promoting its conversion to triaclyglyceride (TAG) [43]. The glycolytic flux can be downregulated by elevated expression of GSTT2 (Glutathione S-Transferase Theta 2), as this enzyme increases the glutathionylation status of metabolic enzymes which in turn can cause a global decrease of glycolysis [44]. Glutathionylation is also known to lower the activity of the TCA cycle component 2-Oxoglutarate dehydrogenase (OGDH) [45]. As also the expression of the OGDH component *OGDHL* is reduced in the absence of G1-HIF, a lower flux through the TCA cycle can be anticipated, which in turn has important implications on the synthesis and metabolism of amino acids. The level of amino acids can be further influenced by additional mechanisms, as elevated expression of *ANGPTL4* leads to impaired uptake of branched amino acids [46], while an increase in *GAD1* (Glutamate Decarboxylase 1) may result in decreased levels of its substrate glutamate. Further and rather indirect effects on amino acid levels may derive from the activity of *PDE4B* (Phosphodiesterase 4B) which limits cAMP levels and thus PKA-mediated upregulation of transporters for glutamine and other amino acids [47].

Metabolic adaptation does not rely solely on altered gene expression, but can be also controlled by signaling pathways that can interconnect and coordinate different metabolic pathways, e.g., the mTOR kinase system [48]. A possible contribution of G1-HIF to mTOR signaling was assessed by determining the phosphorylation of known substrates for the mTORC1 and mTORC2 complexes in nutrient-rich versus nutrient-poor media. While no effects were apparent in nutrient-rich medium (Fig. S5), the absence of G1-HIF markedly decreased levels of mTOR activity in nutrient-poor medium. This resulted in an attenuated phosphorylation of the mTORC2 substrate AKT Ser473 (data not shown) and the mTORC1 targets 4E-BP1 (on Thr37/46) and 70S6K (on Thr389) (Fig. 7C). These experiments also revealed a slight but significant decrease in mTOR protein levels in the absence of G1-HIF. Together, these data show that G1-HIF directly or indirectly controls the expression of metabolic regulator genes and the activity of the mTOR complex.

### Accumulation of G1-HIF occurs via an AMPK-regulated pathway

**Fig. 8:**
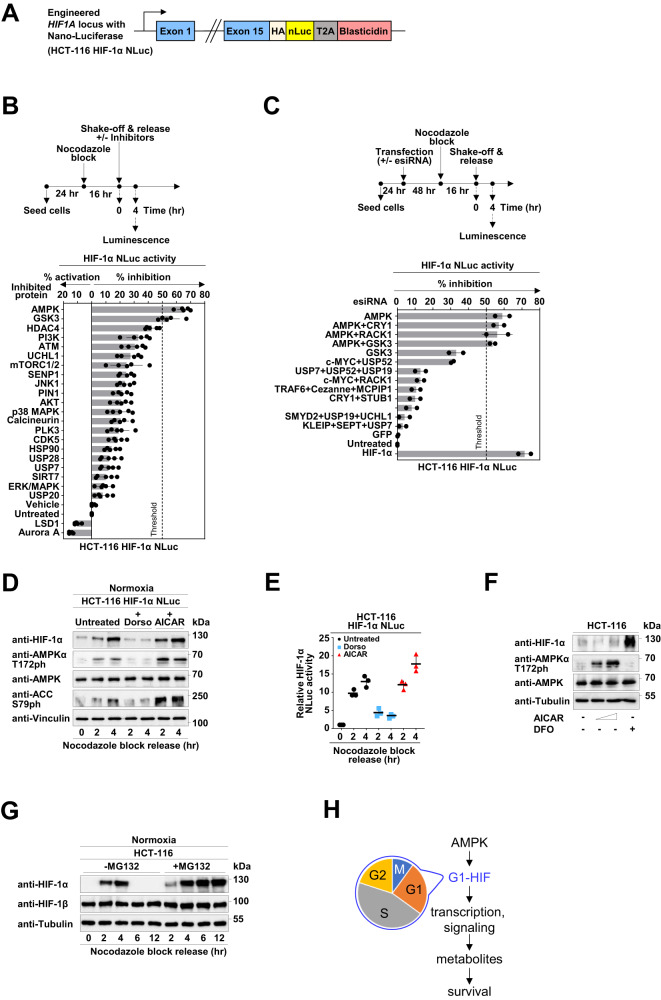
A cell cycle-regulated AMPK activation pathway contributes to G1-HIF formation. **A** Schematic display visualizing the insertion of NanoLuc luciferase into exon 15 of the *HIF1A* locus. **B** Upper: HCT-116 HIF-1α NLuc cells were synchronized via the nocodazole block/release protocol and released in the absence and presence of appropriate non-toxic concentrations of inhibitors for the indicated candidate proteins. After 4 h, Nano-Glo Luciferase Assay reagent was directly added to the cells, followed by measurement of luminescence signal on a GloMax microplate reader. Lower: The mean ± SD of five independent biological replicates is shown. A minimum of 50% inhibition in the HIF-1α NLuc signal was considered as the threshold (indicated by a dotted line). **C** HCT-116 HIF-1α NLuc cells were transfected with either one or several esiRNAs including positive (HIF-1α) and negative (GFP) controls as shown. Two days later cells were synchronized via the nocodazole block/release protocol and after 4 h the Nano-Glo luciferase assay reagent was directly added to the cells, followed by measurement of Luminescence signal. The means ± SD from two independent biological replicates performed in technical duplicates are shown. **D** HCT-116 HIF-1α NLuc cells were synchronized via nocodazole block and released in the absence and presence of Dorsomorphin (Dorso) (10 µM) or AICAR (200 µM). At the indicated time points, cells were harvested and analyzed by Western blotting. **E** Cells were treated as in (**D**), followed by measurement of luminescence after the indicated time points. Data are shown as mean ± SD and analyzed with two-way ANOVA analysis with Bonferroni multiple comparisons test (* = *P* ≤ 0.05, ** = *P* ≤ 0.01, *n* = 3). **F** HCT-116 cells were treated for 16 h with AICAR (100 and 200 µM) or DFO (10 µM) and analyzed by immunoblotting as shown. **G** HCT-116 WT cells were synchronized via nocodazole block and released. MG132 (10 µM) was added 1.5 h after the release to ensure completion of cyclin B degradation and successful mitotic exit. Cells were collected at various time points and analyzed by Western blotting using the indicated antibodies. **H** Schematic model illustrating the contribution of G1-HIF and its regulation of metabolite supply to cell survival.

RNA sequencing failed to identify changes in the expression of other HIF family members or proteins reported to regulate HIF-1α stability (Fig. S6A). There were also no changes in protein levels of HIF-1α or its hydroxylation and ubiquitination (Fig. S6B, C). To identify factors regulating G1-HIF, an HCT-116 cell line expressing the endogenous HIF-1α protein in fusion with the NanoLuc luciferase (NLuc) peptide was generated, as schematically shown in Fig. 8A. These cells (designated as HCT-116 HIF-1α NLuc) recapitulated the cell cycle-dependent transient stabilization of the HIF-1α NLuc fusion protein during G1, as revealed by Western blotting (Fig. S7A) and determination of luciferase activity (Fig. S7B).

To screen for proteins with a potential relevance for the oxygen-independent pathway leading to the generation of G1-HIF, potential candidate regulators were retrieved from the literature (Supplemental Table S4). In the first step, we investigated the influence of commercially available inhibitors on the transient increase of NLuc activity during G1, as schematically displayed in Fig. 8B. Only interference with AMPK and glycogen synthase kinase 3 (GSK3) resulted in more than 50% inhibition of HIF-1α expression. To screen for further G1-HIF activating candidate proteins not amenable to small molecule inhibitors, a siRNA-mediated gene silencing approach was used. HCT-116 HIF-1α NLuc cells were transfected with highly effective endoribonuclease prepared siRNAs (esiRNAs) to reduce the expression of either one or several candidate proteins. After 2 days, cells were synchronized by nocodazole treatment and release. Quantification of the NLuc signal 4 h after release showed that only the esiRNA leading to the downregulation of AMPKα1 resulted in significant reduction of NLuc activity (Fig. 8C, Fig. S7C). As both independent screening approaches suggested the relevance of AMPK for the generation of G1-HIF, it was interesting to investigate the activation of this kinase during the cell cycle and to reveal the contribution of its kinase activity for G1-HIF occurrence. To address this question, nocodazole-arrested HCT-116 HIF-1α NLuc cells were released into the G1 phase for 2 and 4 h in the presence of the AMPK inhibitor Dorsomorphin (compound C) [49] or the AMPK activating compound AICAR [50]. These experiments showed a transient activation of AMPK during the G1 phase, as seen by the increased phosphorylation of threonine 172 (pAMPKα Thr 172) in its catalytic subunit and phosphorylation of its well-known substrate protein acetyl-coenzyme A carboxylase (ACC) (Fig. 8D), which was seen in several cell types (Fig. S7D). At the protein level the induction of G1-HIF was strongly inhibited by Dorsomorphin and slightly increased by AICAR (Fig. 8D), but these changes did not occur at the *HIF1A* mRNA levels (Fig. S76E). The inhibitory effect of Dorsomorphin and the slightly stimulating effect of AICAR were fully recapitulated at the level of NLuc activity (Fig. 8E). Further experiments showed that AICAR alone cannot trigger the stabilization of HIF-1α (Fig. 8F), revealing that AMPK is necessary, but not sufficient to mediate the generation of G1-HIF. While induction of G1-HIF is mediated by an AMPK-driven and oxygen-independent pathway, degradation of this transcription factor did not occur in the presence of the proteasome inhibitor MG132 (Fig. 8G) and thus proceeds by the ubiquitin/proteasome system.

## Discussion

This study reveals stabilization of the endogenous HIF-1α protein during the G1 phase of the cell cycle in normoxia. We hypothesize that this transiently generated G1-HIF serves as a cellular surveillance factor to ensure the availability of nutrients before entering into the metabolically demanding process of DNA synthesis and cell mass duplication. Similar cell cycle-dependent surveillance mechanisms include the spindle assembly checkpoint and the mitotic surveillance pathway [51]. These fail-safe control points have in common that they become functionally relevant only upon the occurrence of problems such as centrosome loss or nutritional stress. The induction of G1-HIF timely coincides with the restriction point, which is functionally defined as the point-of-no-return to complete DNA synthesis [52] and is biochemically seen by phosphorylation of the retinoblastoma protein [53]. While surveillance by G1-HIF does not play a discernible role in the presence of high nutrient levels, it becomes functionally relevant during metabolic stress where it prevents cell death. In these situations, G1-HIF is required to provide sufficient amounts of amino acids and other metabolites to enable cell survival. Furthermore, inactivation of the pro-survival mTOR pathway in the absence of G1-HIF will likely contribute to cell death [54]. As schematically visualized in Fig. 8H, G1-HIF-dependent maintenance of metabolic homeostasis shifts the threshold delineating cell survival and a well-known cell death program initiated by nutrient shortage [37–39]. We can formally not rule out that the G1-HIF-dependent massive drop of nutients in nutrient-poor medium might be a consequence of cell death, although we did not see any G1-HIF-dependent changes in the expression of apoptosis-regulating genes [55].

G1-HIF affects the expression of genes whose products can influence various distinct metabolic processes (Fig. 7A, B). Accordingly, G1-HIF protected against cell death induced by a deficiency of glutamine or an absence of pyruvate/glucose. Although we do not know the relative contribution of individual G1-HIF-regulated genes on the various metabolic changes, it is reasonable to assume that the totality of the measured gene expression changes will impact biochemical pathways and affect nutrient availability. In addition, it is possible that G1-HIF can induce non-transcriptional effects, as they were described for hypoxia-regulated HIF-1α [27, 56]. It is currently not clear whether G1-HIF acts directly on the mTOR signaling pathway or whether the observed downregulation of mTORC1 and mTORC2 activity in the absence of G1-HIF in nutrient-poor medium results from nutrient shortage occurring under these conditions.

This study did not reveal a role of G1-HIF for cell division, and consistently a number of studies showed that tissue-specific deletion of the *Hif1a* gene in mice did not cause any major effects on proliferation and rather affected other processes such as stem cell maintenance and development [57–59]. However, this study does not exclude a possible function of G1-HIF for cell cycle progression, as the role of oxygen-independent HIF-1α for this process depends on the cell type and the (patho)physiological situation [60]. Previous studies reported that the M phase kinase CDK1 can increase the stability of HIF-1α under hypoxic conditions [61, 62]. We never observed any stabilization of HIF-1α in mitotic cells of various origins, suggesting that the stabilizing function of the CDK1/cyclin B complex is confined to conditions of low oxygen availability.

The increase of HIF-1α protein during the G1 phase involves an AMPK-dependent mechanism, as revealed by two independent loss-of-function screens. Activation of AMPK is necessary, but not sufficient for the generation of G1-HIF. The additional signal(s) leading to the oxygen-independent generation of G1-HIF are currently not known. While our data exclude significant transcriptional induction of the genes encoding *HIF1A* or its regulators, a further mechanism could employ increased synthesis of the HIF-1α protein at the ribosome [63]. The transient increase of AMPKα1 activity during the G1 phase has also been observed in mouse fibroblasts, where this process is dependent on Ca^2+^ transients and CaMKKß activity [64]. We observed that the activation of AMPK occurs independent from G1-HIF (Fig. S8A) and is unlikely to occur by changes in the ATP/AMP ratios which slightly increase during G1 [6, 65]. As also Ca^2+^ concentrations were largely constant during the cell cycle in HCT-116 cells (Fig. S8B), it can be assumed that transient induction of AMPK activity may be attributed to the induction of further signaling events and/or to G1-specific changes in the expression of several known AMPK regulators, as displayed in Fig. S8C.

Although the function of AMPK for the oxygen-independent activation of HIF-1α does not reveal a coherent picture [13], a HIF-1α stabilizing function of AMPK was suggested by several studies. Genetic deletion of AMPKα2 in myeloid cells prevents HIF-1α stabilization in neutrophils [66] and furthermore AMPK was found to decrease HIF ubiquitination while triggering its transcriptional activity [67]. A further mechanism could involve direct AMPK-mediated phosphorylation of HIF-1α at S419, which has been detected by in vitro kinase assays in *C. elegans* [68]. It remains to be studied whether this phosphorylation also occurs for human G1-HIF and whether it is functionally relevant.

It should not go unmentioned that the HIF transcription factor is functionally associated with another fundamental periodic process, namely the circadian cycle. The circadian cycle and the cell cycle occur with a period in the range of 1 day and are highly connected [69]. It is interesting to note that the HIF transcription factor system shows mutual cross-regulation with various components of circadian clock proteins [70, 71] including AMPK, which also regulates circadian rhythms in an isoform and -tissue-specific manner [72]. We assume that these rhythmic processes contribute to a part of the constitutive HIF functions occurring under normoxic conditions.

## Limitations of the study

While we used a number of transformed and non-transformed human cell lines to substantiate the evidence the expression of G1-HIF is not limited to a particular cell system, we have not investigated G1-HIF in an intact organism. At the organism level, only small subpopulations of cells are likely to have a sufficiently high proliferation rate to reliably detect G1-HIF. The pronounced conditions of metabolic stress used here mimic situations of prolonged fasting leading to autophagy or conditions that occur in tissues with strongly fluctuating energy requirements such as muscles or in poorly vascularized solid tumors [73].

## Materials and methods

### Cell culture, transfection and synchronization

HCT-116 (ATCC: CCL-247™) cells and their derivatives, HeLa (ATCC: CRM-CCL-2) and U2OS (ATCC: HTB-96) cells were cultured in DMEM supplemented with 10% (v/v) FCS, penicillin (100 U/ml) and streptomycin (100 μg/ml) at 37 °C. Primary human FS4-LTM fibroblasts (inSCREENex, Braunschweig, Germany) were grown in huFIB Medium (InSCREENeX GmbH, Braunschweig, Germany) and their proliferation was induced by addition of doxycycline (1 µg/mL) [74]. All cells were regularly tested for potential contamination by mycoplasma and grown on cell culture dishes in a humidified atmosphere containing 5% (v/v) CO_2_. Transfection was done using the Lipofectamine® 3000 transfection protocol (ThermoFisher). For stable genomic integration of DNA, HCT-116 cells were transfected with a plasmid directing the expression of Cas9 and an appropriate sgRNA for targeting of the *HIF1A* locus along with two further plasmids: One plasmid encoding the inserted sequence flanked by two homology arms with a length of 0.5 kb and containing short PITCh sequences at their distal ends. A second plasmid directing the expression of Cas9 and an appropriate sgRNA cleaving the PITCh sequences, thus creating a DNA fragment [75]. Two days after transfection, Blasticidin (7 µg/ml) was added for 10 days to enable proliferation of cells with a genomic insertion of the resistance gene. Cell colonies were picked and further characterized by PCR analysis of the genomic locus and Western blotting. All further experiments were conducted with pools of several cell clones to avoid clonal effects. Knockdown was done using MISSION esiRNA (Sigma) using the X-tremeGENE siRNA transfection reagent according to the recommendations of the manufacturer. Mitotic synchronization was performed by addition of nocodazole (0.1 µg/ml) for 16 h. Mitotic cells were harvested by mitotic shake-off, washed three times with warm phosphate-buffered saline (PBS). Equal numbers of cells were released into fresh medium. G1/S synchronization was done by addition of thymidine (2 mM) for 16 h. Cells were then washed three times with warm PBS and released into normal DMEM medium for 8 h. Thymidine (2 mM) was added again for 16 h, followed by three washes with warm PBS and addition of fresh medium.

### Screening experiments

Small molecule inhibitor screening: A pool of cell clones showing proper insertion of NanoLuc luciferase into exon 15 of the *HIF1A* locus (HCT-116 HIF-1α NLuc cells) was synchronized via the nocodazole block/release protocol and released in the absence and presence of appropriate concentrations of inhibitors (Supplemental table S4). After 4 h, one Volume of the Nano-Glo Luciferase Assay reagent (freshly prepared by mixing Nano-Glo luciferase assay substrate with Nano-Glo luciferase assay buffer (1:50)) was added to the cells and luciferase activity was recorded using a GloMax® Discover microplate reader (Promega). All experiments were performed in five independent experiments. For screening after knockdown via esiRNAs, two independent experiments in technical duplicates were performed. HCT-116 HIF-1α NLuc cells were transfected with 20 ng of esiRNA (either one or group of esiRNAs) using the X-tremeGENE siRNA transfection reagent (Transfection Reagent: esiRNA ratio of 5:1) followed by incubation for 30 min at room temperature (RT). After that, the transfection mix was carefully added to the cells in a dropwise manner. After 2 days cells were synchronized via nocodazole block and equal numbers of cells were released on a 96-well plate After 4 h, the Nano-Glo luciferase assay reagent (Promega) was directly added to the cells and luminescence was recorded as described above. Further experiments with inhibitors and siRNAs for the candidate proteins AMPK and GSK3 were performed. In these experiments, the luciferase activity was measured also between 4 and 8 h post release, in order to ensure that treatment leads to a reduction rather than to a delay of G1-HIF (data not shown).

### RNA-seq analysis

Cells were treated as described and total RNA was isolated with the NucleoSpin RNA Kit (Macherey-Nagel). Experiments were done in biological replicates, resulting in 24 RNA-seq data sets. After quality control of RNA, 1 μg RNA/sample was used as input material for the RNA sample preparations. Preparation of the RNA library and RNA-sequencing was conducted by Novogene Co., LTD (Chaoyang, Beijing, China). Raw reads were aligned to an index based on the human genome version GRCh38 using HSIAT2 v2.0.5 software [76]. Read counts were generated and Log2 transformed using FeatureCounts v1.5.0-p3 [77] from the R subread package [78]. Normalization and detection of differentially expressed genes was done using DESeq2 v1.20.0 [79] by Novogene according to their bioinformatics pipeline. The resulting data tables were filtered in Excel 2016 for DEGs using cut-offs as described in the figure legends. Venn diagrams were created with tools provided at http://bioinformatics.psb.ugent.be/webtools/Venn/. Overrepresentation analyses of differentially expressed gene sets were done using Metascape software with the express settings. Statistical tests for pathway enrichment analyses were calculated online by Metascape software (https://metascape.org/) using the ontology sources KEGG Pathway, GO Biological Processes, Reactome Gene Sets, Canonical Pathways, CORUM, TRRUST, DisGeNET, PaGenBase, Transcription Factor Targets, WikiPathways, PANTHER Pathway, and COVID and all genes in the genome as the enrichment background. *P* values were based on the accumulative hypergeometric distribution and *q* values were calculated using the Benjamini-Hochberg procedure to account for multiple testings. Terms with a *P* value < 0.01, a minimum count of 3, and an enrichment factor >1.5 were collected. Kappa scores were used as the similarity metric for hierarchical clustering on the enriched terms, and sub-trees with a similarity of >0.3 were considered a cluster. The most statistically significant terms within a cluster were chosen to represent the cluster. Quantification of data and statistical parameters (means, *t*-tests, ANOVA, standard variations, confidence intervals, Pearson correlations, linear regressions) of the RNA-seq data were calculated using DESeq2 (see above), GraphPad Prism 9.3.1, or Microsoft Excel 2016.

### Plasmids and antibodies

This information is listed in Supplemental table S5.

## Supplementary information

Supplementary Figures

suppl. Table 1

suppl. Table 2

suppl. Table 3

suppl. Table 4

suppl. Table 5

uncropped blots

## Data Availability

The experimental data sets generated and/or analyzed during the current study are available from the corresponding author upon reasonable request. No applicable resources were generated during the current study.

## References

[1] Vander Heiden MG, Cantley LC, Thompson CB (2009). Understanding the Warburg Effect: The Metabolic Requirements of Cell Proliferation. Sci (N. Y, NY).

[2] Moncada S, Higgs EA, Colombo Sergio L (2012). Fulfilling the metabolic requirements for cell proliferation. Biochem J.

[3] Colombo SL, Palacios-Callender M, Frakich N, Carcamo S, Kovacs I, Tudzarova S (2011). Molecular basis for the differential use of glucose and glutamine in cell proliferation as revealed by synchronized HeLa cells. Proc Natl Acad Sci.

[4] Agathocleous M, Harris WA (2013). Metabolism in physiological cell proliferation and differentiation. Trends Cell Biol.

[5] Ewald JC, Kuehne A, Zamboni N, Skotheim JM (2016). The Yeast Cyclin-Dependent Kinase Routes Carbon Fluxes to Fuel Cell Cycle Progression. Mol Cell.

[6] Mitra K, Wunder C, Roysam B, Lin G, Lippincott-Schwartz J (2009). A hyperfused mitochondrial state achieved at G1–S regulates cyclin E buildup and entry into S phase. Proc Natl Acad Sci.

[7] Lopez-Mejia IC, Lagarrigue S, Giralt A, Martinez-Carreres L, Zanou N, Denechaud P-D (2017). CDK4 Phosphorylates AMPKα2 to Inhibit Its Activity and Repress Fatty Acid Oxidation. Mol Cell.

[8] Wang H, Nicolay BN, Chick JM, Gao X, Geng Y, Ren H (2017). The metabolic function of cyclin D3-CDK6 kinase in cancer cell survival. Nature.

[9] Lee Y, Dominy JE, Choi YJ, Jurczak M, Tolliday N, Camporez JP (2014). Cyclin D1-Cdk4 controls glucose metabolism independently of cell cycle progression. Nature.

[10] Schwarz C, Johnson A, Kõivomägi M, Zatulovskiy E, Kravitz CJ, Doncic A (2018). A Precise Cdk Activity Threshold Determines Passage through the Restriction Point. Mol Cell.

[11] Talarek N, Gueydon E, Schwob E (2017). Homeostatic control of START through negative feedback between Cln3-Cdk1 and Rim15/Greatwall kinase in budding yeast. Elife.

[12] Inoki K, Kim J, Guan KL (2012). AMPK and mTOR in cellular energy homeostasis and drug targets. Annu Rev Pharm Toxicol.

[13] Dengler F (2020). Activation of AMPK under Hypoxia: Many Roads Leading to Rome. Int J Mol Sci.

[14] Wang GL, Jiang BH, Rue EA, Semenza GL (1995). Hypoxia-inducible factor 1 is a basic-helix-loop-helix-PAS heterodimer regulated by cellular O2 tension. Proc Natl Acad Sci USA.

[15] Jaakkola P, Mole DR, Tian YM, Wilson MI, Gielbert J, Gaskell SJ (2001). Targeting of HIF-alpha to the von Hippel-Lindau ubiquitylation complex by O2-regulated prolyl hydroxylation. Science.

[16] Lando D, Peet DJ, Whelan DA, Gorman JJ, Whitelaw ML (2002). Asparagine hydroxylation of the HIF transactivation domain a hypoxic switch. Science.

[17] Semenza GL (2012). Hypoxia-inducible factors in physiology and medicine. Cell.

[18] Iommarini L, Porcelli AM, Gasparre G, Kurelac I (2017). Non-Canonical Mechanisms Regulating Hypoxia-Inducible Factor 1 Alpha in Cancer. Front Oncol.

[19] Lee BL, Kim WH, Jung J, Cho SJ, Park JW, Kim J (2008). A hypoxia-independent up-regulation of hypoxia-inducible factor-1 by AKT contributes to angiogenesis in human gastric cancer. Carcinogenesis.

[20] Chun SY, Johnson C, Washburn JG, Cruz-Correa MR, Dang DT, Dang LH (2010). Oncogenic KRAS modulates mitochondrial metabolism in human colon cancer cells by inducing HIF-1alpha and HIF-2alpha target genes. Mol Cancer.

[21] Kietzmann T, Mennerich D, Dimova EY (2016). Hypoxia-Inducible Factors (HIFs) and Phosphorylation: Impact on Stability, Localization, and Transactivity. Front Cell Dev Biol.

[22] Liu YV, Baek JH, Zhang H, Diez R, Cole RN, Semenza GL (2007). RACK1 competes with HSP90 for binding to HIF-1alpha and is required for O(2)-independent and HSP90 inhibitor-induced degradation of HIF-1alpha. Mol Cell.

[23] Schober AS, Berra E (2016). DUBs, New Members in the Hypoxia Signaling clUb. Front Oncol.

[24] Sun H, Li X-B, Meng Y, Fan L, Li M, Fang J (2013). TRAF6 upregulates expression of HIF-1α and promotes tumor angiogenesis. Cancer Res.

[25] Schödel J, Oikonomopoulos S, Ragoussis J, Pugh CW, Ratcliffe PJ, Mole DR (2011). High-resolution genome-wide mapping of HIF-binding sites by ChIP-seq. Blood.

[26] Smythies JA, Sun M, Masson N, Salama R, Simpson PD, Murray E (2019). Inherent DNA-binding specificities of the HIF-1alpha and HIF-2alpha transcription factors in chromatin. EMBO Rep..

[27] Hubbi ME, Luo W, Baek JH, Semenza GL (2011). MCM proteins are negative regulators of hypoxia-inducible factor 1. Mol Cell.

[28] Hubbi ME, Kshitiz N, Gilkes DM, Rey S, Wong CC, Luo W (2013). A nontranscriptional role for HIF-1α as a direct inhibitor of DNA replication. Sci Signal.

[29] Li H-S, Zhou Y-N, Li L, Li S-F, Long D, Chen X-L (2019). HIF-1α protects against oxidative stress by directly targeting mitochondria. Redox Biol.

[30] Villa JC, Chiu D, Brandes AH, Escorcia FE, Villa CH, Maguire WF (2014). Non-transcriptional role of Hif-1α in activation of γ-secretase and Notch signaling in breast cancer. Cell Rep..

[31] Schlereth K, Beinoraviciute-Kellner R, Zeitlinger MK, Bretz AC, Sauer M, Charles JP (2010). DNA binding cooperativity of p53 modulates the decision between cell-cycle arrest and apoptosis. Mol Cell.

[32] Loewer A, Batchelor E, Gaglia G, Lahav G (2010). Basal dynamics of p53 reveal transcriptionally attenuated pulses in cycling cells. Cell.

[33] Tudzarova S, Colombo SL, Stoeber K, Carcamo S, Williams GH, Moncada S (2011). Two ubiquitin ligases, APC/C-Cdh1 and SKP1-CUL1-F (SCF)-beta-TrCP, sequentially regulate glycolysis during the cell cycle. Proc Natl Acad Sci USA.

[34] Natsume T, Kiyomitsu T, Saga Y, Kanemaki MT (2016). Rapid Protein Depletion in Human Cells by Auxin-Inducible Degron Tagging with Short Homology Donors. Cell Rep..

[35] Papandreou I, Cairns RA, Fontana L, Lim AL, Denko NC (2006). HIF-1 mediates adaptation to hypoxia by actively downregulating mitochondrial oxygen consumption. Cell Metab.

[36] Semenza GL, Roth PH, Fang HM, Wang GL (1994). Transcriptional regulation of genes encoding glycolytic enzymes by hypoxia-inducible factor 1. J Biol Chem.

[37] Yuneva M, Zamboni N, Oefner P, Sachidanandam R, Lazebnik Y (2007). Deficiency in glutamine but not glucose induces MYC-dependent apoptosis in human cells. J Cell Biol.

[38] Fu Y-M, Zhang H, Ding M, Li Y-Q, Fu X, Yu Z-X (2006). Selective amino acid restriction targets mitochondria to induce apoptosis of androgen-independent prostate cancer cells. J Cell Physiol.

[39] Le A, Lane AN, Hamaker M, Bose S, Gouw A, Barbi J (2012). Glucose-independent glutamine metabolism via TCA cycling for proliferation and survival in B cells. Cell Metab.

[40] Wise DR, Thompson CB (2010). Glutamine addiction: a new therapeutic target in cancer. Trends Biochem Sci.

[41] Kristensen KK, Leth-Espensen KZ, Kumari A, Gronnemose AL, Lund-Winther AM, Young SG (2021). GPIHBP1 and ANGPTL4 Utilize Protein Disorder to Orchestrate Order in Plasma Triglyceride Metabolism and Regulate Compartmentalization of LPL Activity. Front Cell Dev Biol.

[42] Liu X, Huang T, Li L, Tang Y, Tian Y, Wang S (2015). CYP1B1 deficiency ameliorates obesity and glucose intolerance induced by high fat diet in adult C57BL/6J mice. Am J Transl Res.

[43] Quiroga IY, Pellon-Maison M, Suchanek AL, Coleman RA, Gonzalez-Baro MR (2019). Glycerol-3-phosphate acyltransferases 3 and 4 direct glycerolipid synthesis and affect functionality in activated macrophages. Biochem J.

[44] Mailloux RJ (2020). Protein S-glutathionylation reactions as a global inhibitor of cell metabolism for the desensitization of hydrogen peroxide signals. Redox Biol.

[45] Letourneau M, Wang K, Mailloux RJ (2021). Protein S-glutathionylation decreases superoxide/hydrogen peroxide production xanthine oxidoreductase. Free Radic Biol Med.

[46] Lin S, Miao Y, Zheng X, Dong Y, Yang Q, Yang Q (2022). ANGPTL4 negatively regulates the progression of osteosarcoma by remodeling branched-chain amino acid metabolism. Cell Death Discov.

[47] Ogura M, Taniura H, Nakamichi N, Yoneda Y (2007). Upregulation of the glutamine transporter through transactivation mediated by cAMP/protein kinase A signals toward exacerbation of vulnerability to oxidative stress in rat neocortical astrocytes. J Cell Physiol.

[48] Mossmann D, Park S, Hall MN (2018). mTOR signalling and cellular metabolism are mutual determinants in cancer. Nat Rev Cancer.

[49] Zhou G, Myers R, Li Y, Chen Y, Shen X, Fenyk-Melody J (2001). Role of AMP-activated protein kinase in mechanism of metformin action. J Clin Investig.

[50] Sullivan JE, Brocklehurst KJ, Marley AE, Carey F, Carling D, Beri RK (1994). Inhibition of lipolysis and lipogenesis in isolated rat adipocytes with AICAR, a cell-permeable activator of AMP-activated protein kinase. FEBS Lett.

[51] Lambrus BG, Holland AJ (2017). A New Mode of Mitotic Surveillance. Trends Cell Biol.

[52] Pardee AB (1974). A restriction point for control of normal animal cell proliferation. Proc Natl Acad Sci USA.

[53] Pennycook BR, Barr AR (2020). Restriction point regulation at the crossroads between quiescence and cell proliferation. FEBS Lett.

[54] Yellen P, Saqcena M, Salloum D, Feng J, Preda A, Xu L (2011). High-dose rapamycin induces apoptosis in human cancer cells by dissociating mTOR complex 1 and suppressing phosphorylation of 4E-BP1. Cell Cycle.

[55] Green DR, Llambi F (2015). Cell death signaling. Cold Spring Harb Perspect Biol.

[56] Briston T, Yang J, Ashcroft M (2011). HIF-1alpha localization with mitochondria: a new role for an old favorite?. Cell Cycle.

[57] Li L, Candelario KM, Thomas K, Wang R, Wright K, Messier A (2014). Hypoxia inducible factor-1alpha (HIF-1alpha) is required for neural stem cell maintenance and vascular stability in the adult mouse SVZ. J Neurosci.

[58] Bohuslavova R, Cerychova R, Papousek F, Olejnickova V, Bartos M, Gorlach A (2019). HIF-1alpha is required for development of the sympathetic nervous system. Proc Natl Acad Sci USA.

[59] Provot S, Zinyk D, Gunes Y, Kathri R, Le Q, Kronenberg HM (2007). Hif-1alpha regulates differentiation of limb bud mesenchyme and joint development. J Cell Biol.

[60] Hubbi ME, Semenza GL (2015). Regulation of cell proliferation by hypoxia-inducible factors. Am J Physiol - Cell Physiol.

[61] Hubbi ME, Gilkes DM, Hu H, Kshitiz N, Ahmed I, Semenza GL (2014). Cyclin-dependent kinases regulate lysosomal degradation of hypoxia-inducible factor 1α to promote cell-cycle progression. Proc Natl Acad Sci USA.

[62] Warfel NA, Dolloff NG, Dicker DT, Malysz J, El-Deiry WS (2013). CDK1 stabilizes HIF-1α via direct phosphorylation of Ser668 to promote tumor growth. Cell Cycle (Georget, Tex).

[63] Koritzinsky M, Magagnin MG, van den Beucken T, Seigneuric R, Savelkouls K, Dostie J (2006). Gene expression during acute and prolonged hypoxia is regulated by distinct mechanisms of translational control. EMBO J.

[64] Park IJ, Tran QH, Amin ASM, Chu TL, Yang G, Choe W (2018). Transient activation of AMP-activated protein kinase at G1/S phase transition is required for control of S phase in NIH3T3 cells. Biochem Biophys Res Commun.

[65] Schieke SM, McCoy JP, Finkel T (2008). Coordination of mitochondrial bioenergetics with G1 phase cell cycle progression. Cell Cycle.

[66] Abdel Malik R, Zippel N, Frömel T, Heidler J, Zukunft S, Walzog B (2017). AMP-Activated Protein Kinase α2 in Neutrophils Regulates Vascular Repair via Hypoxia-Inducible Factor-1α and a Network of Proteins Affecting Metabolism and Apoptosis. Circ Res.

[67] Jung S-N, Yang WK, Kim J, Kim HS, Kim EJ, Yun H (2008). Reactive oxygen species stabilize hypoxia-inducible factor-1 alpha protein and stimulate transcriptional activity via AMP-activated protein kinase in DU145 human prostate cancer cells. Carcinogenesis.

[68] Hwang AB, Ryu E-A, Artan M, Chang H-W, Kabir MH, Nam H-J (2014). Feedback regulation via AMPK and HIF-1 mediates ROS-dependent longevity in Caenorhabditis elegans. Proc Natl Acad Sci.

[69] Bieler J, Cannavo R, Gustafson K, Gobet C, Gatfield D, Naef F (2014). Robust synchronization of coupled circadian and cell cycle oscillators in single mammalian cells. Mol Syst Biol.

[70] Peek CB, Levine DC, Cedernaes J, Taguchi A, Kobayashi Y, Tsai SJ (2017). Circadian Clock Interaction with HIF1alpha Mediates Oxygenic Metabolism and Anaerobic Glycolysis in Skeletal Muscle. Cell Metab.

[71] Chilov D, Hofer T, Bauer C, Wenger RH, Gassmann M (2001). Hypoxia affects expression of circadian genes PER1 and CLOCK in mouse brain. FASEB J.

[72] Um JH, Pendergast JS, Springer DA, Foretz M, Viollet B, Brown A (2011). AMPK regulates circadian rhythms in a tissue- and isoform-specific manner. PLOS ONE.

[73] Vander Heiden MG, DeBerardinis RJ (2017). Understanding the Intersections between Metabolism and Cancer Biology. Cell.

[74] Riedlinger T, Bartkuhn M, Zimmermann T, Hake SB, Nist A, Stiewe T (2019). Chemotherapeutic Drugs Inhibiting Topoisomerase 1 Activity Impede Cytokine-Induced and NF-kappaB p65-Regulated Gene Expression. Cancers (Basel).

[75] Sakuma T, Nakade S, Sakane Y, Suzuki K-IT, Yamamoto T (2016). MMEJ-assisted gene knock-in using TALENs and CRISPR-Cas9 with the PITCh systems. Nat Protoc.

[76] Kim D, Paggi JM, Park C, Bennett C, Salzberg SL (2019). Graph-based genome alignment and genotyping with HISAT2 and HISAT-genotype. Nat Biotechnol.

[77] Liao Y, Smyth GK, Shi W (2014). featureCounts: an efficient general purpose program for assigning sequence reads to genomic features. Bioinformatics.

[78] Liao Y, Smyth GK, Shi W (2019). The R package Rsubread is easier, faster, cheaper and better for alignment and quantification of RNA sequencing reads. Nucleic Acids Res.

[79] Love MI, Huber W, Anders S (2014). Moderated estimation of fold change and dispersion for RNA-seq data with DESeq2. Genome Biol.

