# Supplementary material for: A transient increase of HIF-1α during the G1 phase (G1-HIF) ensures cell survival under nutritional stress
Source: Cell Death Dis. 2023 Jul 27;14(7):477. doi: 10.1038/s41419-023-06012-7 (PMC10374543; doi:10.1038/s41419-023-06012-7)
Supplement: Supplementary file 1 — Supplementary Figures [file 41419_2023_6012_MOESM1_ESM.pdf]

A transient increase of HIF-1α during the G1 phase (G1-HIF) controls amino acid homeostasis to ensure cell survival under nutritional stress

Ratnal Belapurkar, Maximilian Pfisterer, Jan Dreute, Sebastian Werner, Sven Zukunft, Ingrid Fleming, Michael Kracht and M. Lienhard SCHMITZ

Supplementary Figures

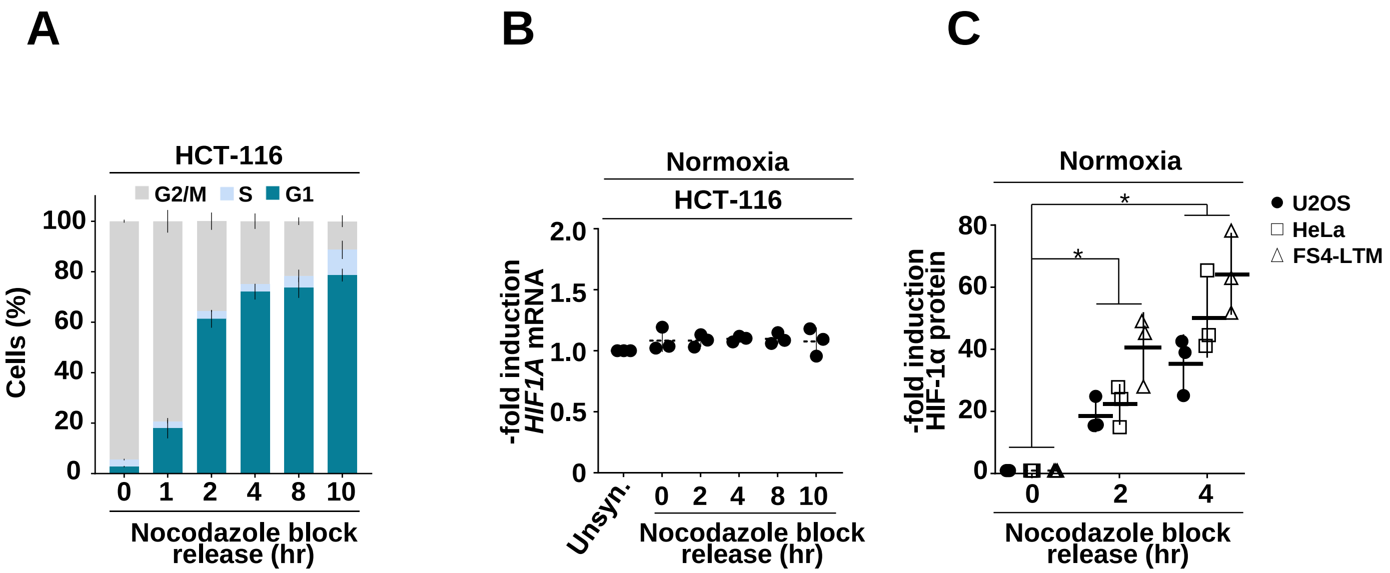

Fig. S1. Cell cycle-dependent expression of HIF-1α in normoxia. (A) HCT-116 cells were synchronized via nocodazole block/release. Cells were collected at the indicated time points and analyzed for the distribution of the cell cycle phases by flow cytometry. The mean ± SD of three independent biological replicates is shown. (B) HCT-116 cells were synchronized via the nocodazole block/release protocol and the levels of HIF-1α-encoding transcripts were determined by RT-qPCR. Transcript levels of unsynchronized cells were set to 1, the mean ± SD of three independent biological replicates is shown. (C) Different transformed (HeLa and U2OS) and primary (FS4-LTM) cells were synchronized via the nocodazole block/release protocol and cell extracts were analyzed by immunoblotting for G1-HIF. The protein amounts of G1-HIF were quantified and normalized. Data show the mean ± SD analysed with two-way ANOVA with Tukey multiple comparisons test (* = $P \leq 0.05$, n = 3).

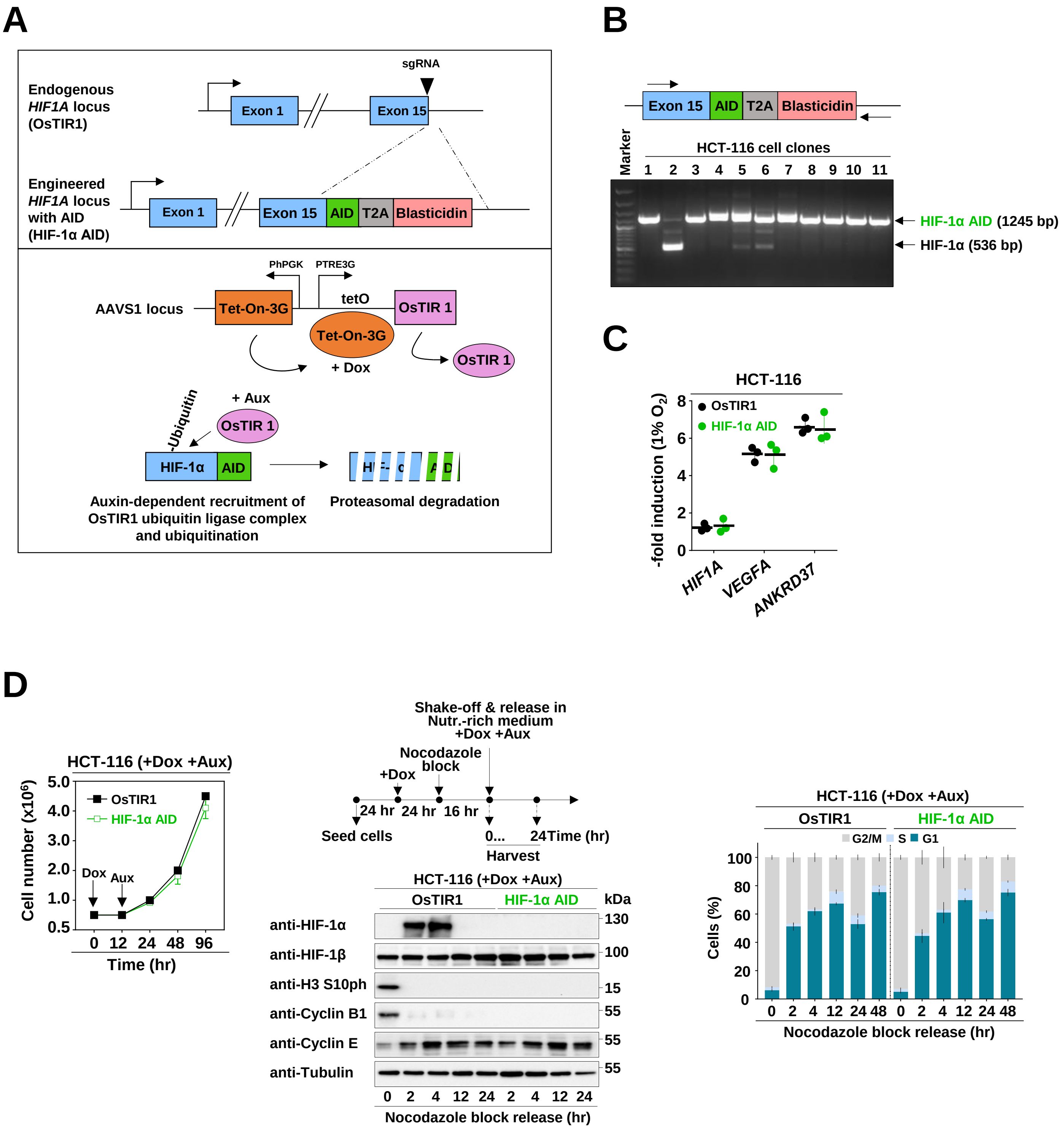

Fig. S2. G1-HIF protects from cell death induced by nutritional stress. (A) Upper: Schematic illustration of the targeting strategy for the fusion of the AID to the C-terminus of HIF-1α. Lower: Visualization of Dox/Aux-inducible elimination of the HIF-AID protein. (B) Genomic DNA was isolated, and PCR was performed using primers flanking the homology arms (indicated by arrows). Correct genomic insertion of the AID should result in a 1245 bp PCR product, whereas the untargeted locus will result in a PCR product of 536 bp, an ethidium bromide-stained agarose gel is shown. (C) To ensure that the insertion of AID cassette does not affect HIF-1α activity, the indicated cells were incubated in the presence of 1% O_2 for 4 h, followed by RNA isolation, cDNA synthesis and RT-qPCR detecting the indicated genes including the HIF target genes *VEGFA* and *ANKRD37*. The mean ± SD of three independent biological replicates is shown, gene expression observed under normoxic conditions was set to 1. (D) Left: HCT-116 OsTIR1 and HCT-116 HIF-1α AID cells were treated for 12 h with Dox (1 µg/mL), followed by addition of Aux (100 µM) as shown. The number of cells was counted every day using a LUNA automated cell counter. The mean ± SD of two independent biological replicates is shown. Middle: Cells were treated as shown and released from a nocodazole block in nutrient-rich medium. At various time points post release, cells were either used for Western blot analysis or alternatively for determination of the cell cycle stage by flow cytometry (right). The mean ± SD of three independent biological replicates is shown (..for continuation see next page).

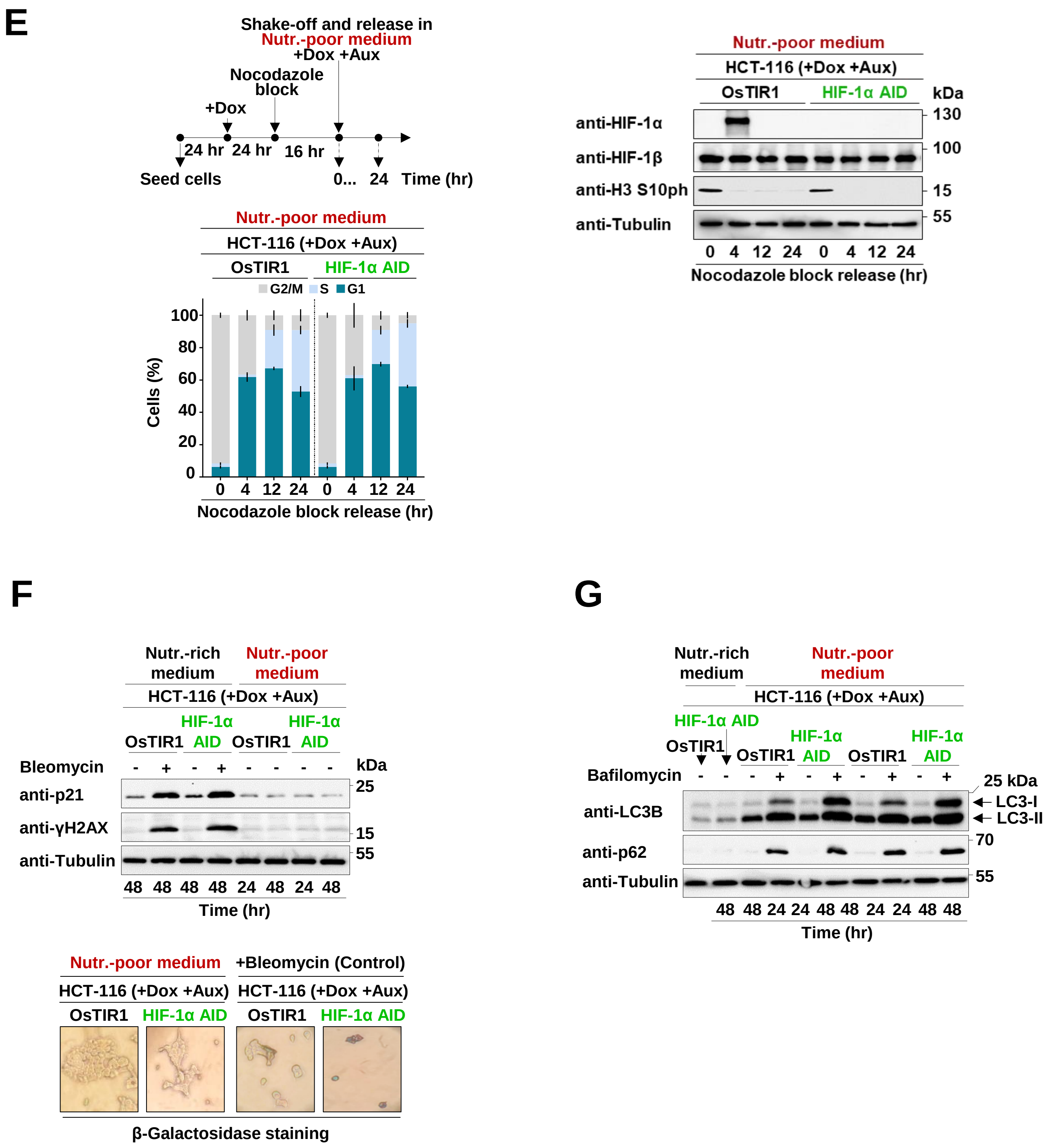

Fig. S2. Continued from previous page. G1-HIF protects from cell death induced by nutritional stress. (E) The experiment was done as in (D) with the exception that cells were released into nutrient-poor medium. (F) HCT-116 OsTIR1 (referred to as OsTIR1 in this figure) and HCT-116 HIF-1α AID (referred to as AID in this figure) cells were seeded and pre-treated with Dox, followed by further culturing in nutrient-rich medium or nutrient-poor medium in the presence of Aux +/- bleomycin (10 µg/mL) for the indicated periods, to induce senescence. Cells were either analyzed by Western blotting for the induction of senescence markers p21 and phosphorylation of H2AX (upper), or stained for the senescence marker ß-Galactosidase after 2 days of incubation, as seen by blue staining (lower). (G) Cells were incubated with the indicated media containing Dox, Aux and bafilomycin A1 (100 nM/mL) for the indicated periods to measure the autophagic flux. Cells were collected and analyzed by Western blotting for LC3 modification and decay of the autophagosome substrate p62/SQSTM1.

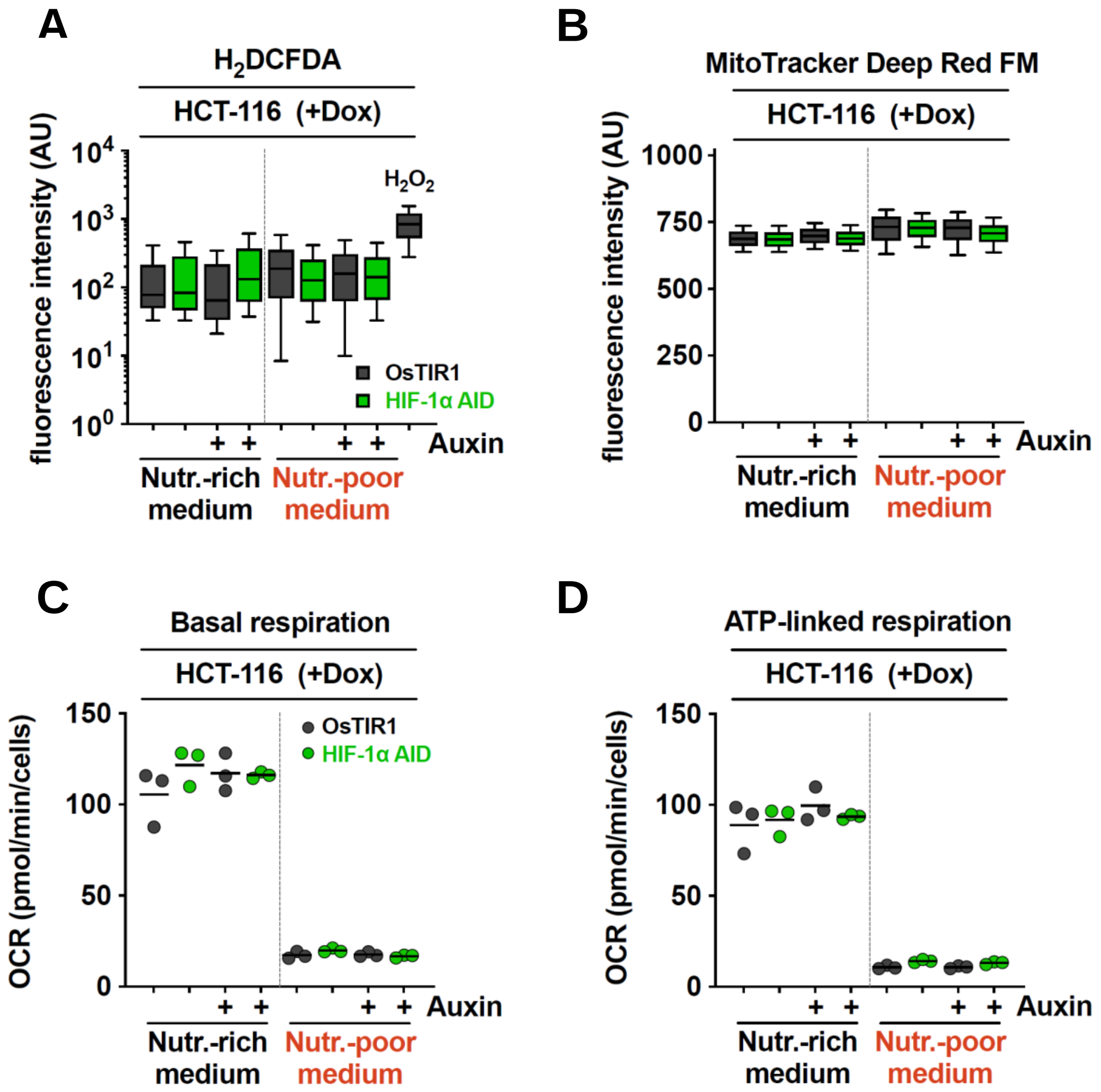

Figure S3. Analysis of G1-HIF effects on oxidative stress and oxygen consumption. HCT-116 OsTIR1 and HCT-116 HIF-1α AID cells were treated for 24 h with Dox (1 µg/mL), following 20 h of incubation in nutrient-rich or nutrient-poor medium in the presence or absence of Aux (100 µM) as shown. Cells were further analyzed for intracellular levels of reactive oxygen species (ROS) (A), mitochondrial abundance (B), basal respiration (C) and ATP-linked respiration (D). ROS levels were determined by loading of cells with the cell-permeable ROS probe H_2DCFDA (1 µM) for 20 min, followed by recording the fluorescence of the oxidized probe by flow cytometry. As a positive control of oxidative stress, cells were treated with H_2O_2 (200 µM) for 30 min, fluorescence intensity is displayed in arbitrary units (AU). For the analysis of mitochondrial abundance, cells were loaded with MitoTracker Deep Red FM (200 nM) for 15 min, followed by flow-cytometric analysis of MitoTracker fluorescence. The box plot displays the data between the first and third quartiles and the median is indicated. Whiskers extend to the 10th and 90th percentile. Oxygen consumption rate (OCR) was measured by Seahorse metabolic flux analysis. ATP-linked respiration was measured after treatment with Oligomycin (2 µM). Basal and ATP-linked respiration was calculated by subtraction of non-mitochondrial respiration after treatment with FCCP (2 µM) and Rotenone/Antimycin A (0.75 µM each). Data points from three independent experiments are displayed, the median is indicated.

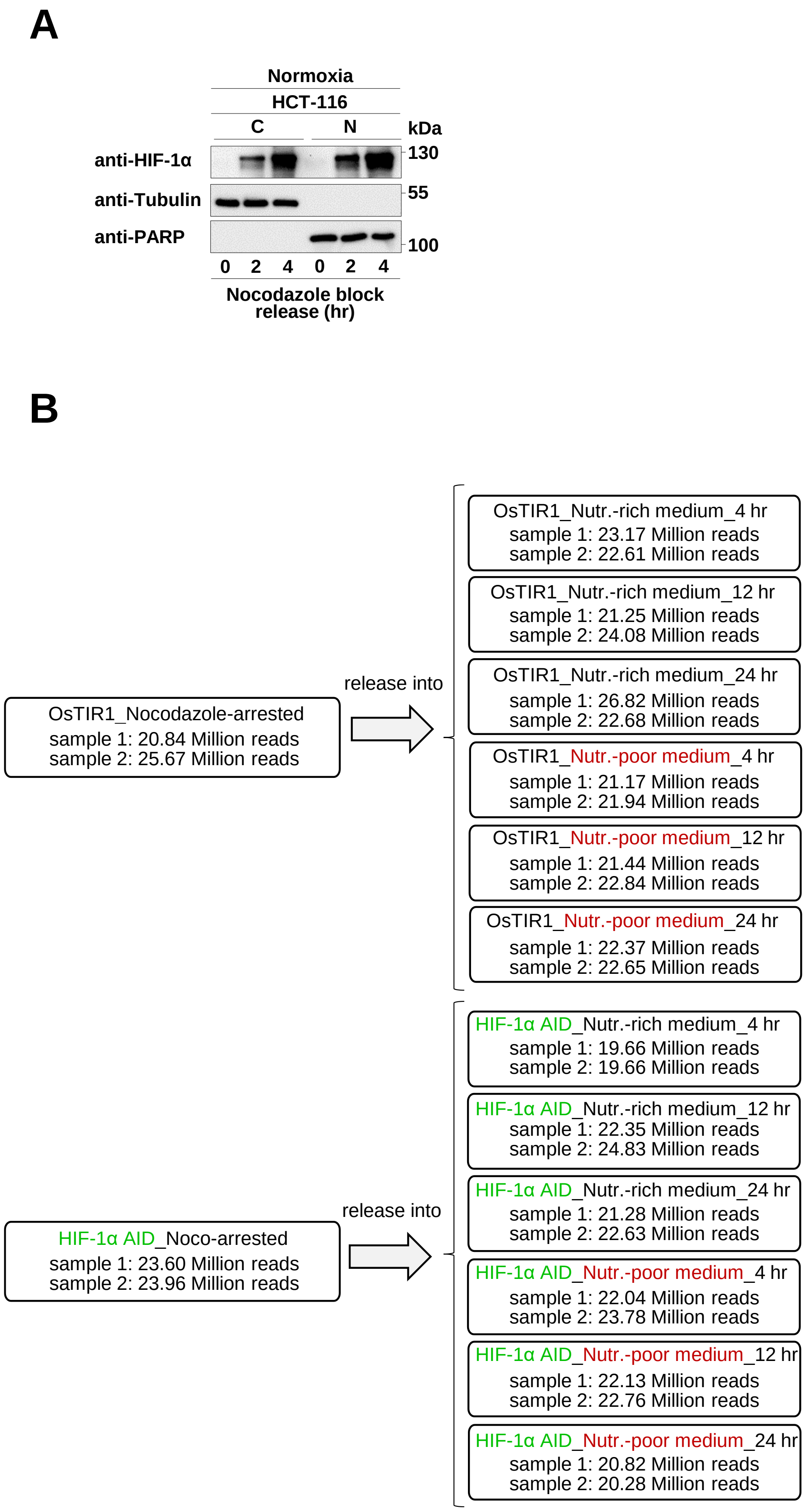

Fig. S4. Analysis of G1-HIF- and cell cycle-dependent gene expression. (A) HCT-116 cells were synchronized via the nocodazole block/release protocol, followed by fractionation into cytosolic (C) and nuclear (N) fractions. Western blotting was used to analyze the localization of G1-HIF. PARP and Tubulin staining was used to determine the purity of both fractions. (B) Cells were treated as shown in Fig. 6A and subjected to RNA-seq. The number of reads per condition are indicated (..for continuation see next page).

C

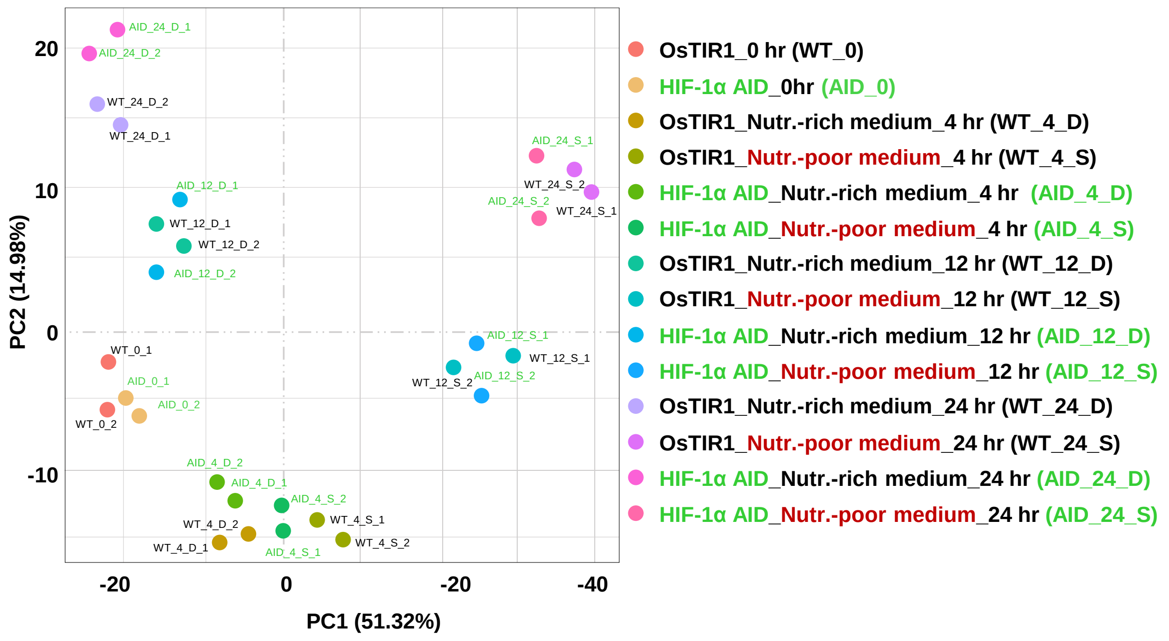

D

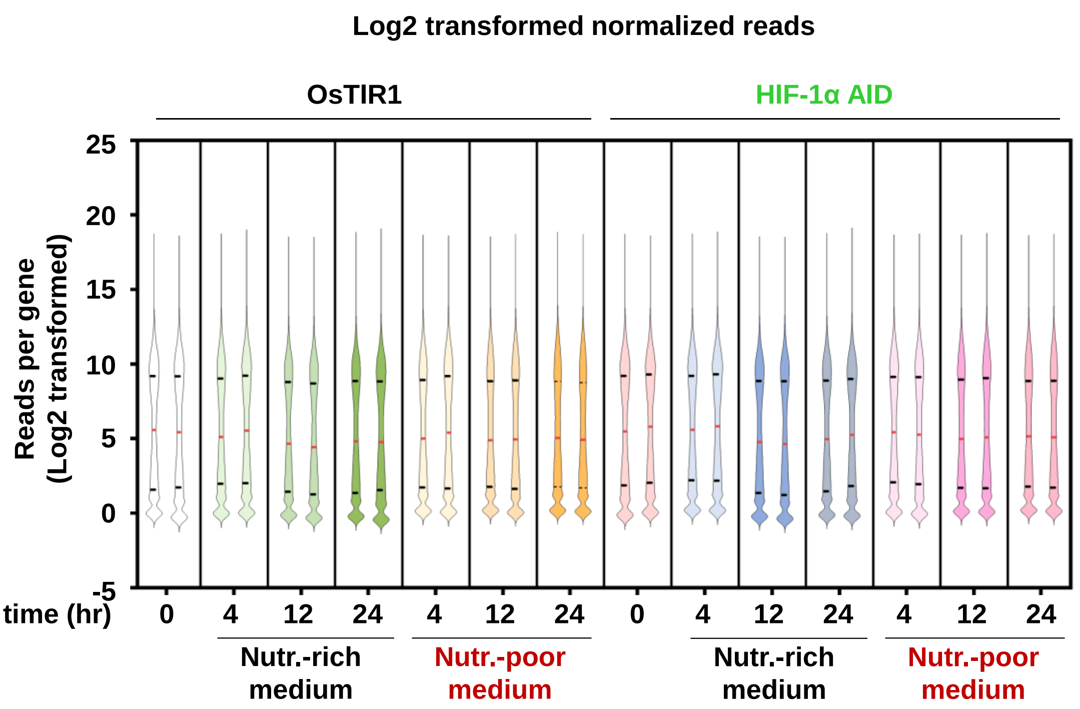

Fig. S4. Continued from previous page. Analysis of G1-HIF- and cell cycle-dependent gene expression. (C) Principal component (PC) analyses of RNA-seq data showing the first two components, samples are represented by dots. (D) Violin plots visualizing read distribution in each library after normalization. The dashed lines indicate the interquartile range containing 50% of the values around the median (red line) (..for continuation see next page).

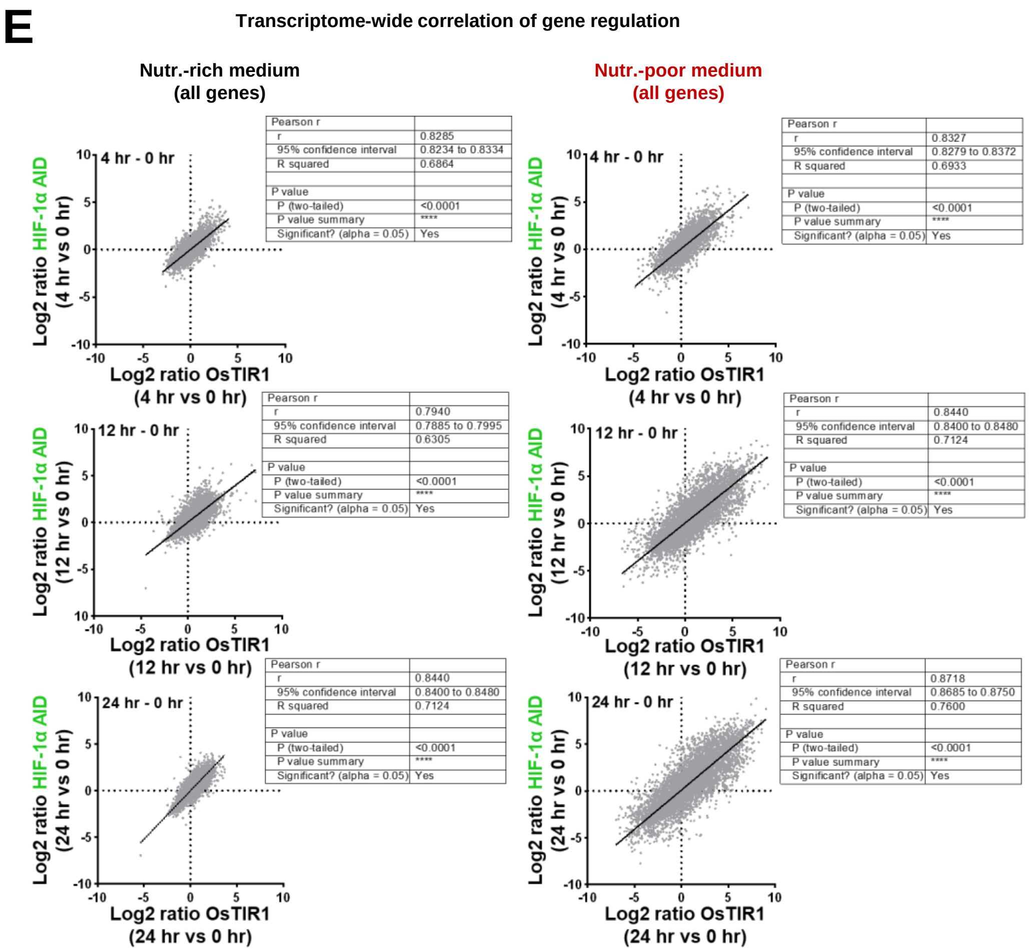

Fig. S4. Continued from previous page. Analysis of G1-HIF- and cell cycle-dependent gene expression. (E) Graphs show Pearson correlation analyses of all pairwise ratio comparisons (4/0, 12/0, 24/0 h) across the entire transcriptomic data sets. Y-axes display changes of gene expression in HCT-116 HIF-1α AID cells, while X-axes show ratios values obtained from HCT-116 OsTIR1 cells. Black lines show fitted linear regression lines. Tables display correlation coefficients (Pearson's r) and all other relevant parameters of the correlation analysis.

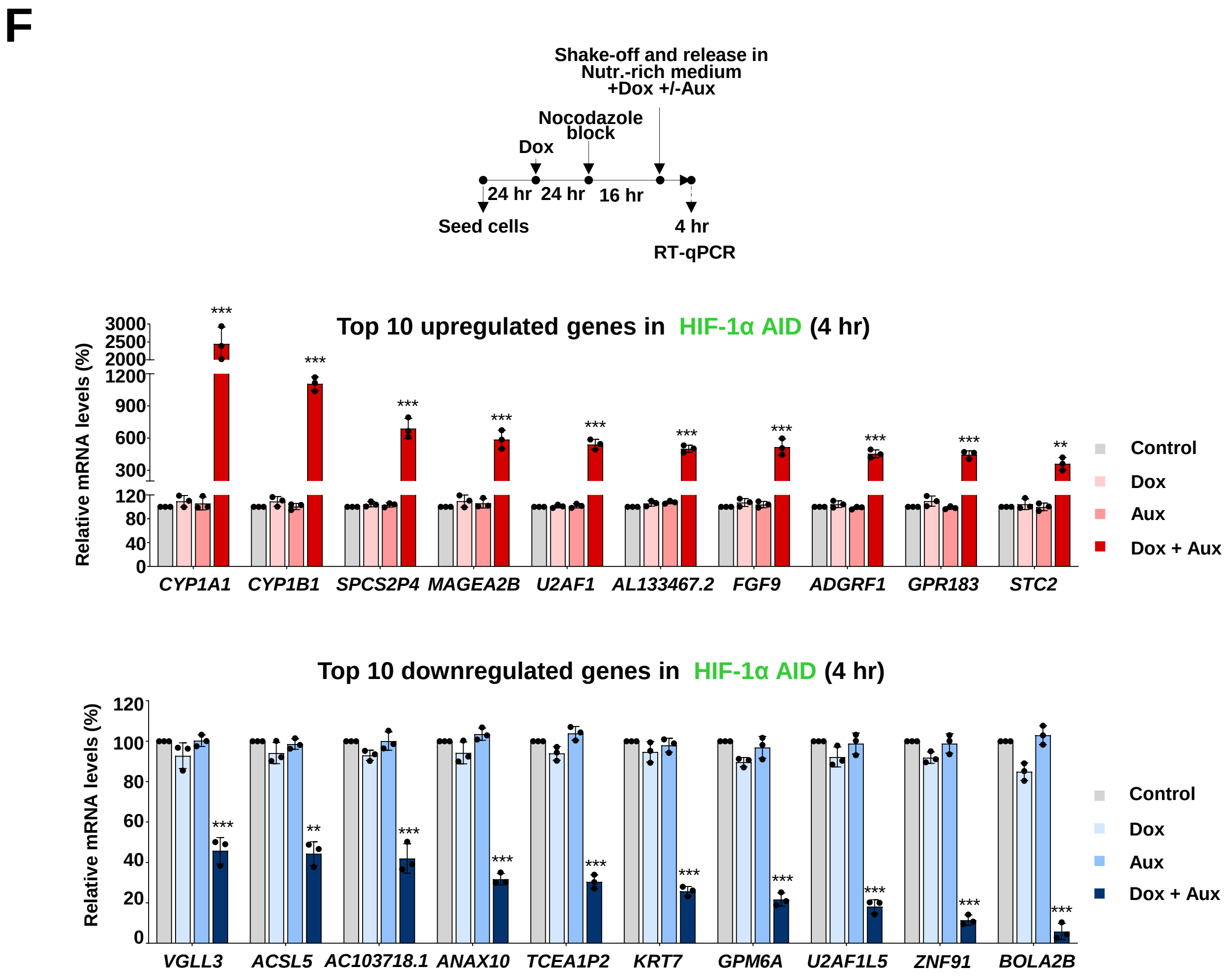

Fig. S4. Continued from previous page. Analysis of G1-HIF- and cell cycle-dependent gene expression. (F) Confirmation of HIF-1α-dependency of regulated gene expression. Cells were treated as shown and then harvested and analyzed for gene expression by RT-qPCR. The top 10 up- (red) and down-regulated (blue) genes are shown. The experiment was performed 3 times, relative expression of the control was set to 100%. Data show the mean ± SD analysed with two-way ANOVA with Tukey multiple comparisons test (*** = $P \leq 0.001$, ** = $P \leq 0.01$, n = 3).

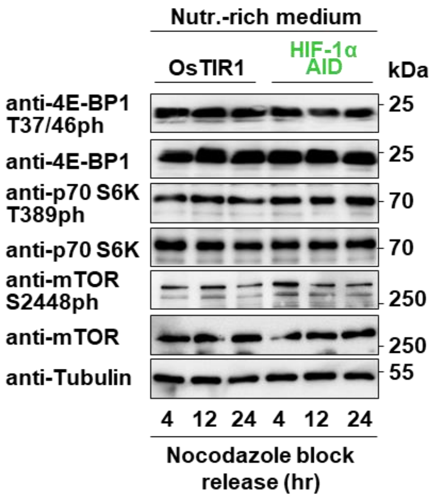

Fig. S5. Analysis of G1-HIF-mediated regulation of mTOR activity in nutrient-rich medium. HCT-116 OsTIR1 and HCT-116 HIF-1α AID cells were treated for 12 h with Dox (1 µg/mL), arrested in M phase using nocodazole and released in nutrient-rich medium containing Aux for the indicated periods. Cell extracts were analyzed by Western blotting for the expression and phosphorylation of the indicated mTOR substrates.

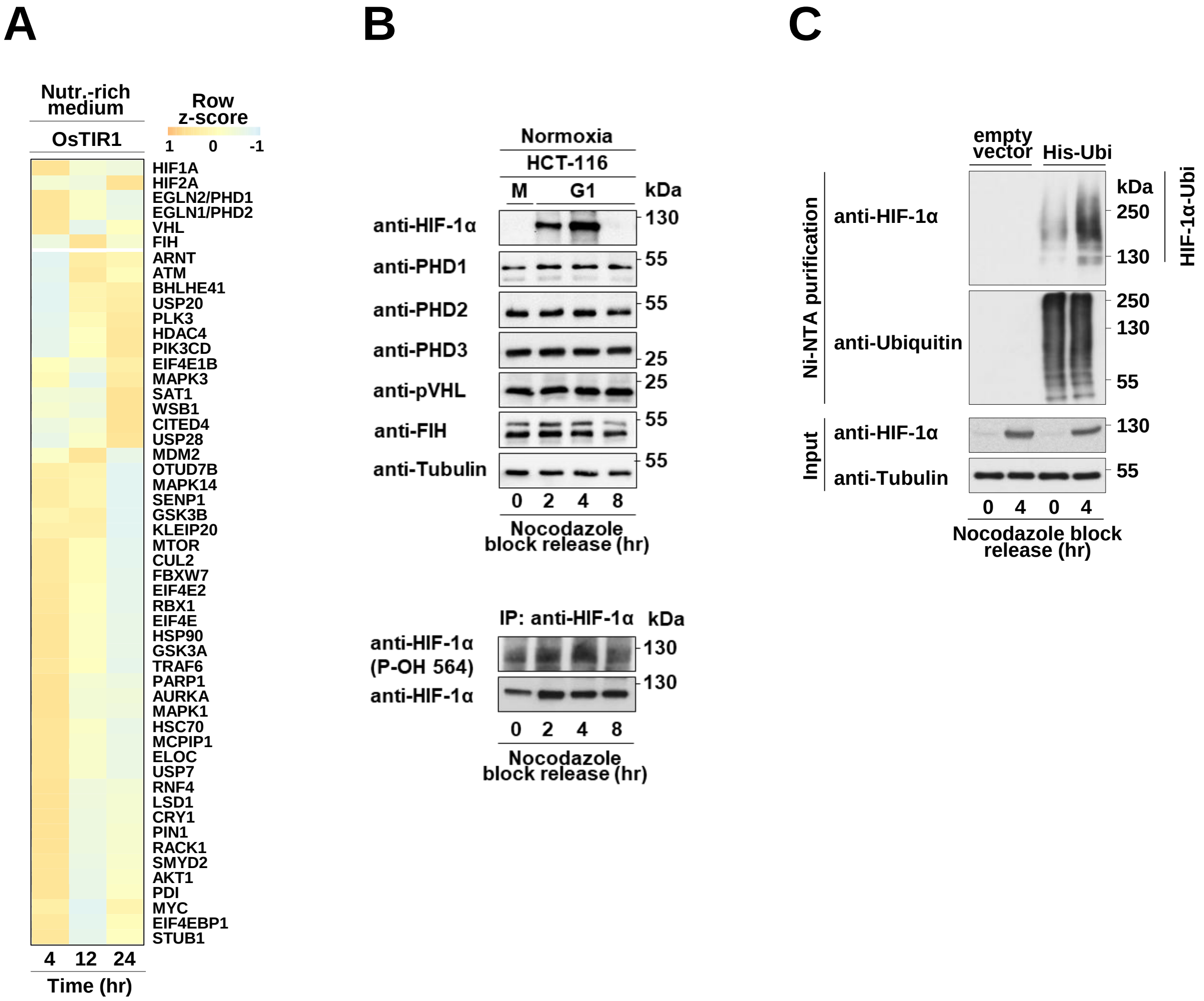

Fig. S6. Mechanisms of G1-HIF formation. (A) Known regulators of oxygen-dependent and -independent HIF-1α activation were identified by literature searches (suppl. Table S4) and interrogated for their RNA expression profile during the cell cycle. The 52 transcripts that were detected in the RNA-seq data set with $P \leq 0.05$ during at least one time point are shown, none of the transcripts showed >1.5-fold regulation. (B) Upper: HCT-116 cells were synchronized via the nocodazole block/release protocol, followed by Western blot analysis to test the expression and hydroxylation of HIF-1α and some of its regulators (M: mitosis). Lower: HCT-116 cells were synchronized as shown and HIF-1α was immunoprecipitated from lysates. The immunoprecipitated material was adjusted to obtain approximately comparable HIF-1α amounts, followed by detection of the precipitated protein and its hydroxylation with specific antibodies. (C) HCT-116 cells were transfected with a plasmid encoding (His)6-tagged ubiquitin or in the control with an empty vector as shown. The following day, cells were synchronized via nocodazole block/release. Cells were collected at the indicated time points, one fraction of the cells was lysed in NP40 lysis buffer to ensure proper upregulation of G1-HIF (Input), the remaining material was lysed under denaturing conditions, followed by enrichment of ubiquitinated proteins on nickel (Ni)-NTA beads. The ubiquitinated proteins were eluted and analyzed by immunoblotting as shown. Please note that the apparent increase of G1-HIF ubiquitination is fully reflected at the level of the input material, indicating that net ubiquitination of HIF-1α is unchanged.

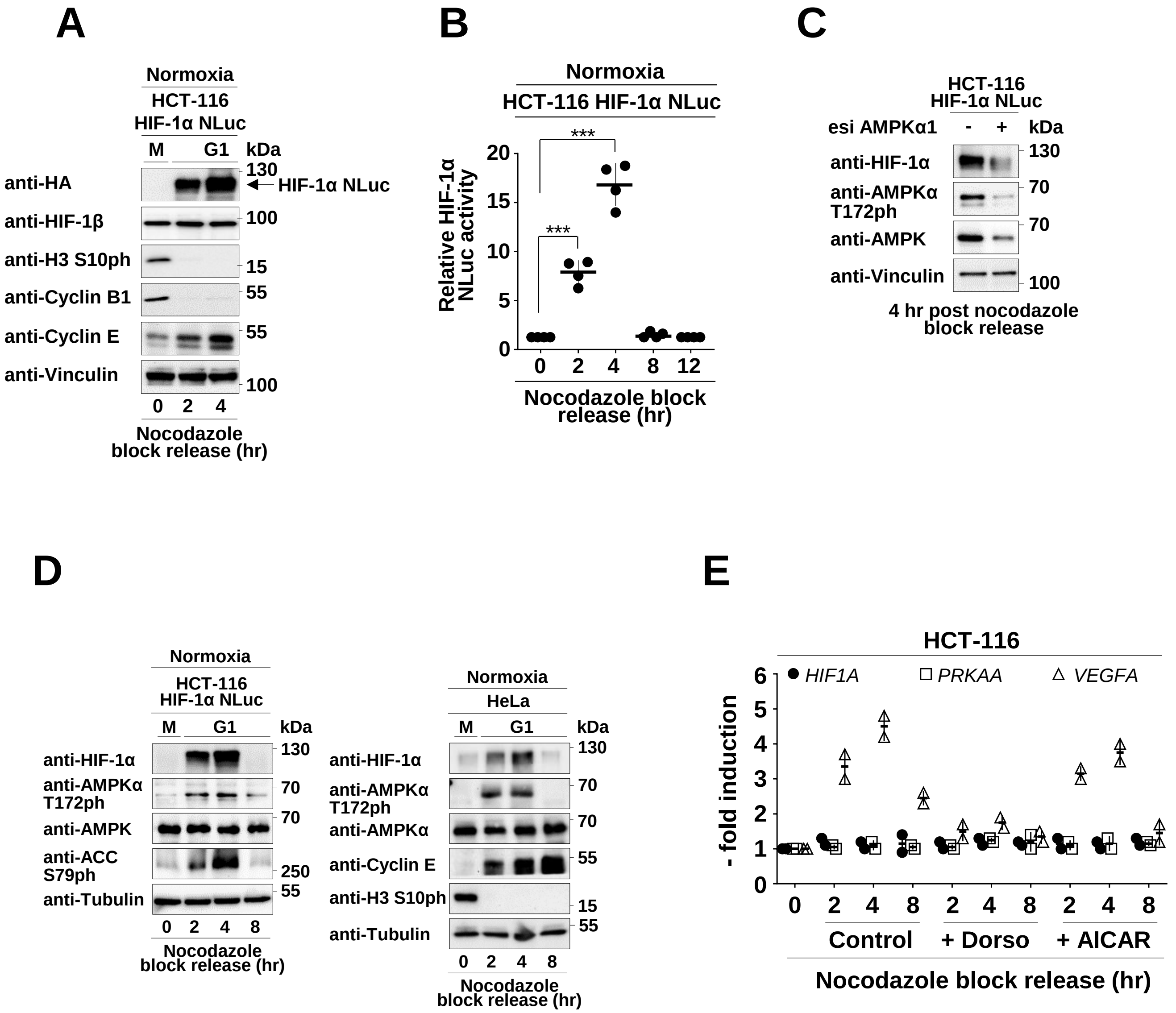

Fig. S7. Analysis of AMPK-dependent induction of G1-HIF formation. (A) HCT-116 HIF-1α NLuc cells were synchronized using the nocodazole/release protocol (upper) and analyzed at different stages of the cell cycle for the occurrence of the HIF-1α NLuc fusion protein by Western blotting (lower). (B) Cells were treated as in (A) and luciferase activity was determined at the indicated time points. Data show the mean ± SD analysed with one-way ANOVA with Bonferroni multiple comparisons test (*** = $P \leq 0.001$, n = 4). (C) HCT-116 HIF-1α NLuc cells were transfected with an esiRNA targeting AMPKα1 or a control esiRNA and synchronized using the nocodazole/release protocol. The occurrence of G1-HIF was monitored by immunoblotting as shown. (D) The indicated cells were synchronized using the nocodazole/release protocol. The occurrence of G1-HIF and the transient activation of AMPK was monitored by immunoblotting as shown. (E) HCT-116 HIF-1α NLuc cells were synchronized via nocodazole block and released in the absence and presence of Dorsomorphin (Dorso) (10 µM) or AICAR (200 µM). At the indicated time points, cells were harvested and analyzed by RT-qPCR for mRNA expression levels of the indicated genes, including the known HIF-1α target gene *VEGFA* and the gene coding for AMPKα1 (*PRKAA*). Individual data points and the mean values are indicated.

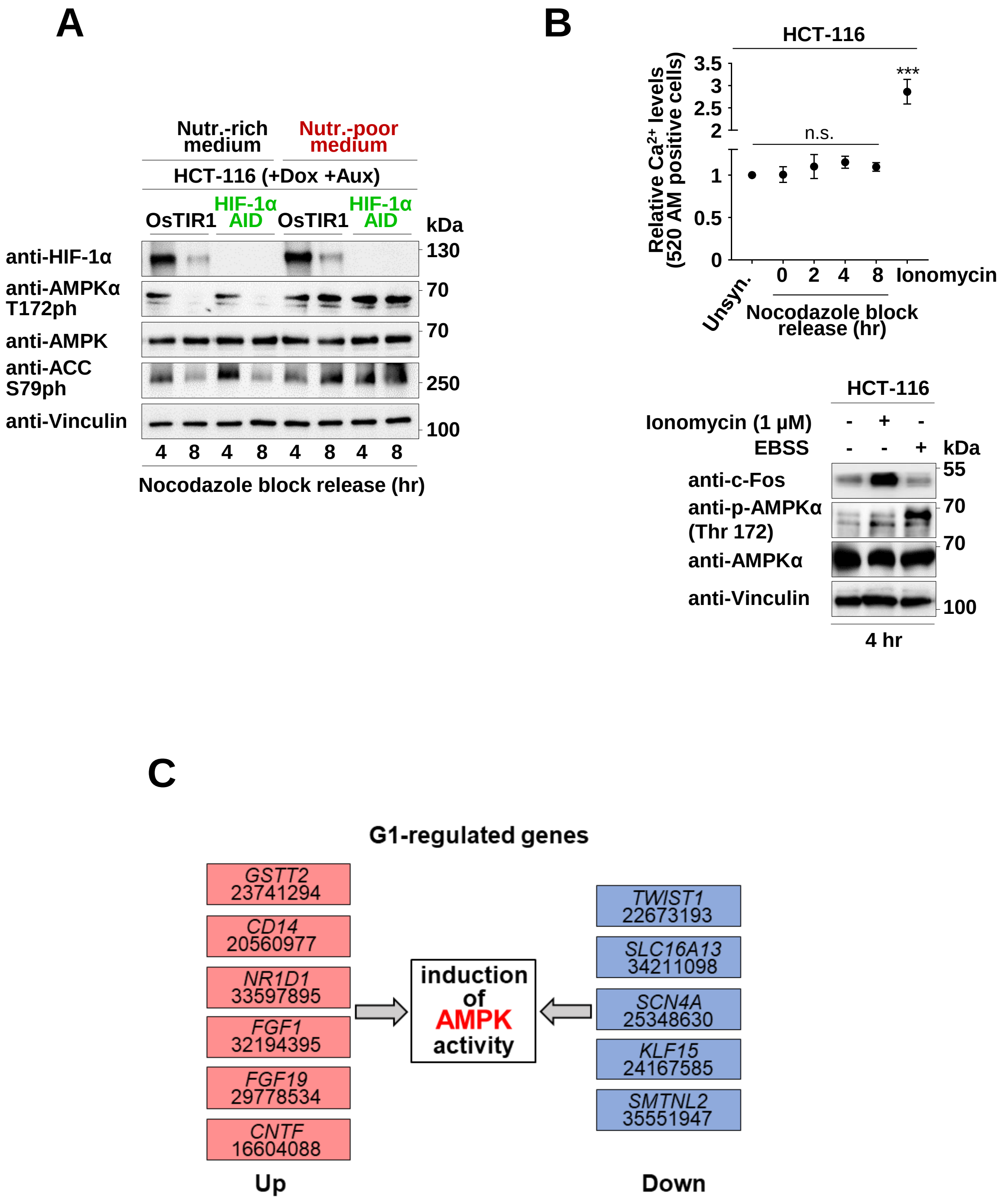

Fig. S8. Analysis of AMPK regulation in G1. (A) HCT-116 OsTIR1 and HCT-116 HIF-1α AID cells were treated with Dox for one day and then arrested in prometaphase by addition of nocodazole. Cells were released in the presence of Dox and Aux into nutrition-rich and nutrition-poor medium and analyzed at the given time points for the occurrence of G1-HIF and the activation of AMPK by immunoblotting, note that AMPK activation in the absence of G1-HIF is prolonged due to the induction of nutritional stress. (B) Upper: HCT-116 cells were synchronized via the nocodazole block/release protocol and released in the absence and presence of Ionomycin (1 µM) at the indicated time points representing different cell cycle phases. Intracellular Ca^{2+} levels were determined by FACS using the fluorogenic Ca^{2+}-sensitive dye 520 AM (Abcam). Ca^{2+} concentration of the unsynchronized HCT-116 cells were set to 1. Data are shown as mean ± SD analysed with one-way ANOVA with Tukey multiple comparisons test (n.s. = non-specific, $P \geq 0.05$, *** = $P \leq 0.001$, n = 3). Lower: HCT-116 cells were incubated with the Ca^{++} ionophore Ionomycin (1 µM) or nutrition-deficient Earle's Balanced Salt Solution (EBSS) medium for 4 h as shown. Immunoblotting was used to detect the known Ca^{2+}-regulated c-Fos protein and the activation of AMPK. (C) G1-regulated genes undergoing $\geq \pm 2$-fold ($P \leq 0.05$) regulation between Nocodazole-arrested cells and 4 h after release in nutrition-rich medium in HCT-116 OsTIR1 cells were analyzed for their ability to influence AMPK activation. The genes shown in red are upregulated in G1 and known to trigger AMPK activity. Conversely, the genes shown in blue show reduced expression in G1 and are known to trigger AMPK activation upon downregulation, the numbers show PMIDs of the relevant publications.
