# Supplementary material for: A transient increase of HIF-1α during the G1 phase (G1-HIF) ensures cell survival under nutritional stress
Source: Cell Death Dis. 2023 Jul 27;14(7):477. doi: 10.1038/s41419-023-06012-7 (PMC10374543; doi:10.1038/s41419-023-06012-7)
Supplement: Supplementary file 5 — suppl. Table 4 [file 41419_2023_6012_MOESM5_ESM.docx]

**A transient increase of HIF-1α during the G1 phase (G1-HIF) ensures cell survival under nutritional stress**

Ratnal Belapurkar, Max Pfisterer, Jan Dreute, Sebastian Werner, Sven Zukunft, Ingrid Fleming, Michael Kracht and M. Lienhard SCHMITZ

**Supplementary table S4.** Further information on the inhibitor screen and siRNA screen displayed in Fig. 8B, C.

| **Protein name (Symbol)** | **Inhibitors (Source)** | **Working conc.(µM)** | **Relevance to HIF-1α pathway** | **PMID** |
| --- | --- | --- | --- | --- |
| AMP-activated protein kinase (AMPK) | Dorsomorphin 2HCl (Selleckchem #S7306) | 10 | Indirect interaction with HIF1-α | **2606143127259535** |
| ATM serine/threonine-protein kinase (ATM) | KU55933 (Selleckchem #S1092) | 10 | Phosphorylation on Ser 696 results in stabilization of HIF1-α | **21095582** |
| Aurora Kinase A (Aurora A) | Alisertib/MLN8237 (Selleckchem #S1133) | 20 | Promote HIF1-α dependent signaling | 23966163 |
| Calcineurin | Cyclosporine A (Sigma #33024) | 1 | Inhibit RACK1 mediated HIF1-α degradation | **17965024** |
| c-Jun N-terminal kinase 1 (JNK1) | Sp600125 (Selleckchem #S1460) | 10 | Mediates degradation of HIF1-α in a pVHL-independent manner | **20068160** |
| Cyclin-dependent kinase 5 (CDK5) | sc-202094 (Santacruz #sc-202094) | 10 | Phosphorylation on Ser 687 results in stabilization of HIF1-α | **28336293** |
| Glycogen synthase kinase 3 (GSK3) | BIO (Sigma #B1686) | 20 | Phosphorylation on Ser 551,555 and 589 targets HIF1-α to proteasomal degradation | **12764143** |
| Heat Shock Protein 90(HSP90) | 17-Geldanamycin (Selleckchem #S2713) | 30 | Inhibits ubiquitination by RACK1 and stabilizes HIF1-α | **17244529** |
| Histone Deacetylase 4 (HDAC4) | LMK-235 (Sigma #SML-1053) | 20 | Deacetylation at Lysine 10,11,12,19 and 21 results in stabilization of HIF1-α | **21917920** |
| Lysine-specific histone demethylase 1A (LSD1) | SP2509 (Selleckchem #S7680) | 10 | Demethylation of K32 residue results in stabilization of HIF1-α | **26757928** |
| Mammalian target of rapamycin complex (mTORC1/2) | AZD8055 (Selleckchem  #S1555) | 20 | Enhances transcription rate of HIF1-α resulting in HIF1-α Protein accumulation | **16915281** |
| NAD-dependent deacetylase sirtuin 7(SIRT7) | Nicotinamide (Selleckchem  #S1899) | 30 | Degradation of HIF1-α independent of PHDs and proteasomal degradation | **23750001** |
| p38 mitogen-activated protein kinases (p38 MAPK) | SB-202190 (Sigma #S7067) | 50 | Stabilization and transactivation of HIF1-α | **16278378** |
| Peptidyl-prolyl cis/trans isomerase (PIN1) | piB – ATRA (Sigma #B7688) | 10 | Direct interaction and stabilization of HIF-1α | 26964091 |
| Phosphoinositide 3-kinases (PI3K) | LY294002 (CST #9901s) | 50 | Non-conventional stabilization of HIF1-α | **16289155** |
| Polo Like Kinase 3 (PLK3) | BI 6727 (Selleckchem #S2235) | 1 | Phosphorylation of Ser576 and Ser657 residues and degradation of HIF1-α | **20889502** |
| Protein kinase B (AKT) | MK-2206 2HCl (Selleckchem #S1078) | 5 | HIF1-α protein accumulation | **16289155** |
| Ras-Raf-MEK-ERK pathway (ERK/MAPK) | PD98059 (Selleckchem #S1177) | 20 | P300 mediated transactivation of HIF1-α | **16289155** |
| SUMO Specific Peptidase 1 (SENP1) | Momordin Ic (Selleckchem #S9159) | 10 | DeSUMOylation and stabilization of HIF1-α | **17981124** |
| Ubiquitin C-Terminal Hydrolase L1 (UCHL1) | LDN57444 (Sigma #L4170) | 5 | Deubiquitylation and stabilization of HIF1-α | **27014628** |
| Ubiquitin Specific Peptidase 20 (USP20) | GSK2643943A (Medchem #HY-11458) | 10 | Deubiquitylation and stabilization of HIF1-α by removing pVHL mediated ubiquitination | **27014628** |
| Ubiquitin Specific Peptidase 28 (USP28) | AZ1 (Selleckchem  #S8904) | 5 | Deubiquitylation and stabilization of HIF1-α by inhibiting Fbw7-GSK3 mediated degradation | **31208103** |
| Ubiquitin Specific Peptidase 7 (USP7) | P5091 (Selleckchem #S7132) | 1 | Deubiquitylation and stabilization of HIF1-α | **31208103** |

**List of genes inhibited using esiRNA mediated knockdown in this study (Fig 8C):**

| **Gene name**  **(Symbol)** | **Genecard name** | **Relevance to HIF-1α pathway** | **PMID** |
| --- | --- | --- | --- |
| AMP-activated protein kinase (AMPK) | *PRKAA2* | Stimulation of HIF1-α transcriptional activity/controversial | **26061431,27259535** |
| Basic Helix-Loop-Helix Family Member E41 (BHLHE41) | *BHLHE41* | Direct interaction and degradation of HIF1-α via the proteasomal pathway | **22801492** |
| Cellular myelocytomatosis oncogene (c-Myc) | *MYC* | Stabilization of HIF1-α in normoxia and hypoxia | **22186139** |
| Cryptochrome Circadian Regulator 1(CRY1) | *CRY1* | Modulation of promoter binding of HIF1-α and reduction of the half-life of HIF1-α protein | **30875610** |
| Glycogen synthase kinase 3 (GSK3) | *GSK3A* | Degradation of HIF1-α via a pVHL-independent mechanism | **17325032** |
| Kelch Like Family Member 20 (KLEIP20) | *KLHL20* | Stabilization of HIF1-α mRNA | **31208103** |
| Lysosome-associated membrane protein 2 (LAMP2) | *LAMP2* | Degradation of HIF1-α via Hsp70 interaction axis | **23457305** |
| Monocyte chemotactic protein-1-induced protein-1 (MCPIP1) | *ZC3H12A* | Deubiquitylation and stabilization of HIF1-α | **29742804** |
| OTU Domain Containing 7B (Cezanne) | *OTUD7B* | Stabilization of HIF1-α via a proteasome-independent mechanism | **25355043** |
| Receptor for Activated C Kinase 1 (RACK1) | *RACK1* | Direct interaction and degradation of HIF1-α | **17244529** |
| Septins (SEPT) | *SEPTIN9* | Inhibition of RACK1 mediated HIF1-α degradation | **19251694** |
| Spermidine/Spermine N1-Acetyltransferase 1 (SAT1) | *SAT1* | Degradation of HIF1-α via RACK1 mediated pathway | **17875644** |
| STIP1 Homology And U-Box Containing Protein 1 (STUB1) | *STUB1* | Degradation of HIF1-α via Hsp70 interaction axis | **23880665** |
| Tumor necrosis factor receptor (TNFR)-associated factor 6 (TRAF6) | *TRAF6* | K63 linked poly-ubiquitination and stabilization of HIF1-α | **23722539** |
| Ubiquitin C-Terminal Hydrolase L1(UCHL1) | *UCHL1* | Deubiquitylation and stabilization of HIF1-α | **27014628** |
| Ubiquitin Specific Peptidase 19 (USP19) | *USP19* | Stabilization of HIF1-α via PHDs/SIAHs interaction | **27014628** |
| Ubiquitin Specific Peptidase 7(USP7) | *USP7* | Deubiquitylation and stabilization of HIF1-α | **31208103** |
| Ubiquitin-specific peptidase 52/poly(A) nuclease 2 (USP52) | *PAN2* | Stabilization of HIF1-α mRNA allowing normoxic induction of HIF1-α | **27014628** |
