# Supplementary material for: A transient increase of HIF-1α during the G1 phase (G1-HIF) ensures cell survival under nutritional stress
Source: Cell Death Dis. 2023 Jul 27;14(7):477. doi: 10.1038/s41419-023-06012-7 (PMC10374543; doi:10.1038/s41419-023-06012-7)
Supplement: Supplementary file 6 — suppl. Table 5 [file 41419_2023_6012_MOESM6_ESM.docx]

**A transient increase of HIF-1α during the G1 phase (G1-HIF) ensures cell survival under nutritional stress**

Ratnal Belapurkar, Maximilian Pfisterer, Jan Dreute, Sebastian Werner, Sven Zukunft, Ingrid Fleming, Michael Kracht and M. Lienhard SCHMITZ

**Suppl. Table S5**

**List of antibodies, plasmids, oligonucleotides, reagents and kits used in this study:**

**Antibodies**

| **Primary Antibody** | **Source** | **Identifier** | **Dilution** |
| --- | --- | --- | --- |
| AMPKα Thr 172ph (40H90)  rabbit mAb | Cell Signaling Technology | Cat #2531S RRID:AB_330330 | 1:1000 |
| AMPKα  rabbit mAb | Cell Signaling Technology | Cat #2532S RRID:AB_330331 | 1:1000 |
| Aurora B  mouse mAb | Abcam | Cat #ab3609 RRID:AB_449204 | 1:1000 |
| c-Fos (9F6)  rabbit mAb | Cell Signaling Technology | Ca t#2250 RRID:AB_2247211 | 1:1000 |
| Cleaved Caspase 3 Asp 175 rabbit mAb | Cell Signaling Technology | Cat #9664 RRID:AB_2070042 | 1:1000 |
| Cyclin B1 (D11)  mouse mAb | Santa Cruz | Cat #sc-7393 RRID:AB_626632 | 1:1000 |
| Cyclin D (92G2)  rabbit mAb | Cell Signaling Technology | Cat #2978 RRID:AB_2259616 | 1:1000 |
| Cyclin E (HE12)  mouse mAb | Santa Cruz | Cat #sc-247 RRID:AB_627357 | 1:1000 |
| 4E-BP1 (52H11)  rabbit mAb | Cell Signaling Technology | Cat #9644 RRID:AB_2097841 | 1:1000 |
| 4E-BP1 Thr 37/46ph (236B4)  rabbit mAb | Cell Signaling Technology | Cat #2855 RRID:AB_560835 | 1:1000 |
| FIH (D19B3)  rabbit mAb | Cell Signaling Technology | Cat #4426S RRID:NA | 1:1000 |
| H2A.X S139ph (JBW301)  mouse mAb | Millipore | Cat #05-636 RRID:AB_309864 | 1:1000 |
| H3 Ser10-P (6G3)  mouse mAb | Cell Signaling Technology | Cat #9706S RRID:AB_331748 | 1:1000 |
| HA (3F10)  rat mAb | Roche Sigma Aldrich | Cat #4426S RRID:AB_390918 | 1:1000 |
| HIF-1α (D2U3T)  rabbit mAb | Cell Signaling Technology | Cat #14179 RRID:AB_2622225 | 1:500 |
| HIF-1α Pro 564OH (D43B5)  rabbit mAb | Cell Signaling Technology | Cat #3434 RRID:AB_2116958 | 1:500 |
| HIF-1β (D28F3)  rabbit mAb | Cell Signaling Technology | Cat #5537 RRID:AB_10694232 | 1:1000 |
| HIF-2α  rabbit pAb | Novus Biologicals | Cat #NB100-122SS RRID:AB_10002593 | 1:500 |
| HIF-3α  mouse pAb | Novus Biologicals | Cat #H00064344-B02P RRID:AB_1237045 | 1:500 |
| LC3B (D11)  rabbit mAb | Cell Signaling Technology | Cat #3868S RRID:AB_2137707 | 1:1000 |
| mTOR (7C10)  rabbit mAb | Cell Signaling Technology | Cat #2972S RRID:AB_330978 | 1:1000 |
| mTOR Ser 2448ph  rabbit mAb | Cell Signaling Technology | Cat #5536T RRID:AB_10691552 | 1:1000 |
| p21WAF1 (EA10)  mouse mAb | Calbiochem | Cat #OP64 RRID:AB_2335868 | 1:1000 |
| p62 (D3)  mouse mAb | Santa Cruz | Cat #sc-28359 RRID:AB_628279 | 1:1000 |
| pACC Ser 79ph  rabbit mAb | Cell Signaling Technology | Cat #3661 RRID:AB_330337 | 1:1000 |
| p70S6K  rabbit mAb | Cell Signaling Technology | Cat #9202S RRID:AB_331676 | 1:1000 |
| p70S6K Thr 389ph  rabbit mAb | Cell Signaling Technology | Cat #9205S RRID:AB_330944 | 1:1000 |
| PARP (C210)  mouse mAb | Clontech  Takara Bio | Cat #630210 RRID: NA | 1:1000 |
| PFKFB3  rabbit pAb | Proteintech | Cat #13763-1-AP RRID:AB_2162854 | 1:500 |
| PHD1  rabbit pAb | Novus Biologicals | Cat #NB100-310 RRID:AB_10001800 | 1:1000 |
| PHD2  rabbit pAb | Novus Biologicals | Cat #NB100-137SS RRID:AB_791551 | 1:1000 |
| PHD3  rabbit pAb | Novus Biologicals | Cat #NB100-139SS RRID:AB_2246335 | 1:1000 |
| pVHL  rabbit pAb | Cell Signaling Technology | Cat #68547 RRID:AB_2716279 | 1:1000 |
| Ubiquitin (P4D1)  mouse mAb | Cell Signaling Technology | Cat #3936 RRID:AB_331292 | 1:1000 |
| Vinculin  mouse mAb | Sigma | Cat #V9131 RRID:AB_477629 | 1:1000 |
| α-Tubulin (12G10)  mouse mAb | DSHB | Cat #12G10 RRID:AB_1157911 | 1:1000 |
| **Secondary Antibody** | **Source** | **Identifier** | **Dilution** |
| goat-anti-mouse IgG HRP | Dianova | Cat #115-035-146 RRID:AB_2307392 | 1:5000 |
| goat-anti-rabbit IgG HRP | Dianova | Cat #111-035-144 RRID:AB_2307391 | 1:5000 |
| goat-anti-rat IgG HRP | Dianova | Cat #112-035-143 RRID:AB_2338138 | 1:5000 |

**Plasmids**

| **Plasmid** | **Source** | **Identifier** |
| --- | --- | --- |
| Cas9 (pX459) | Addgene | PMID: 24157548 Cat #48139 RRID: Addgene_48139 |
| Cas9 sg RNA PITCh (pUC ori) | Dr. Tetsushi Sakuma, Dr. Takashi Yamamoto | PMID: **26678082** |
| Cas9 sgHIF1α C term (pX459) | M.L. Schmitz | This study |
| HIF-1α-mAID (pUC57) | M.L. Schmitz | This study |
| HIF-1α-Nano Luciferase (pUC57) | M.L. Schmitz | This study |
| His6-Ubiquitin (His-Ub_8_/pCIN4) | Dr. Richard Baer | PMID: 15166217 |

**DNA- Oligonucleotide for CRISPR/Cas9-mediated HIF-1α genome engineering**

| **Oligonucleotides** | **Sequence (5’-3’)** | **Purpose** |
| --- | --- | --- |
| hu HIF-1α-sgRNA-for | CACCGAAGAATTACTCAGAGCTT | pX459 cloning |
| hu HIF-1α-sgRNA-rev | AAACAAGCTCTGAGTAATTCTTC | pX459 cloning |
| HIF-1α-mAID (pUC57) | AAGGAGAAGAGTGCTTGTCCTAAAGATCCAGCCAAACCTCCGGCCAAGGCACAAGTTGGGGATGGCCACCGGTGAGATCATACCGGAAGAACGTGATGGTTTCCTGCCAAAAATCAAGCGGTGGCCCGGAGGCGGCGGCGTTCGTGAAGGTATCAATGGACGGAGCACCGTACTTGAGGAAAATCGATTTGAGGATGTATAAA | Repair template for Endogenous tagging of HIF-1α with AID |
| HIF-1α-Nano Luciferase (pUC57) | GTCTTCACACTCGAAGATTTCGTTGGGGACTGGCGACAGACAGCCGGCTACAACCTGGACCAAGTCCTTGAACAGGGAGGTGTGTCCAGTTTGTTTCAGAATCTCGGGGTGTCCGTAACTCCGATCCAAAGGATTGTCCTGAGCGGTGAAAATGGGCTGAAGATCGACATCCATGTCATCATCCCGTATGAAGGTCTGAGCGGCGACCAAATGGGCCAGATCGAAAAAATTTTTAAGGTGGTGTACCCTGTGGATGATCATCACTTTAAGGTGATCCTGCACTATGGCACACTGGTAATCGACGGGGTTACGCCGAACATGATCGACTATTTCGGACGGCCGTATGAAGGCATCGCCGTGTTCGACGGCAAAAAGATCACTGTAACAGGGACCCTGTGGAACGGCAACAAAATTATCGACGAGCGCCTGATCAACCCCGACGGCTCCCTGCTGTTCCGAGTAACCATCAACGGAGTGACCGGCTGGCGGCTGTGCGAACGCATTCTGGCG | Repair template for Endogenous tagging of HIF-1α with Nano Luciferase |

**DNA- Oligonucleotide for qPCR**

| **Oligonucleotides** | **Sequence (5’-3’)** |
| --- | --- |
| hu ANKRD37-for | CTGGAGTGCCTAAGCCTGCTTGTA |
| hu ANKRD37-rev | CTCCGTTTCTGTCTGAGC ACGGC |
| hu HIF-1α-for | CACCACAGGACAGTACAGGAT |
| hu HIF-1α-rev | CGTGCTGAATAATACCACTCACA |
| hu TPI-for | GGACTCGGAGTAATCGCCTG |
| hu TPI-rev | TGTTGGGGTGTTGCAGTCTT |
| hu VEGF-for | CGCTATGTGACCTTTGGCATTT |
| hu VEGF-rev | GCTAAAGTCAGGGCCACTTACT |

**Reagents**

| **Reagent** | **Source** | **Identifier** | **Working conc.** |
| --- | --- | --- | --- |
| 3-Indole acetic acid (Auxin) | Sigma | Cat #I3750 | 100 µM/ml |
| 5-Aminoimidazole-4-carboxamide ribonucleotide (AICAR) | Sigma | Cat #A9978 | 100-200 µM/ml |
| 5-Bromo-4-chloro-3-indoyl-D-galactopyranoside (X-Gal) | Sigma | Cat #16555 | 1 mg/ml |
| Bafilomycin A | InvivoGen | Cat #tlrl-baf1 | 100 nM/ml |
| Blasticidin | InvivoGen | Cat #ant-bl-1 | 7 µg/ml |
| Bleomycin | Santa Cruz | Cat # sc-200134 | 10 µg/ml |
| Desferrioxamine (DFO) | Sigma | Cat #D9533 | 100 µM/ml |
| Doxycycline | Sigma | Cat #D9891 | 1 µg/ml |
| H_2_DCFDA | Invitrogen | Cat #D399 | 1 µM |
| Ionomycin | Sigma | Cat # I0634 | 1 µM/ml |
| MG-132 (Z-Leu-Leu-Leu-al) | Sigma | Cat #SML1135 | 10 µM/ml |
| MitoTracker Deep Red FM | Invitrogen | Cat #M22426 | 200 nM |
| Nocodazole | Sigma | Cat #M1404 | 0.1 µg/ml |
| Quinoline-Val-Asp-Difluorophenoxymethylketone  (Q-VD-OPh/Q-VD) | Selleckchem | Cat #S7311 | 20 µM/ml |
| Thymidine | Sigma | Cat #T1895 | 2 mM/ml |

**Kits**

| **Reagent** | **Source** | **Identifier** |
| --- | --- | --- |
| Annexin V (FITC) Apoptosis Detection | eBioscience | Cat #88-8005-72 |
| Calcium Flux Assay Kit | Abcam | Cat #ab233472 |
| Nano -Glo Luciferase Assay | Promega | Cat #N1130 |
| NucleoSpin RNA | Macherey-Nagel | Cat # 740955.50 |
| NucleoSpin Tissue | Macherey-Nagel | Cat #740952.50 |
| Seahorse XF Cell Mito Stress Test | Agilent | Cat#103015-100 |

**Cell culture medium**

| **Medium** | **Source** | **Identifier** |
| --- | --- | --- |
| DMEM, high glucose + GlutaMAX | Life Technologies | Cat # 31966-047 |
| DMEM, no glucose, no glutamine, no phenol red | Life Technologies | Cat #A1443001 |
| huFIB Medium | InSCREENeX GmbH | Cat #INS-ME-1001 |
