# Supplementary material for: A transient increase of HIF-1α during the G1 phase (G1-HIF) ensures cell survival under nutritional stress
Source: Cell Death Dis. 2023 Jul 27;14(7):477. doi: 10.1038/s41419-023-06012-7 (PMC10374543; doi:10.1038/s41419-023-06012-7)

A transient increase of HIF-1α during the G1 phase (G1-HIF) controls amino acid homeostasis to ensure cell survival under nutritional stress

Ratnal Belapurkar, Maximilian Pfisterer, Jan, Dreute, Sebastian Werner, Sven Zukunft, Ingrid Fleming, Michael Kracht and M. Lienhard SCHMITZ

Original blots

Fig. 1A

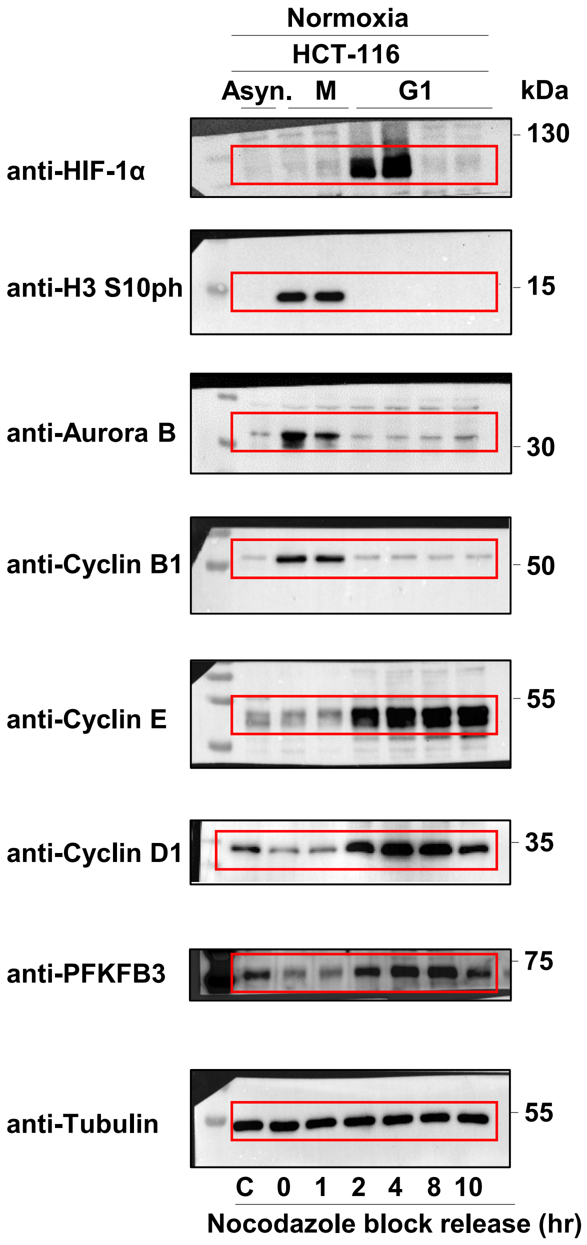

Fig. 1B

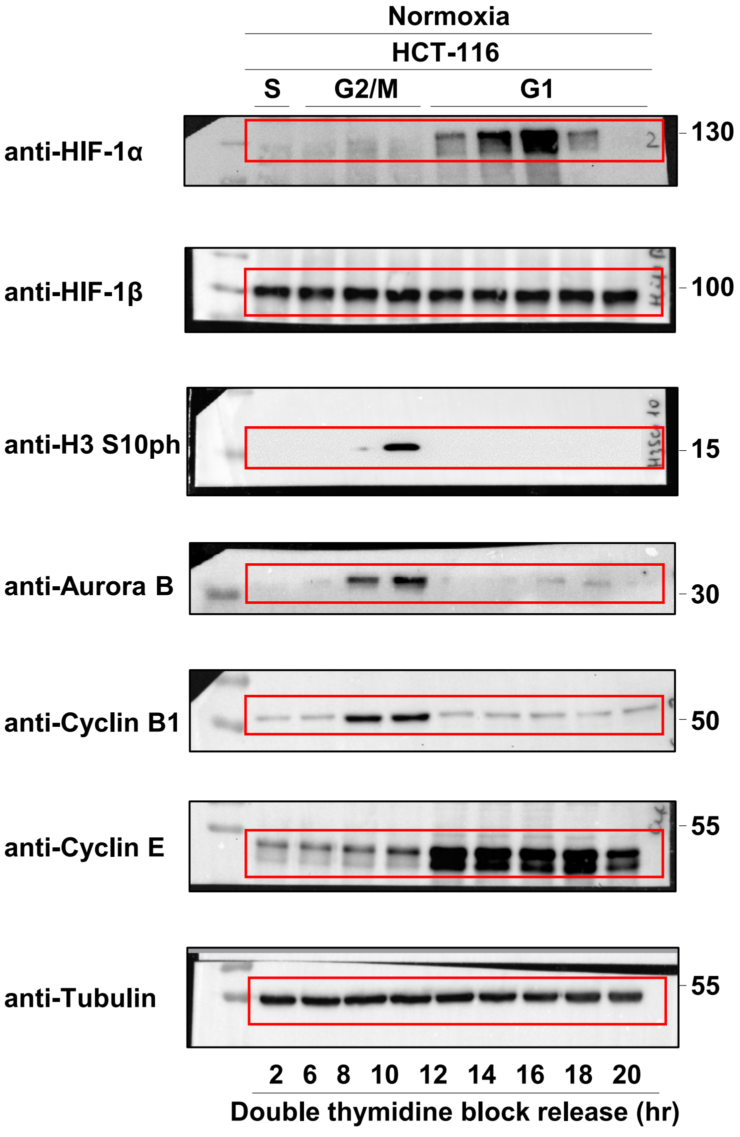

Normoxia
HCT-116
S
G2/M
G1
anti-HIF-1α
130
anti-HIF-1β
100
anti-H3 S10ph
15
anti-Aurora B
30
anti-Cyclin B1
50
anti-Cyclin E
55
anti-Tubulin
55
2 6 8 10 12 14 16 18 20
Double thymidine block release (hr)

Fig. 1C

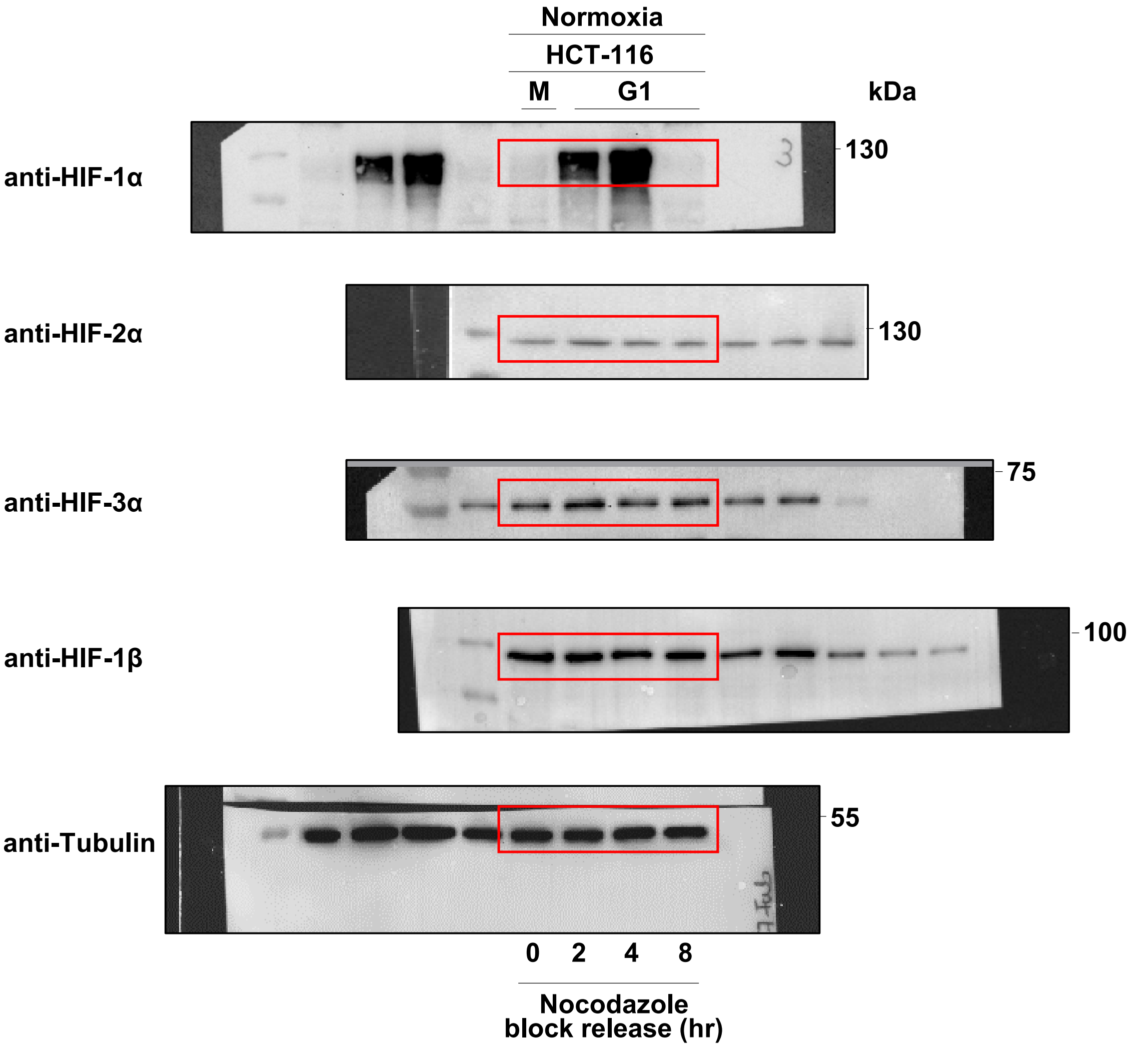
Normoxia
HCT-116
M
G1
kDa
anti-HIF-1α
130
anti-HIF-2α
130
anti-HIF-3α
75
anti-HIF-1β
100
anti-Tubulin
55
0
2
4
8
Nocodazole
block release (hr)

Fig. 1D

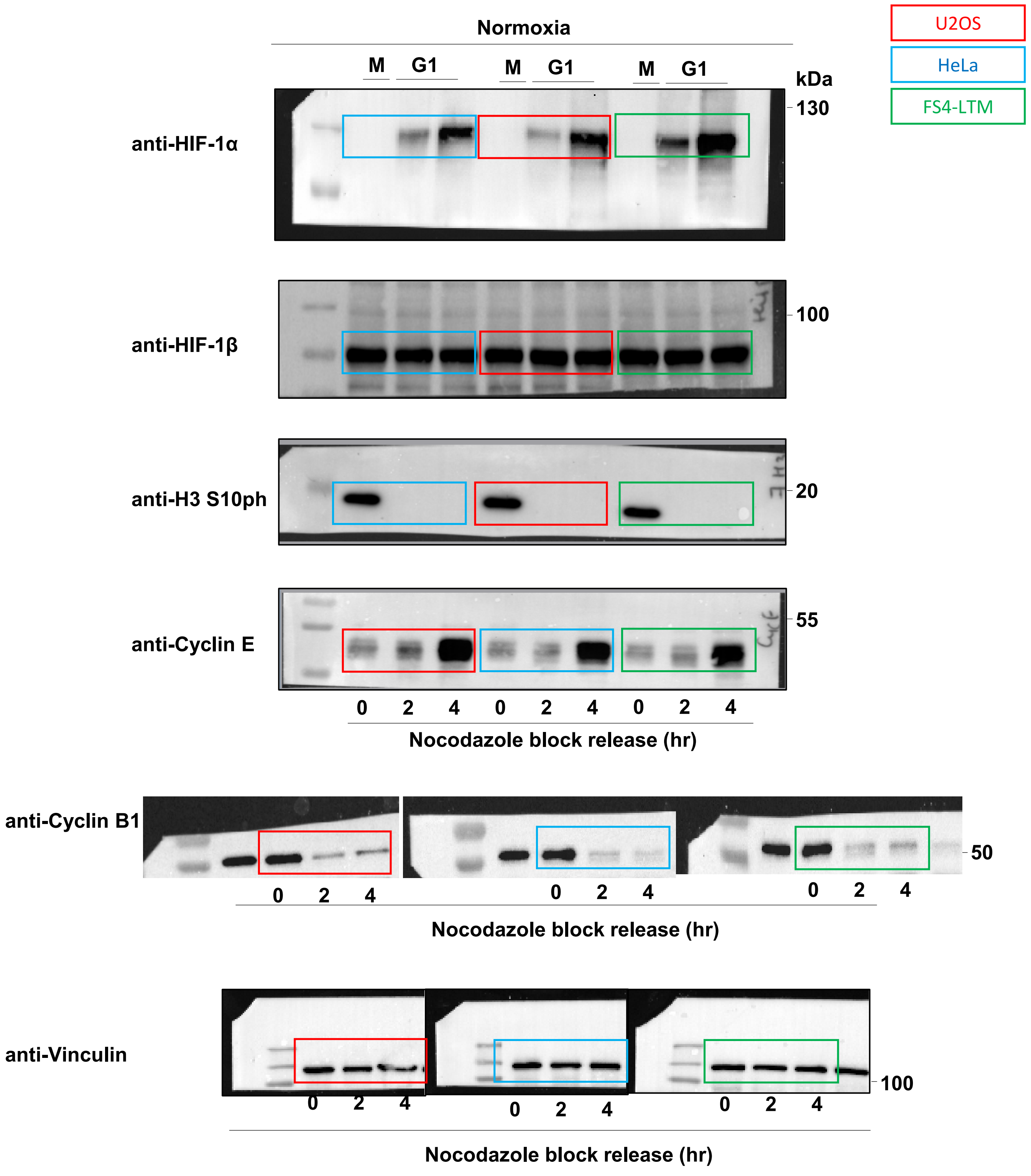

Normoxia
M
G1
M
G1
M
G1
kDa
130
100
20
55
50
100
U2OS
HeLa
FS4-LTM
anti-HIF-1α
anti-HIF-1β
anti-H3 S10ph
anti-Cyclin E
0 2 4 0 2 4 0 2 4
Nocodazole block release (hr)
anti-Cyclin B1
0 2 4
0 2 4
0 2 4
Nocodazole block release (hr)
anti-Vinculin
0 2 4
0 2 4
0 2 4
Nocodazole block release (hr)

Fig. 1E

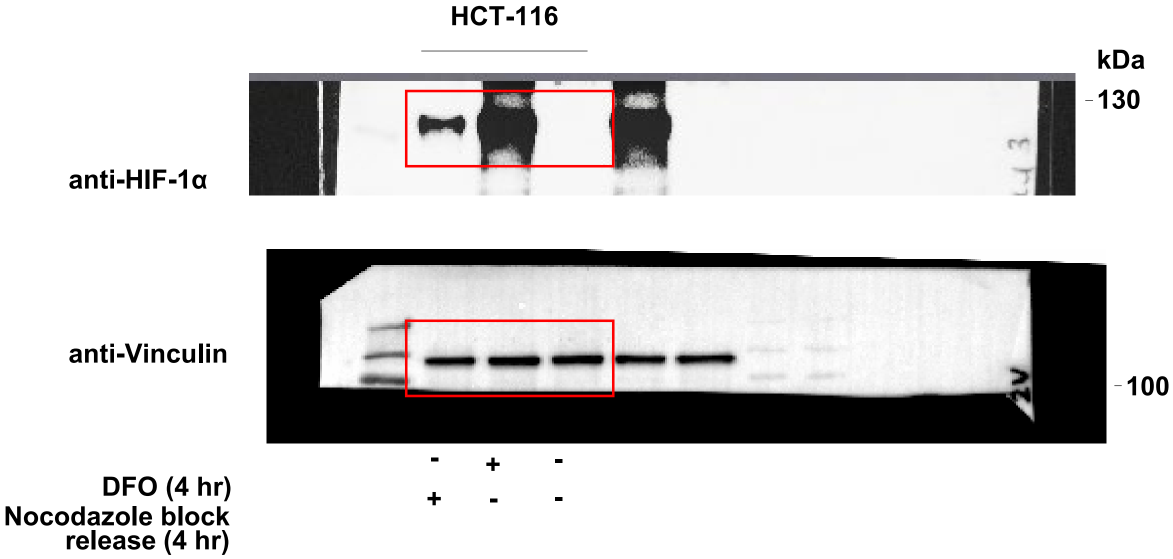
HCT-116
kDa
130
anti-HIF-1α
anti-Vinculin
100
DFO (4 hr)
Nocodazole block
release (4 hr)
- + -
+ - -

Fig. 2A

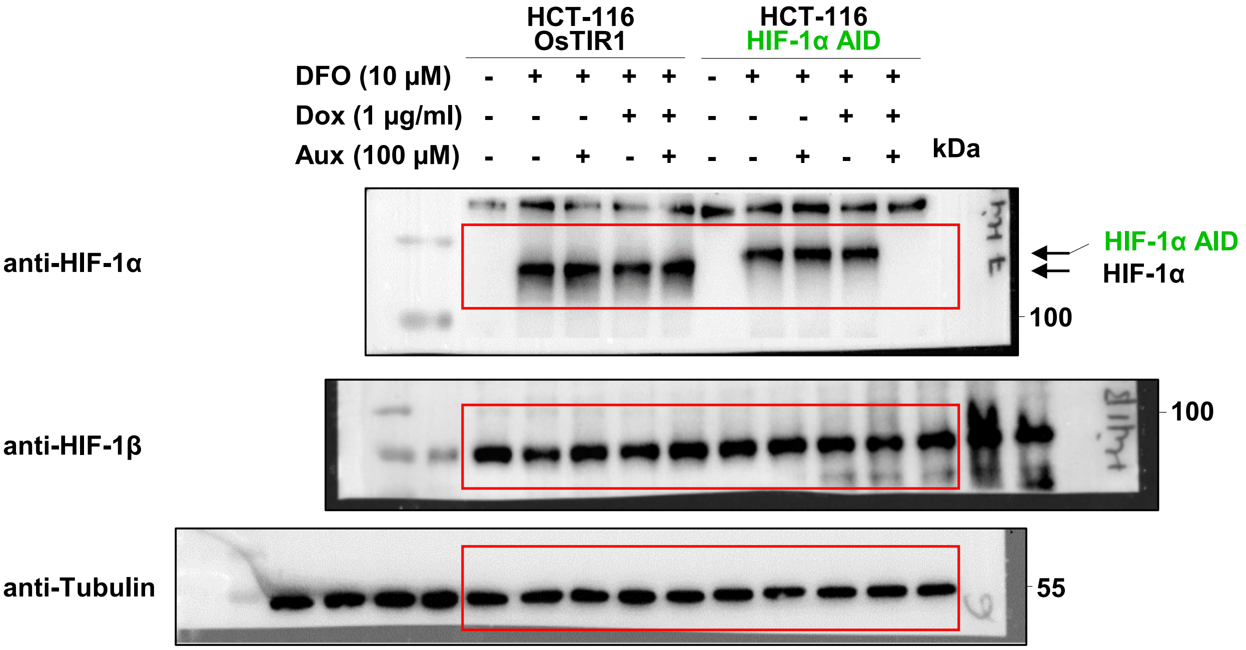
HCT-116
OsTIR1
HCT-116
HIF-1α AID
DFO (10 μM) - + + + + - + + + +
Dox (1 μg/ml) - - - + + - - - + +
Aux (100 μM) - - + - + - - + - +
kDa
anti-HIF-1α
HIF-1α AID
HIF-1α
100
anti-HIF-1β
100
anti-Tubulin
55

Fig. 3A

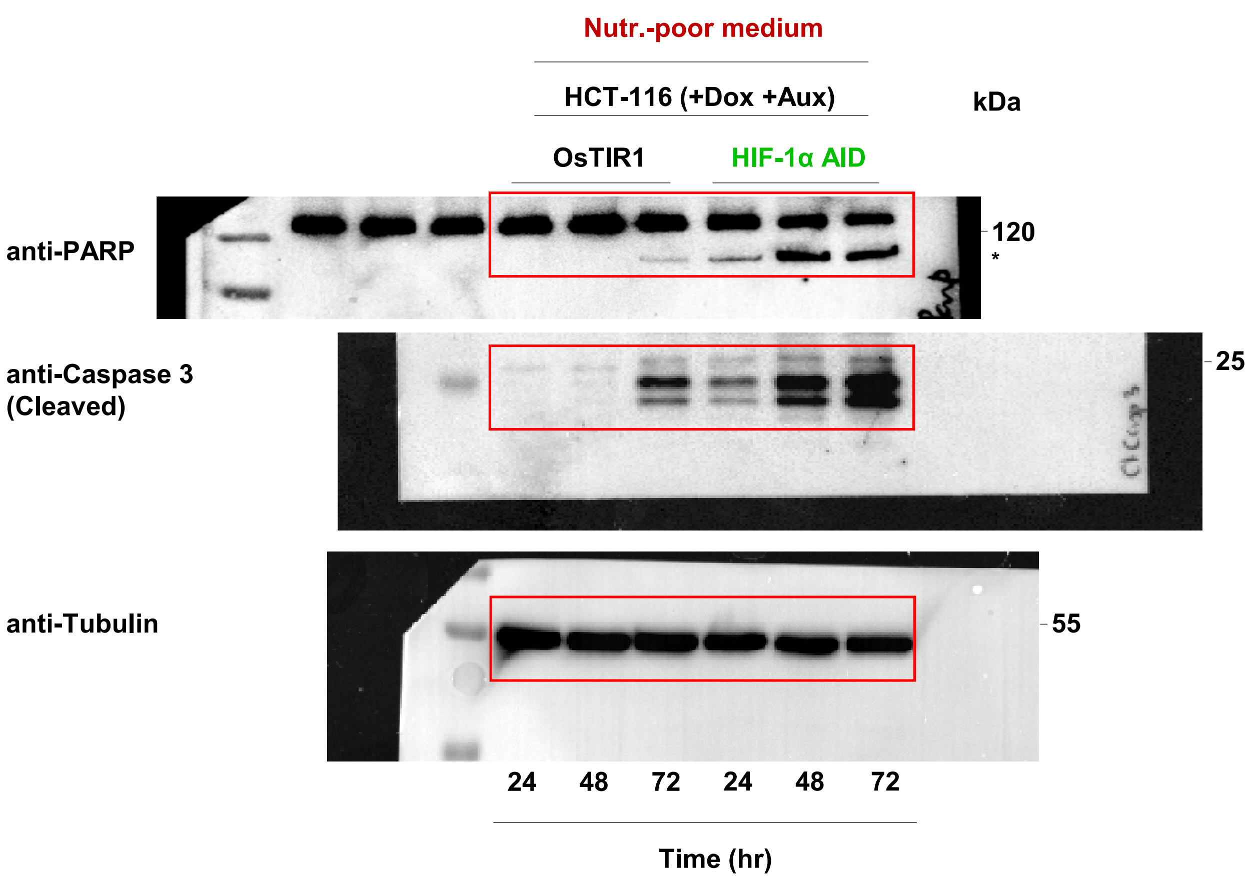

Nutr.-poor medium
HCT-116 (+Dox +Aux)
kDa
OsTIR1
HIF-1α AID
anti-PARP
120
*
anti-Caspase 3 (Cleaved)
25
anti-Tubulin
55
24
48
72
24
48
72
Time (hr)

Fig. 7C

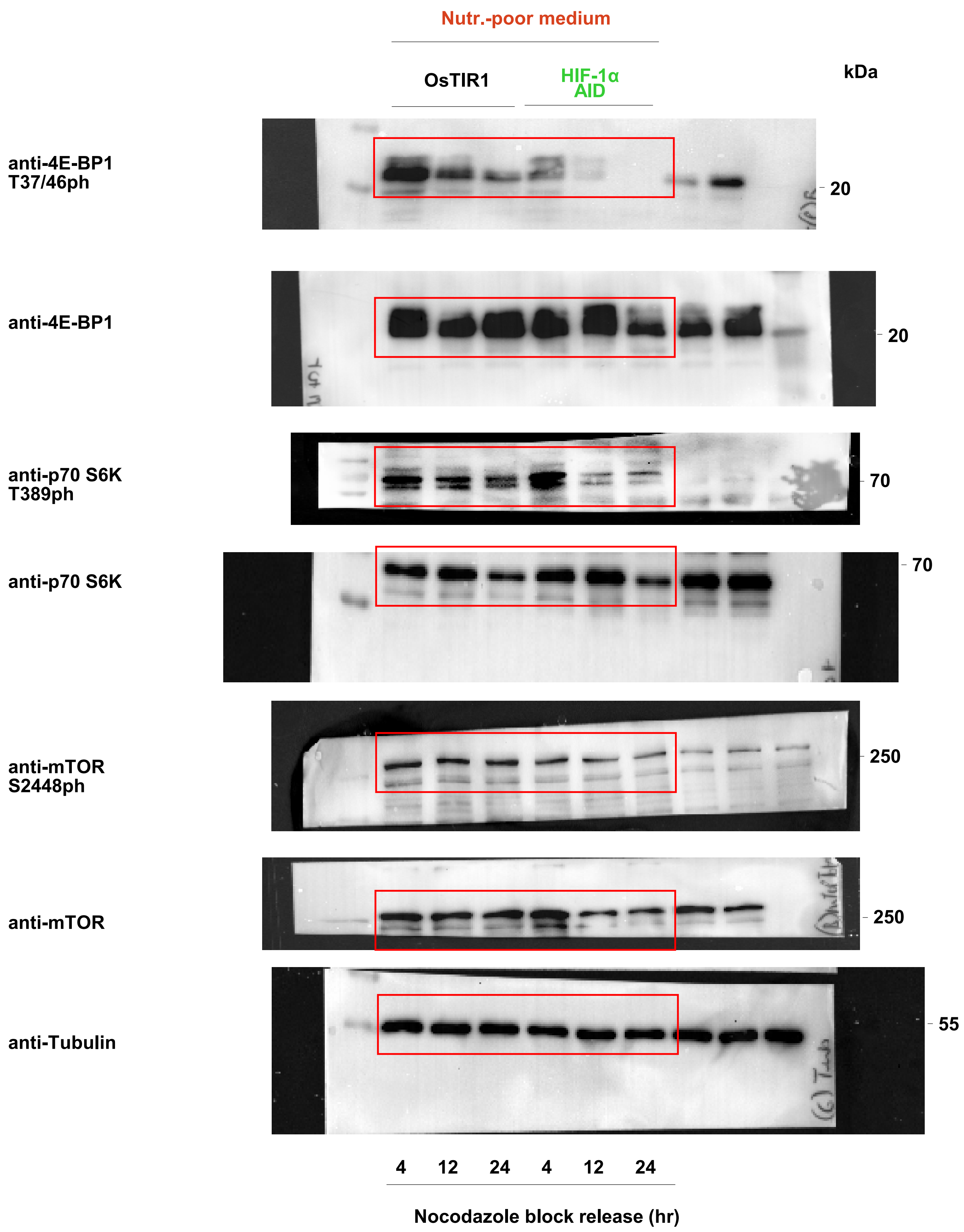
Nutr.-poor medium
OsTIR1
HIF-1α AID
kDa
anti-4E-BP1 T37/46ph
20
anti-4E-BP1
20
anti-p70 S6K T389ph
70
anti-p70 S6K
70
anti-mTOR S2448ph
250
anti-mTOR
250
anti-Tubulin
55
4
12
24
4
12
24
Nocodazole block release (hr)

Fig. 8D

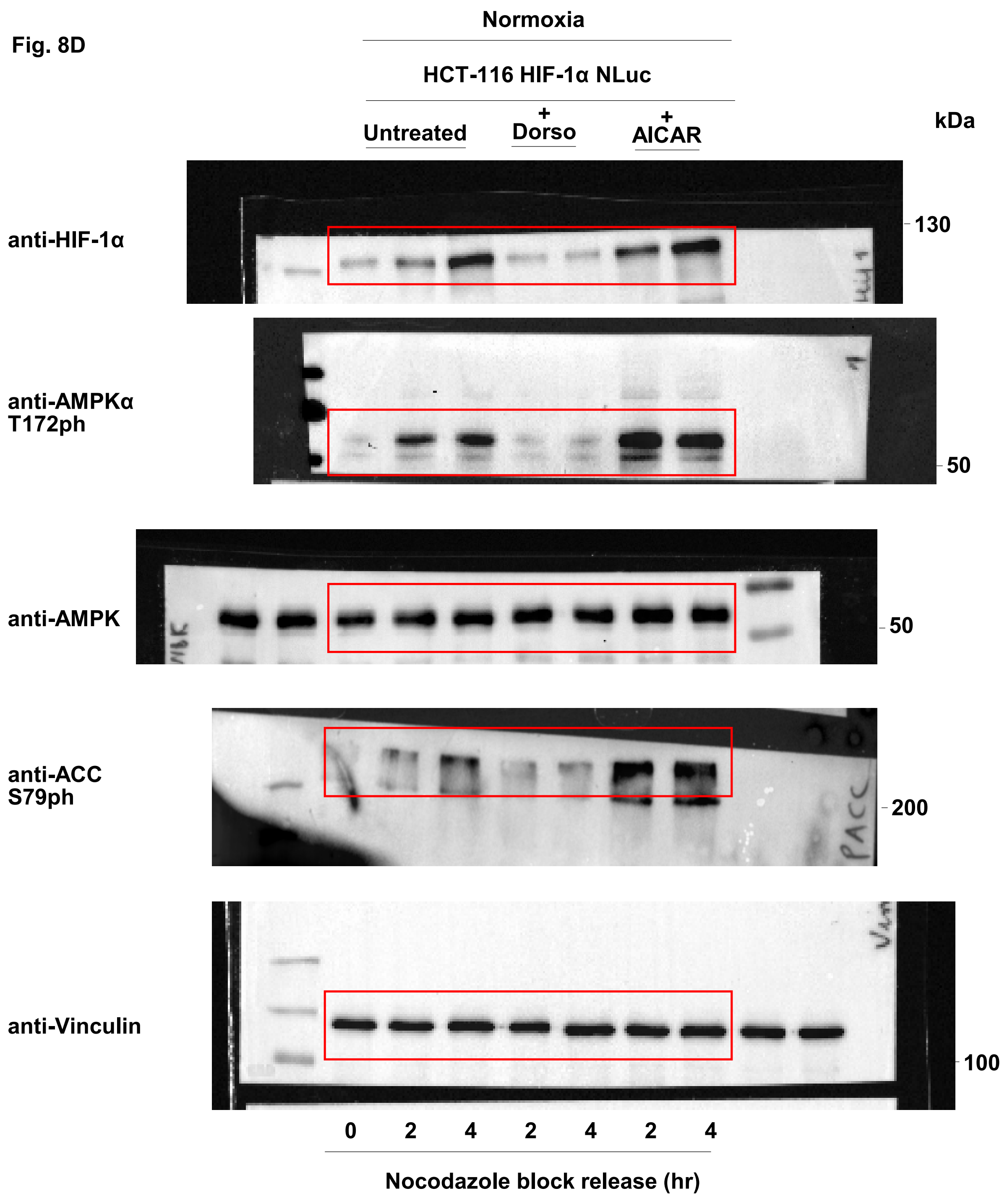
Normoxia
HCT-116 HIF-1α NLuc
Untreated
+ Dorso
+ AICAR
kDa
anti-HIF-1α
130
anti-AMPKα T172ph
50
anti-AMPK
50
anti-ACC S79ph
200
anti-Vinculin
100
0 2 4 2 4 2 4
Nocodazole block release (hr)

Fig. 8F

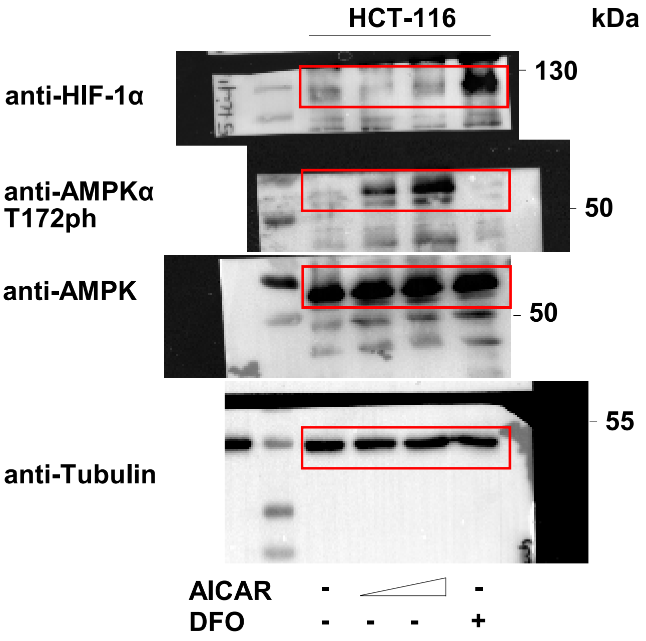
HCT-116
kDa
anti-HIF-1α
130
anti-AMPKα T172ph
50
anti-AMPK
50
anti-Tubulin
55
AICAR - -
DFO - - - +

Fig. 8G

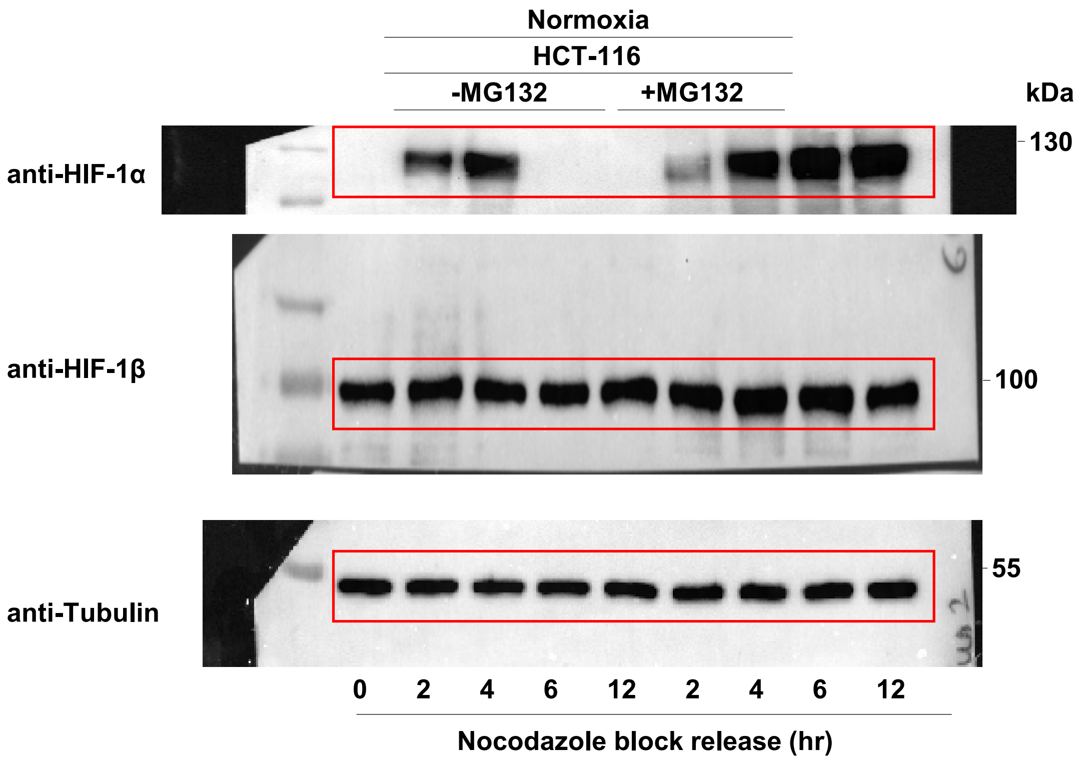
Normoxia
HCT-116
-MG132
+MG132
kDa
anti-HIF-1α
130
anti-HIF-1β
100
anti-Tubulin
55
0
2
4
6
12
2
4
6
12
Nocodazole block release (hr)

Supplementary Figures

Suppl. Fig. S2D

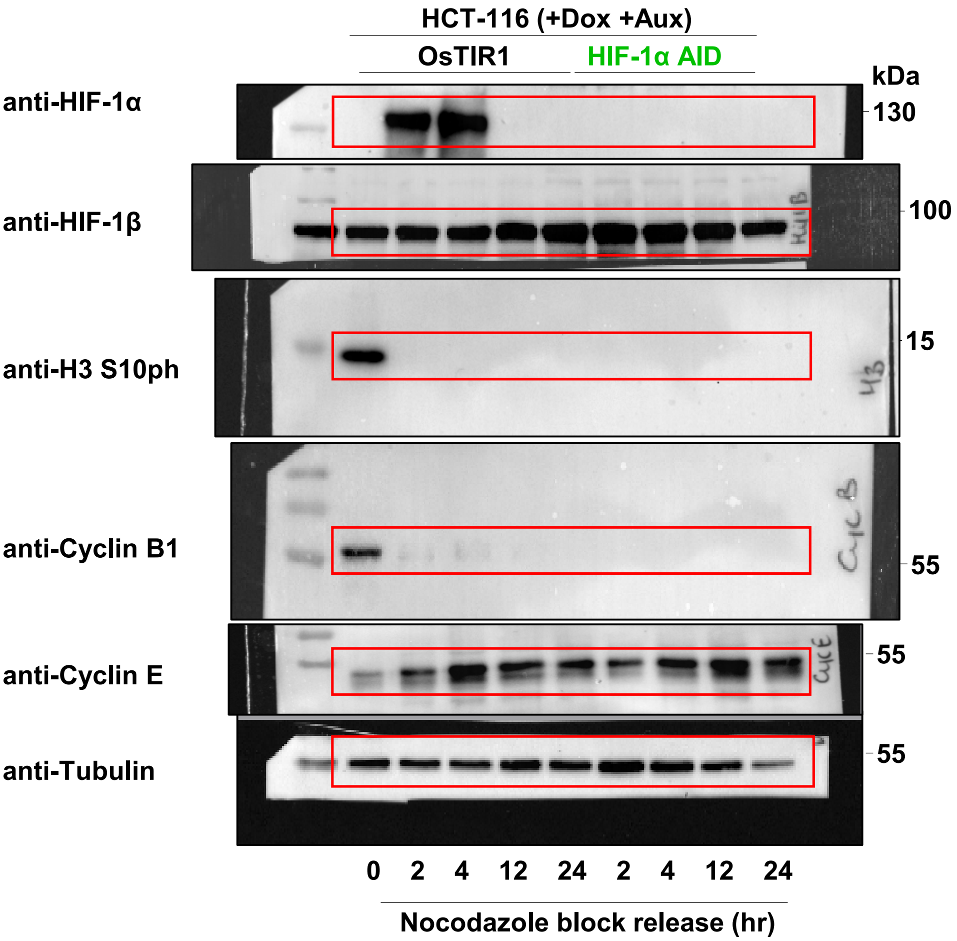

Suppl. Fig. S2E

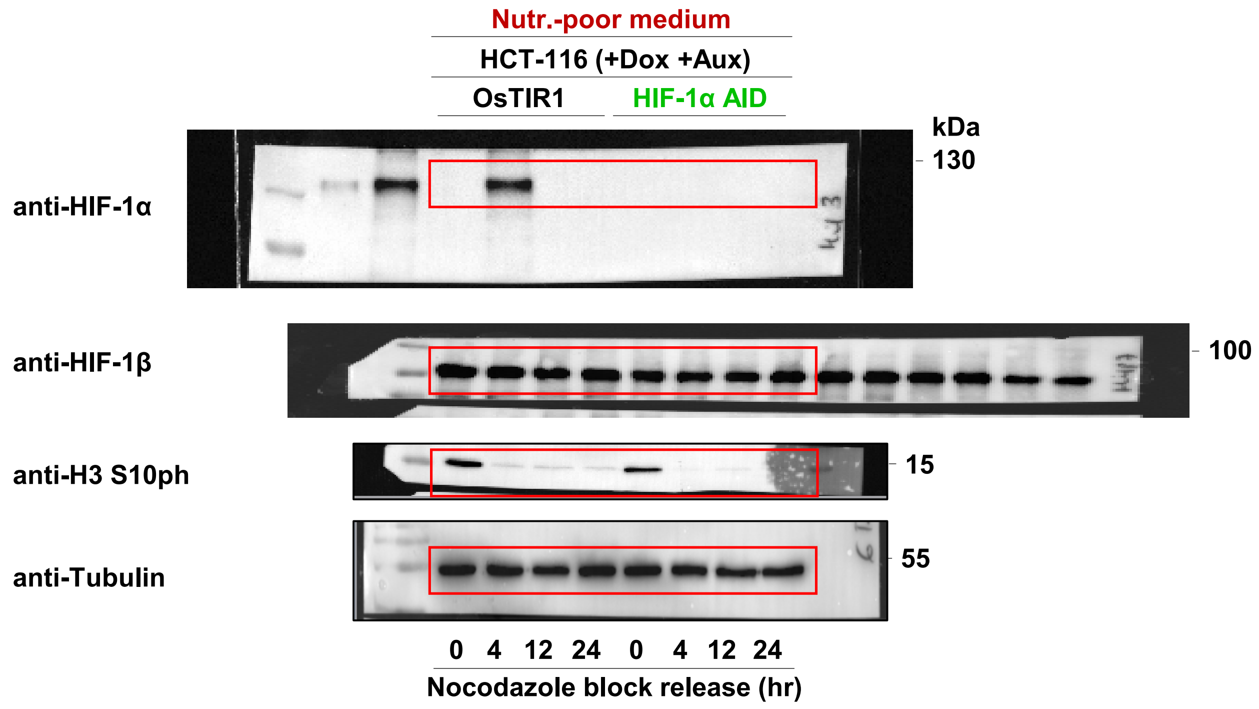

Suppl. Fig. S2 F

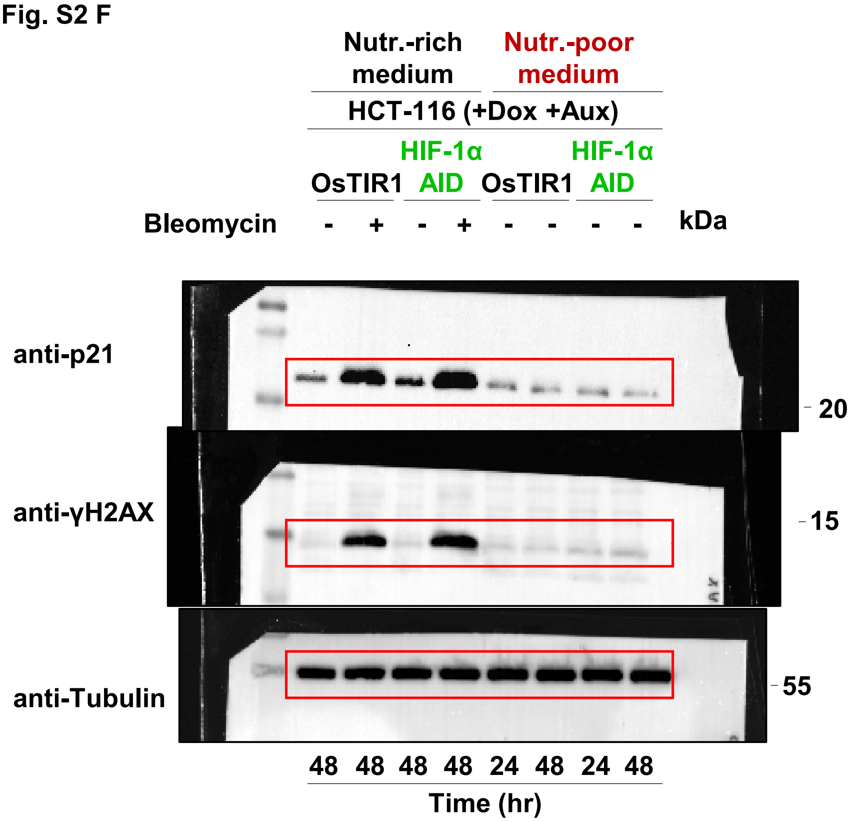

Suppl. Fig. S2G

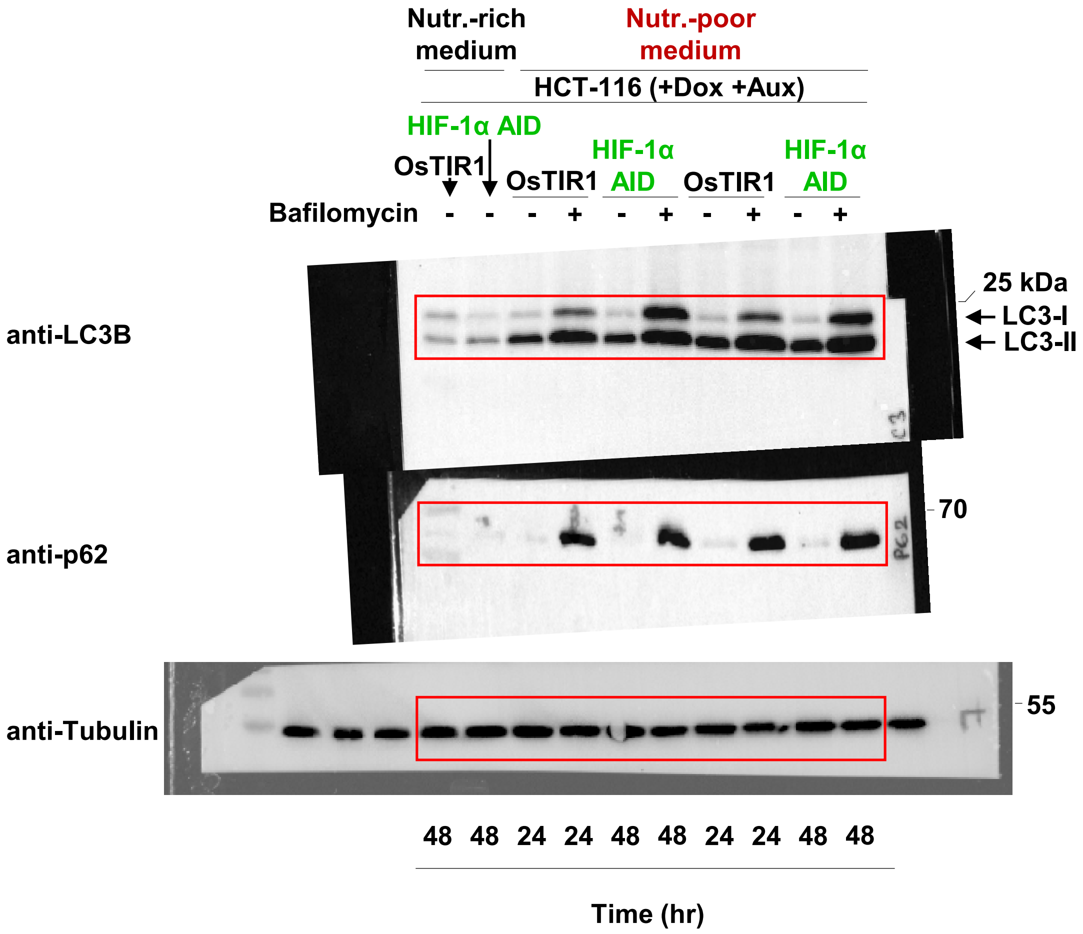

Suppl. Fig. S4 A

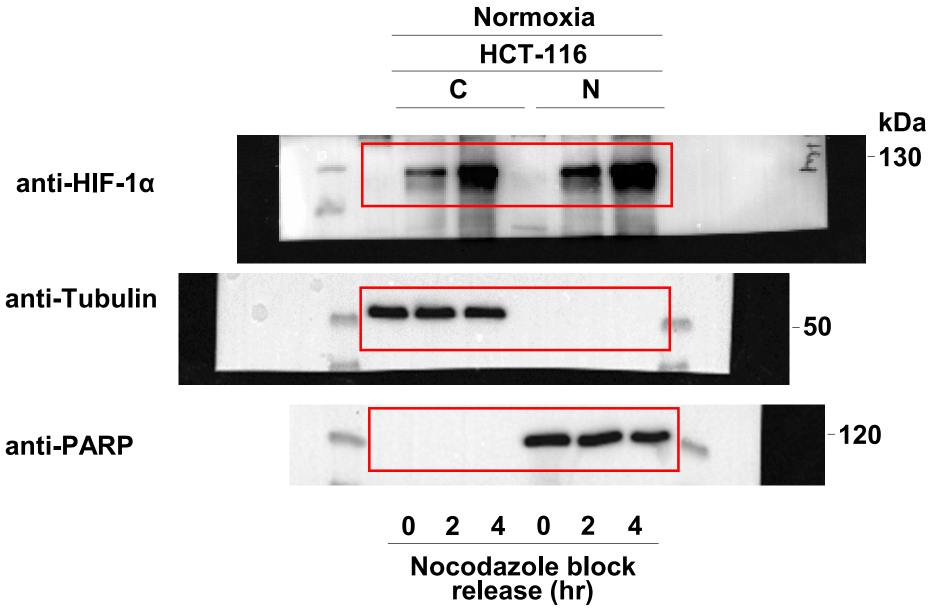

Suppl. Fig. S5

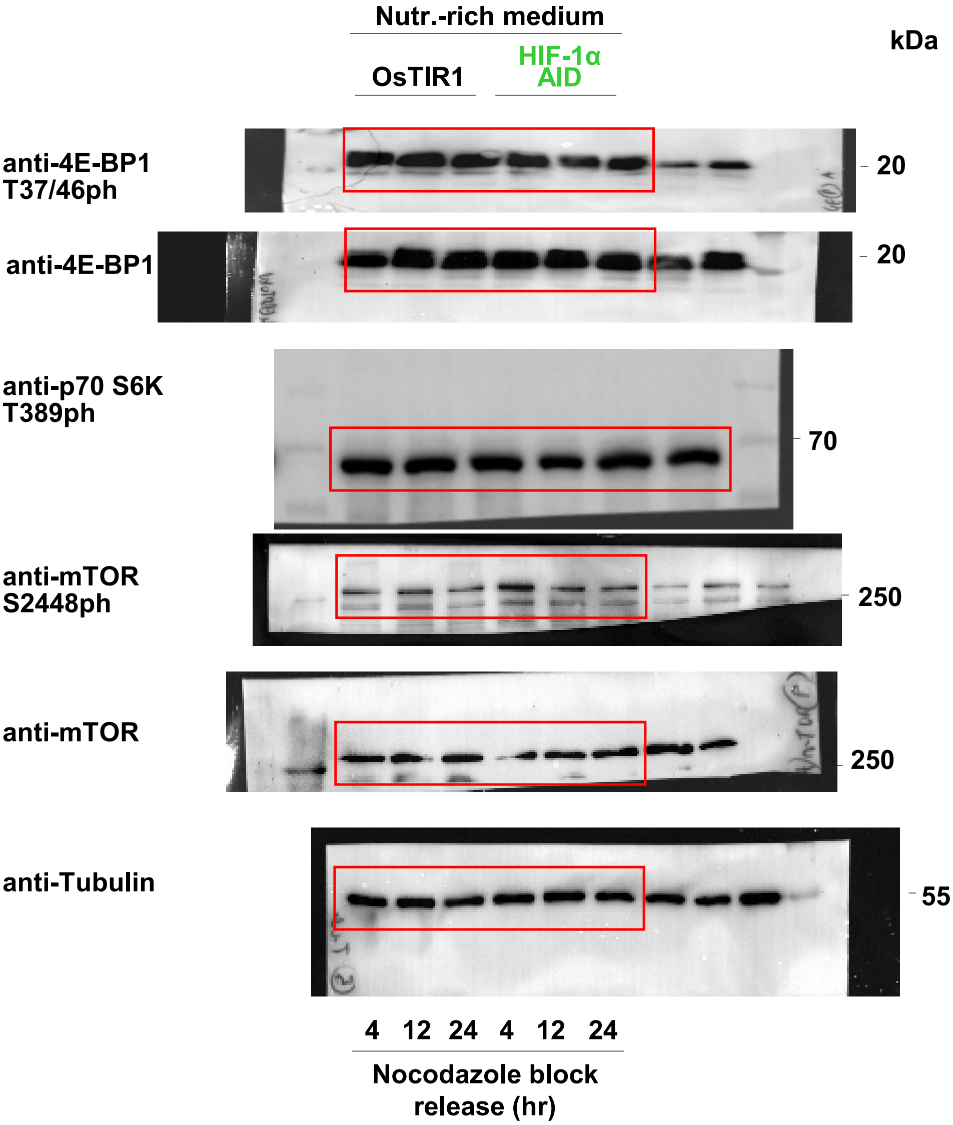

Nutr.-rich medium
OsTIR1
HIF-1α AID
kDa
anti-4E-BP1 T37/46ph
20
anti-4E-BP1
20
anti-p70 S6K T389ph
70
anti-mTOR S2448ph
250
anti-mTOR
250
anti-Tubulin
55
4 12 24 4 12 24
Nocodazole block release (hr)

Suppl. Fig. S6B Upper

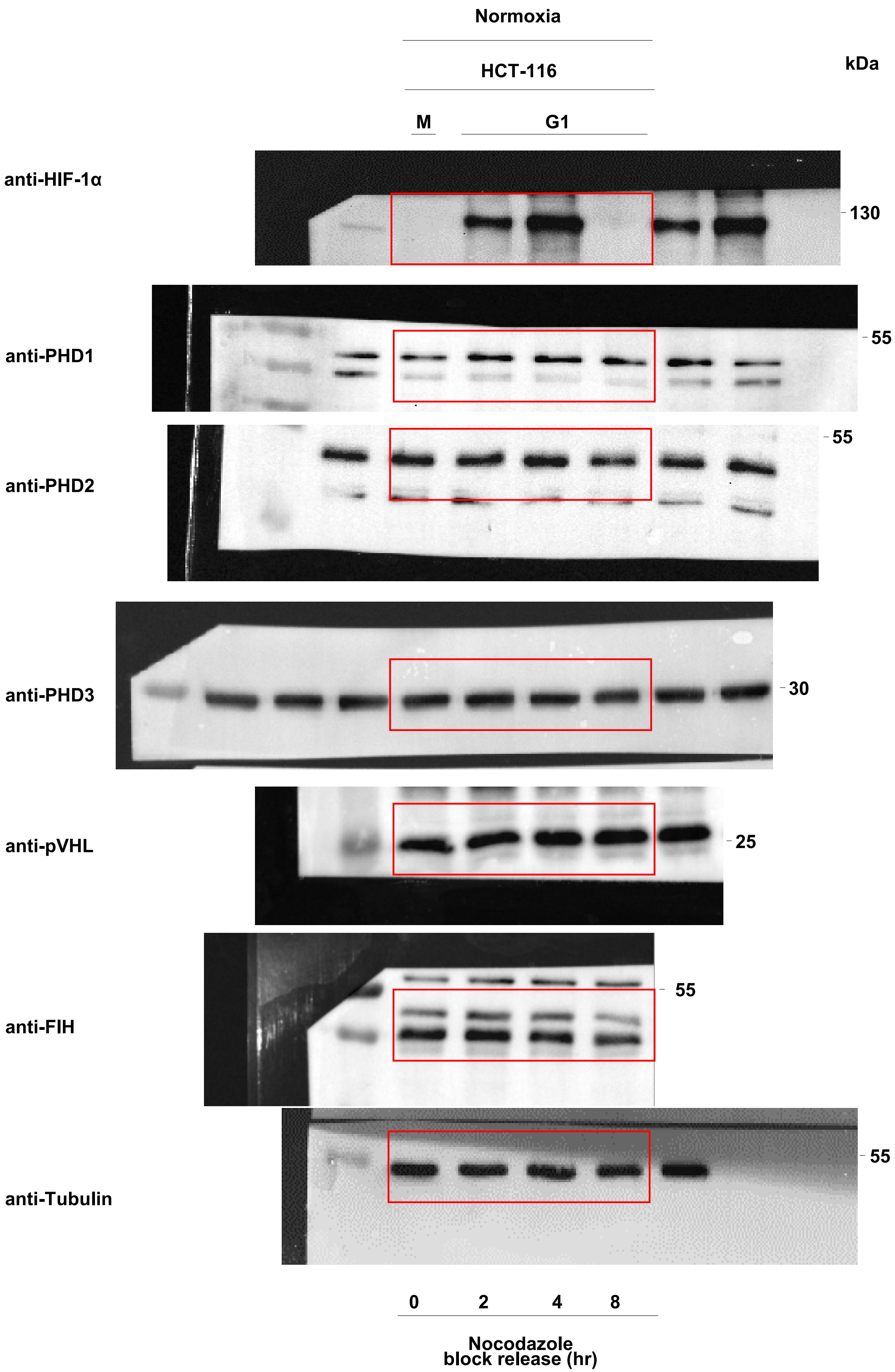

Suppl. Fig. S6B Lower

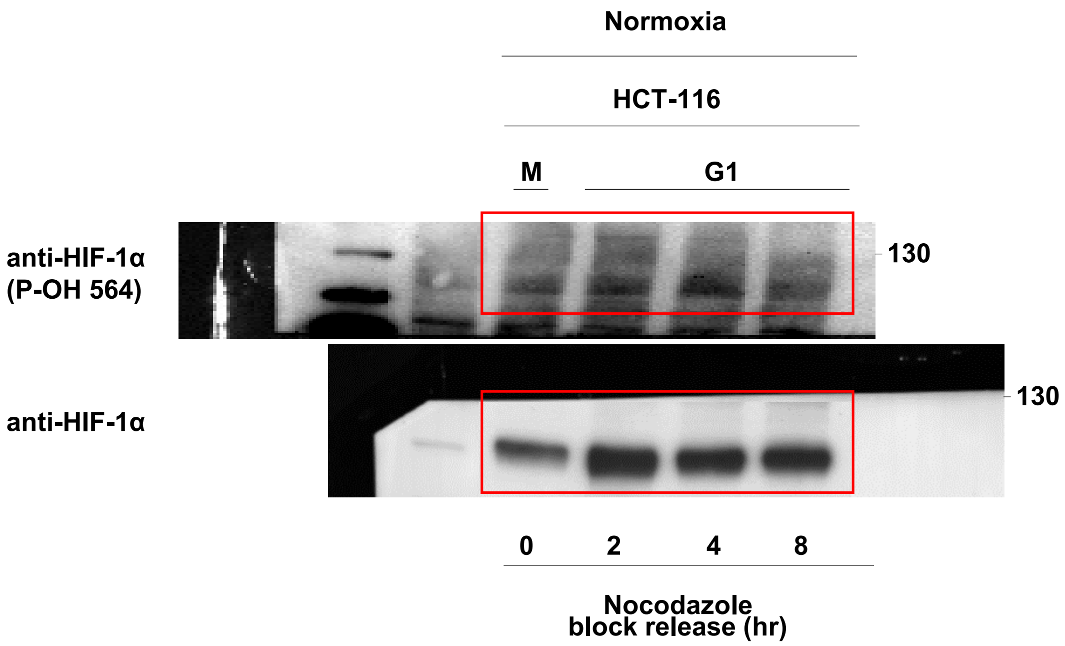

Suppl. Fig. S6C

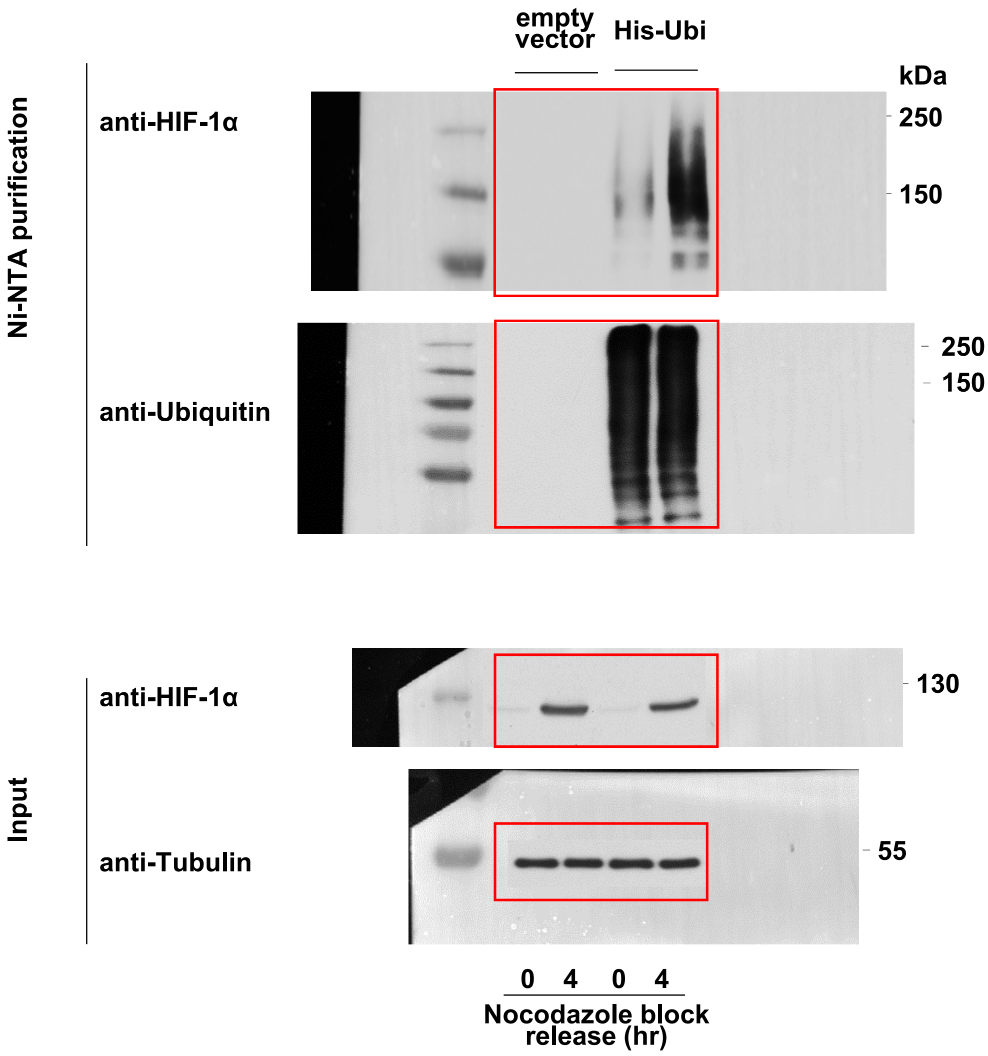

Suppl. Fig. S7A

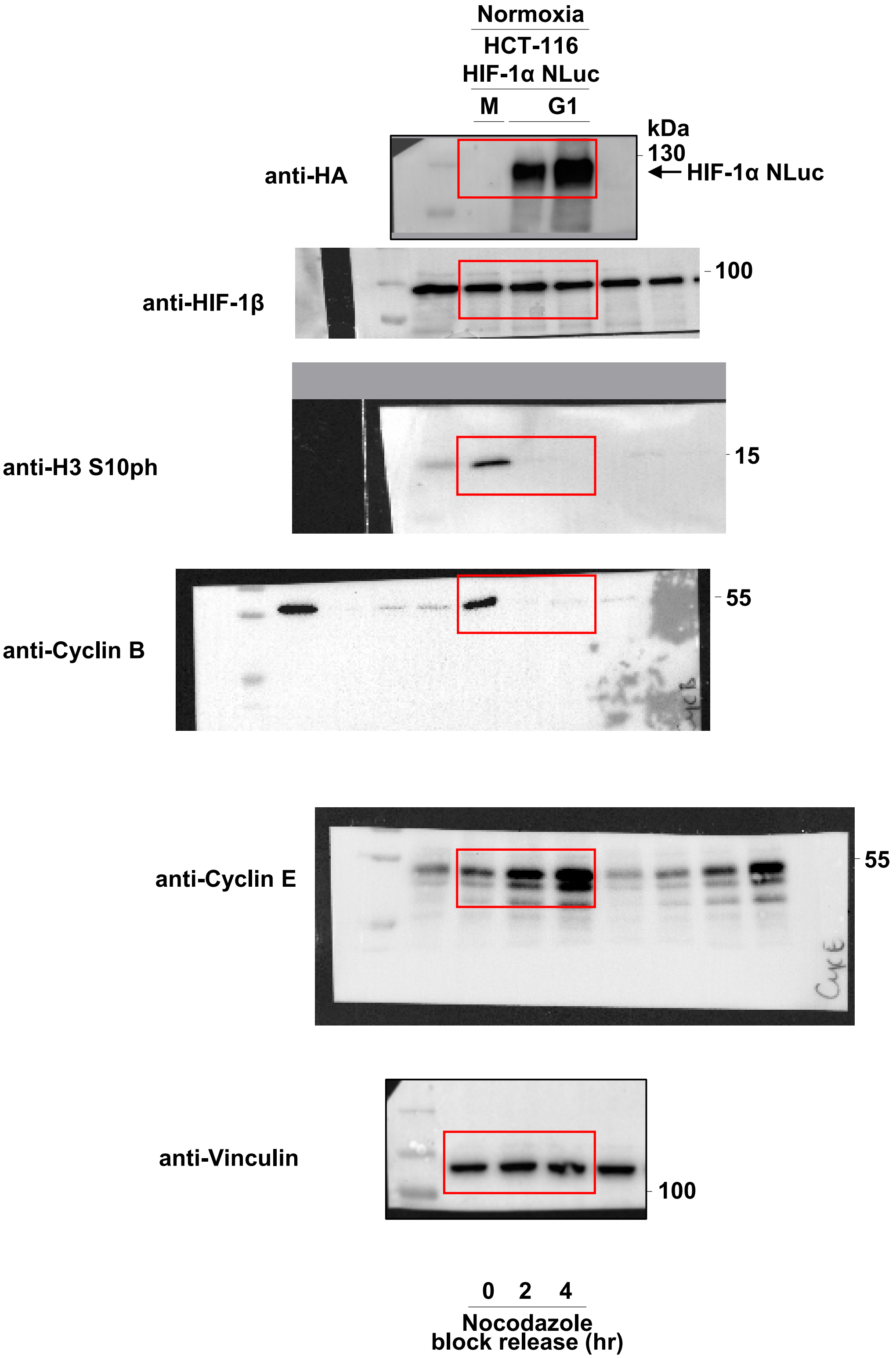

Suppl. Fig. S7C

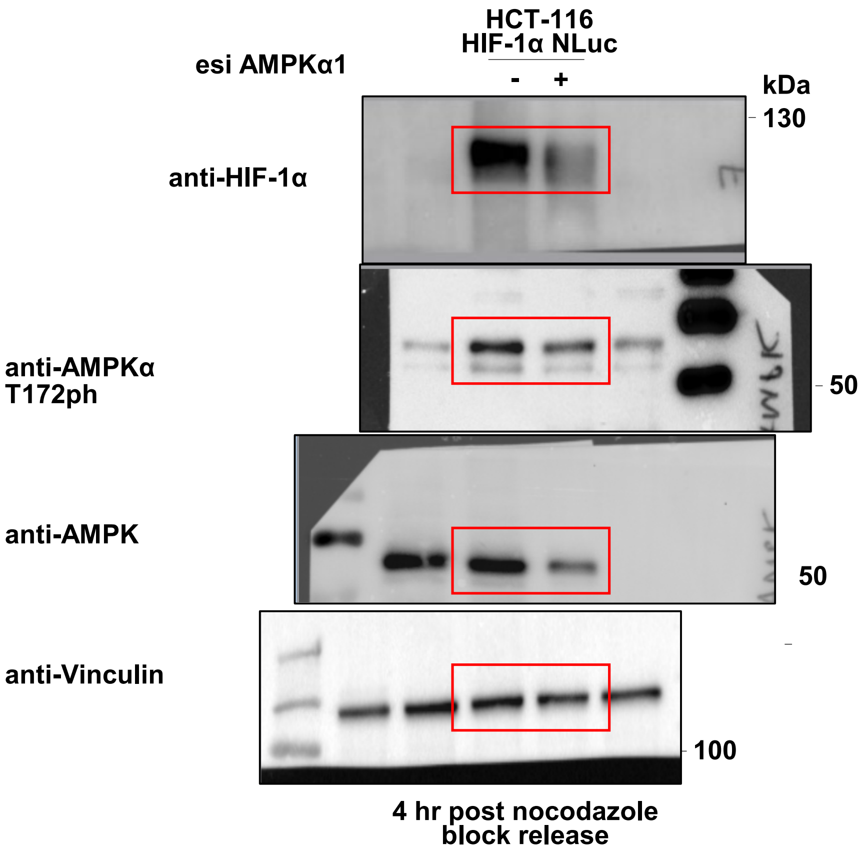

Suppl. Fig. S7D Left

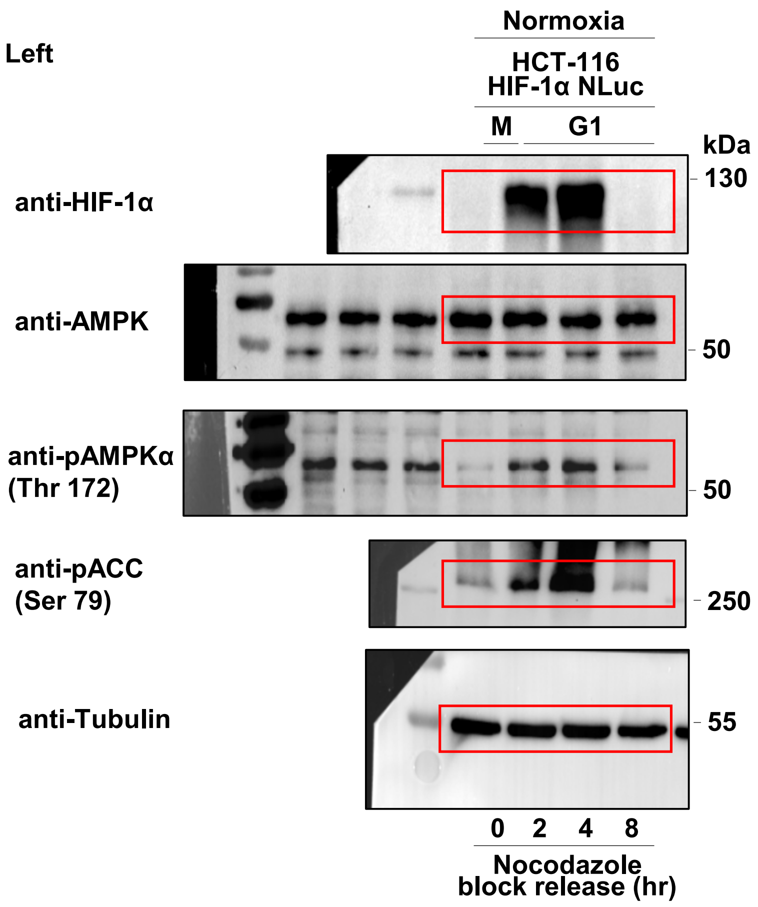

Suppl. Fig. S7D Right

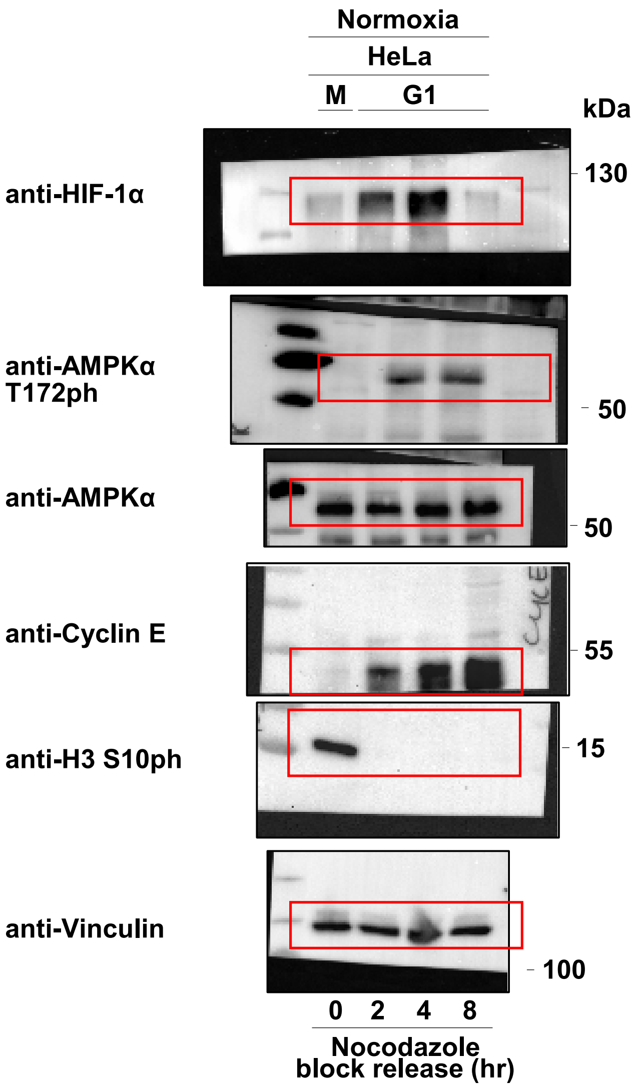

Suppl. Fig. S8A

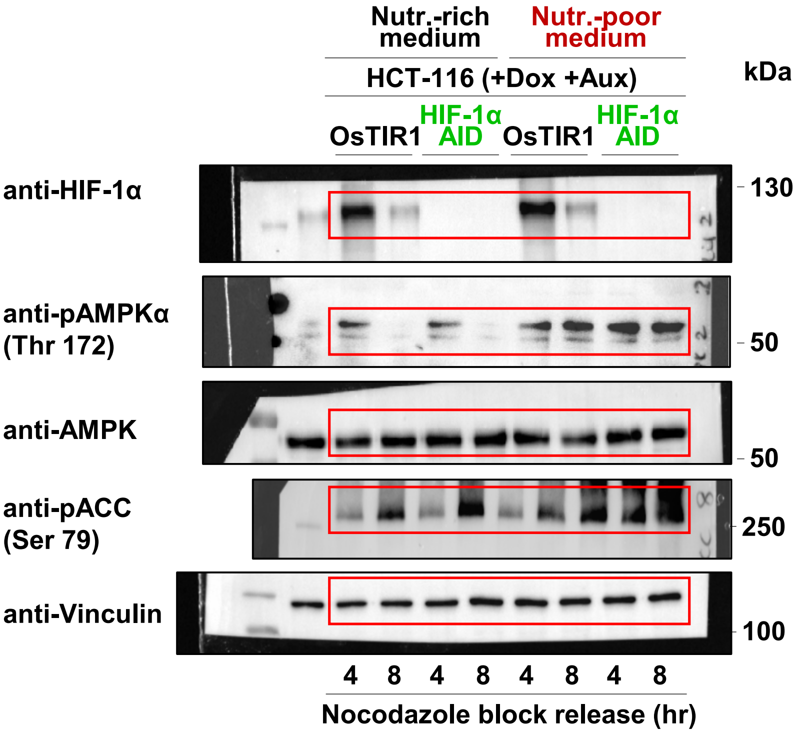

Suppl. Fig. S8B

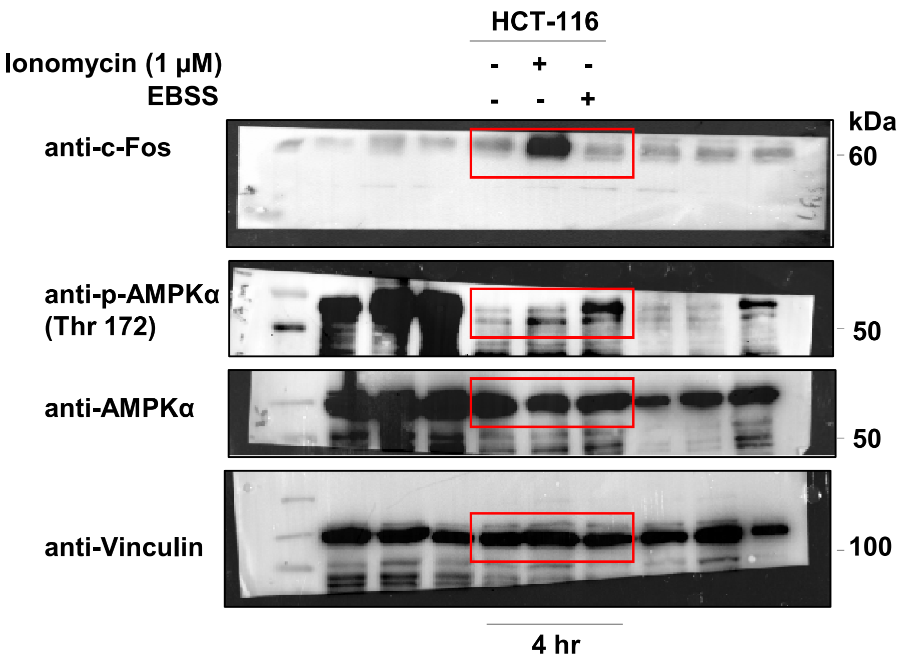

Supplement: Supplementary file 7 — uncropped blots [file 41419_2023_6012_MOESM7_ESM.pdf]
